# Effects of a slowly fermentable fiber mixture against the background of a high-protein diet on insulin sensitivity and metabolic health in individuals with overweight: a randomized, placebo-controlled trial

**DOI:** 10.1080/19490976.2025.2606473

**Published:** 2025-12-29

**Authors:** Colin A. J. van Kalkeren, Thirza van Deuren, Miranda M. J. Coenjaerds, Gianluca Galazzo, David J.M. Barnett, John Penders, Bolette Hartmann, Jens J. Holst, Emanuel E. Canfora, Ellen E. Blaak

**Affiliations:** a Department of Human Biology, NUTRIM ‘Institute of Nutrition and Translational Research in Metabolism’, Maastricht University Medical Center+, Maastricht, The Netherlands; b Department of Nutrition and Diet, Maastricht University Medical Center+, Maastricht, The Netherlands; c Department of Medical Microbiology, NUTRIM ‘Institute of Nutrition and Translational Research in Metabolism’, Maastricht University Medical Center+, Maastricht, The Netherlands; d Department of Biomedical Sciences, NNF Center for Basic Metabolic Research, The Panum Institute, University of Copenhagen, Copenhagen, Denmark

**Keywords:** Dietary fiber, insulin resistance, cardiometabolic health, microbiome, fermentation

## Abstract

The gut microbiota ferments dietary fibers, producing short-chain fatty acids (SCFA). Enhanced SCFA production in the distal colon has been linked to improved cardiometabolic health. However, most fibers are fermented proximally, resulting in increased protein fermentation distally, producing metabolites putatively harmful to metabolic health. This 12-week randomized, placebo-controlled trial aimed to improve metabolic health through increasing distal SCFA production while inhibiting proteolytic fermentation using a fiber supplement that increased distal SCFA production *in vitro*. We assessed the effects of daily potato fiber/sugar beet pectin supplementation (fiber, *n* = 19) versus maltodextrin (placebo, *n* = 21), both added to a high-protein diet (25E% protein, ±45% plant-based), on peripheral insulin sensitivity (IS) in adults with overweight/obesity. Secondary outcomes included tissue-specific IS, body composition, microbial composition and functionality, substrate metabolism, and gut permeability. Peripheral IS tended to decrease after fiber supplementation compared to placebo (*p* = 0.081), while whole-body IS significantly decreased (*p* = 0.034). Fiber mitigated the increase in insulin-mediated carbohydrate oxidation (*p* = 0.027) and decrease in fat oxidation (*p =* 0.006) that occurred in the placebo group. Additionally, fiber prevented an increase in protein oxidation (*p* = 0.048), while increasing colonic gut permeability (*p* = 0.046) and plasma interleukin-6 (*p* = 0.025). Body composition, microbial composition, and fecal and circulating metabolites remained unchanged. In conclusion, fibers combined with a high-protein diet reduced (peripheral) IS and decreased metabolic flexibility compared to placebo. Reduced protein oxidation after fiber may reflect diminished amino acid bioavailability. Additionally, coadministration of fiber and protein may compromise gut barrier function and inflammatory responses. More research investigating the interplay between dietary fibers and proteins is needed.

## Introduction

The rising prevalence of obesity and obesity-related diseases, including type 2 diabetes (T2D), poses an increased burden on healthcare systems worldwide. Accumulating evidence suggests that the microbiome may play a prominent role in these diseases,^1-5^ being responsible for the fermentation of undigested food in the colon, including dietary fibers and proteins. Fermentation of dietary fibers produces short-chain fatty acids (SCFA), which can positively affect cardiometabolic health, improving multiorgan insulin resistance and reducing inflammation, among others.^1-3^^,^^6-8^ However, fibers become depleted towards the distal colon, initiating proteolytic fermentation instead, which produces a wide range of microbial metabolites, including branched-chain fatty acids (BCFA), ammonia, *p*-cresol, and hydrogen sulfide. These metabolites are often associated with detrimental health effects, including reduced gut barrier function, diminished lipid oxidative capacity, and increased inflammation.^2^

Previous studies have shown that acute distal, but not proximal, colonic SCFA administration induces increased lipid oxidation and reduced plasma inflammatory markers,^3^^,^^6^ suggesting that interventions increasing distal saccharolytic fermentation, and potentially inhibiting proteolytic fermentation, may alleviate cardiometabolic disturbances.^1^^,^^9^ However, not every fiber induces distal SCFA production. Fermentable fibers with a low molecular mass and a less complex structure (e.g., oligosaccharides) are rapidly fermented in the proximal colon, whereas more complex fibers might be fermented slowly and thereby reach the distal colon. Thus, combining dietary fibers with different fermentation rates may promote saccharolytic fermentation distally, as was shown in a recent study, combining long-chain inulin and resistant starch, which increased distal SCFA production *in vitro*,^10^ and provided acute metabolic improvements *in vivo* in lean individuals.^10^ However, *in vitro* SCFA production by the microbiota of individuals with obesity was not increased,^10^ and no effects on metabolic health were observed *in vivo* in individuals with prediabetes. This might be explained by differences in the gut microbiome between individuals with normal weight and obesity and prediabetes,^2^^,^^11^^,^^12^ evidenced by lower microbial diversity and fewer SCFA-producing taxa in individuals with overweight, potentially with increased protein degradation capacities.^4^^,^^5^^,^^11^ Hence, metabolic phenotype, initial microbiota composition, and the microbiota's SCFA-producing capacity may be important determinants of response to nutritional interventions.^4^^,^^11-13^

Besides differences in fiber type and metabolic phenotype, combining fibers and (plant-based) proteins might influence the effects of fiber-induced steering of colonic fermentation and, subsequently, metabolic processes. Since current nutritional guidelines generally aim to shift toward plant-based diets and increased dietary fiber intake, understanding fiber–protein interactions is essential.^7^^,^^14^ While plant-based diets in general have been associated with cardiometabolic benefits,^15^ and plant-derived proteins may improve insulin sensitivity, inflammatory processes, and body weight control,^16-18^ the impact of indigestible plant-based proteins on microbial composition and functionality is still insufficiently understood. Plant-based, compared to animal-based, proteins are generally less digestible,^18^^,^^19^ potentially increasing proteolytic fermentation in the colon. Since proteolytic fermentation might already be increased in individuals with obesity, understanding the microbial and metabolic effects of combining (plant-based) proteins and dietary fibers in a long-term setting in a population at risk of developing T2D and cardiometabolic complications is essential.

In this randomized-controlled trial, we aimed to steer microbial fermentation processes in the human colon through a potato fiber/sugar beet pectin supplement, known to stimulate distal colonic SCFA production *in vitro,*^20^ within the context of a high-protein diet (±45% plant-based) in individuals at risk of developing T2D. We intended to increase saccharolytic and inhibit proteolytic fermentation in the distal colon, thereby improving metabolic health through altered microbial metabolite production, including peripheral insulin sensitivity (IS) as the primary outcome. Secondary outcomes were hepatic and adipose tissue, as well as whole-body IS, substrate oxidation, body composition, circulating cardiometabolic markers, and microbial composition and functionality as secondary outcomes.

## Methods

### Recruitment and study registration

**Figure 1.**
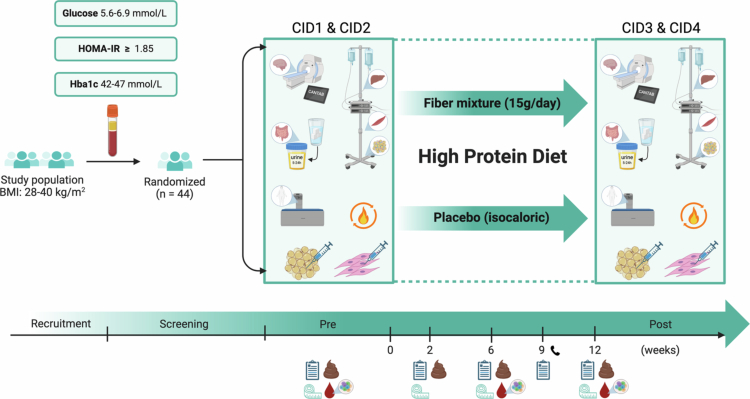
Experimental design of the DISTAL-study. After screening for eligibility, participants visited the university for two clinical investigation days (CID 1 and 2) for baseline measurements, including body composition (anthropometrics and DEXA scan), gut permeability (multisugar test), substrate metabolism (indirect calorimetry), and tissue-specific insulin sensitivity (two-step hyperinsulinemic-euglycemic clamp). All measurements were repeated after the 12-week intervention (CID 3 and 4), during which all individuals adhered to a high-protein diet and were randomized to receive either the fiber mixture or a placebo. Interim visits were scheduled at weeks 2, 6, and 9. At multiple time points, participants provided fecal samples for microbiota composition, blood samples, and questionnaires.

The DISTAL study was a 12-week, parallel, double-blind, placebo-controlled dietary intervention, investigating the effects of potato fiber and sugar beet pectin supplementation versus an isocaloric placebo, both within the context of a high-protein diet (25E% protein, ±45% plant-based), in 44 adults with overweight/obesity and impaired glucose metabolism (30–75 y, BMI 28–40 kg/m^2^, exhibiting signs of insulin resistance, based on either fasting glucose (5.6–6.9 mmol/l), HbA1c (42–47 mmol/mol), or HOMA-IR (>1.85)) (Figure 1). This single-center study was conducted between September 2022 and June 2024 at Maastricht University Medical Center+ (MUMC+, Maastricht, the Netherlands) and Maastricht University (Maastricht University, Maastricht, the Netherlands), was approved by the local Medical Ethics Committee (METC azM/UM, MUMC+, Maastricht, the Netherlands, no. NL80459.068.22), registered at ClinicalTrials.gov (NCT05354245, https://clinicaltrials.gov/study/NCT05354245), and was carried out according to the principles of the Declaration of Helsinki (October 2013), and monitored by the Clinical Trial Center Maastricht (CTCM, Maastricht, the Netherlands). Patients and/or the public were not involved in the design, conduct, reporting, or dissemination plans of this research. Participants were recruited from the vicinity of Maastricht, The Netherlands, via an existing volunteer database and online and paper media, including flyers, local newspaper advertisements, and online volunteer platforms. All participants involved in this study provided written and verbal consent after receiving information (verbally and on paper) on the study design and conducted experiments, and were provided financial compensation for their efforts. Further details on recruitment and screening for eligibility can be found in the Supplementary Materials.

### Primary and secondary outcome measures

We assessed the effects of a potato fiber and sugar beet pectin mixture, within the context of a high-protein, partially plant-based diet, versus placebo (maltodextrin) on peripheral insulin sensitivity (IS), measured by the two-step hyperinsulinemic-euglycemic clamp as our primary outcome, and on tissue-specific and whole-body IS, substrate and energy metabolism, cardiometabolic markers, microbial composition and functionality, gut permeability, and general well-being as secondary outcomes.

### Potato fiber and sugar beet pectin supplementation

The investigated fiber mixture was based on our previous *in vitro* experiments, in which the current combination yielded the most saccharolytic fermentation and SCFA production in the distal colon.^20^ Participants were randomized using minimization for age, sex, and BMI to either of the intervention groups (the potato fiber and sugar beet pectin mixture (fiber) or an isocaloric placebo (maltodextrin)). The fiber supplement consisted of 15 g/d (50:50 potato fiber:sugar beet pectin). Both fiber and placebo were distributed in three equal-weight sachets per day, one for each meal. Participants were instructed to start using the supplements and adhering to the high-protein diet the day after CID2 until the day before CID3, and to return all provided sachets at the end of the intervention to assess adherence.

### High-plant-protein dietary intervention

Both groups were instructed to adhere to a high-protein, partially plant-based diet. This diet consisted of 25 energy-% (E%) proteins, 30E% fats (<30% saturated fats), and 45E% carbohydrates, all in line with Dutch dietary guidelines.^21^^,^^22^ The protein content was aimed at 45% plant-based, and fiber consumption at approximately 30 g/d (fiber supplement not included). Our in-house dietician provided written and verbal instructions of the diet, which was tailored to participants' energy requirements and personal preferences to maintain weight stability and increase dietary adherence.

### Study procedures

Extensive details on study design, the dietary intervention, participant characteristics, recruitment (including a flow-chart (Supplementary figure 1)), and randomization, as well as detailed descriptions of the conducted experiments and related analyzes, can be found in the Supplementary methods. A brief description of the included experiments can be found below.

#### Tissue-specific insulin sensitivity

The two-step hyperinsulinemic-euglycemic clamp, the gold standard for measuring tissue-specific and whole-body IS, was performed after a >10 h overnight fast on CID2 and CID4, which was preceded by consumption of a standardized meal (See supplementary materials for more details). At first, a [6,6-^2^H_2_]glucose tracer is infused for 2 h, after which low-dose insulin infusion (10 mU/m^2^/min) was added for three hours to allow calculation of hepatic and adipose-tissue IS. Afterwards, insulin infusion was increased to 40 mU/m^2^/min to determine peripheral and whole-body IS (by means of the rate of glucose appearance (Ra), glucose disposal (Rd), Rd per unit of circulating insulin (Rd/I), glucose infusion rates (GIR), and M values). Insulin-mediated free fatty acid suppression was calculated at the end of the low step as a measure of adipose tissue (AT) insulin sensitivity.

#### Energy expenditure and substrate metabolism

During the last thirty minutes of each step of the clamp, open-circuit indirect calorimetry was conducted using a ventilated hood system to assess energy expenditure and substrate utilization, calculated by Weir and Frayn equations.^23^^,^^24^ 24 h urinary nitrogen excretion was incorporated to accurately determine protein oxidation. Furthermore, metabolic flexibility was assessed by calculating the difference in respiratory quotient between baseline and the high step and the switch in insulin-stimulated substrate oxidation.

#### Body composition, anthropometry, and blood pressure

Dual-energy X-ray absorptiometry (DEXA) was performed at CID1 and CID3 to determine body fat mass, fat-free mass, fat distribution, and visceral adipose tissue. Body weight, waist and hip circumferences, and blood pressure were measured on CID1, weeks 2 and 6, and CID3.

#### Cardiometabolic markers

Fasting blood samples were obtained at CID2 and CID4, and during the week-6 visit after an overnight fast to assess cardiometabolic markers, including glucose, insulin, glycated hemoglobin A1 (HbA_1c_), free-fatty acids (FFA), inflammatory markers (interleukin (IL)-6, IL-8, IL-10, tumor necrosis factor-*α* (TNA-*α*), interferon-*γ* (IFN-*γ*), C-reactive protein (CRP), and leukocytes), lipid profile, lipopolysaccharide-binding protein (LBP), satiety hormones (Peptide YY (PYY) and glucagon-like peptide 1 (GLP-1)), and SCFA and BCFA.

#### Microbial composition and functionality

Fecal microbiota composition and functionality were assessed at CID1, week 2, week 6, and CID3. Composition was determined using 16S rRNA gene amplicon sequencing, while functionality was assessed through fecal SCFA and BCFA concentrations.

#### Gut permeability

A multisugar test, containing four different sugars (lactulose, sucralose, erythritol, and L-rhamnose) dissolved in water, was used to assess upper and lower gastrointestinal (GI) permeability on CID1 and CID3. Upper GI permeability was assessed by calculating the urinary lactulose/rhamnose ratio obtained within the first 5 h after ingestion, whereas lower permeability was derived from the sucralose/erythritol ratio in 5–24 h urine.

#### Self-reported data

On CID1, week 6, and CID3, dietary intake was assessed using three 3-d food records from three random days (two weekdays, one weekend day), and the Short Questionnaire to Assess Health-enhancing physical activity (SQUASH) was filled in to assess physical activity. Information regarding gastrointestinal symptoms and stool consistency was obtained on CID1, weeks 2 and 6, and CID3 using the Gastrointestinal Symptom Rating Scale (GSRS) and Bristol Stool Scale (BSS), respectively. Additionally, general well-being (RAND-36), perceived stress (Perceived Stress Scale (PSS-10) and eating behavior (Three-Factor Eating Questionnaire (TFEQ)) were assessed on CID1 and 3.

### Statistical analysis

A power calculation (20% difference in insulin sensitivity, power of 80%, alpha of 5% and a 20% drop-out rate) indicated we needed 22 individuals per group. Per protocol analyzes were performed on all data. Continuous data were analyzed using linear mixed models with repeated measures to assess differential effects between groups over time. Categorical data were analyzed using generalized estimated equations. Nonnormally distributed data were ln-transformed. Displayed data are means ± SD, and presented *p*-values are two-tailed, adjusted for age, sex, and BMI at baseline.

Microbial alpha and beta diversity were derived from unfiltered data using the effective Shannon index, Faith's phylogenetic diversity, Aitchison distance, and generalized UniFrac distance. The centered log ratio (CLR) transformation was applied to genus abundances prior to differential abundance analyzes. The Benjamini‒Hochberg procedure was applied to correct for multiple testing and control the false discovery rate (FDR).^25^

## Results

In total, 104 people were screened for participation between September 2022 and June 2024. Fifty individuals could be included in the study (Supplementary Figure 1); however, 6 participants withdrew before CID1, leaving a total of 44 participants to be randomized to one of the intervention arms (fiber *n* = 21; placebo *n* = 23). After two dropouts in each group, 19 and 21 participants completed the fiber and placebo intervention, respectively (*see Supplementary materials – ‘Data collection summary’ for more details)*. Baseline characteristics are displayed in Table 1, which shows comparable groups overall, with a mean age of 59.6 and 58.0 y in the placebo and fiber group, respectively, and an average BMI of ±33.5 kg/m^2^ and a female percentage of ±58% in both groups. No serious adverse events occurred during the study period.

**Table 1.** Participant characteristics at baseline.

	Placebo (*n* = 21)	Fiber (*n* = 19)
*Age (y)*	59.6 ± 11.2	58.0 ± 12.1
*Female sex*	12 (57.1)	11 (57.9)
*Weight (kg)*	100.1 ± 16.8	97.6 ± 14.4
*BMI (kg/m* ^*2*^ *)*	33.5 ± 3.8	33.6 ± 4.1
*Fasting glucose (mmol/l)*	5.5 ± 0.5	5.6 ± 0.6
*HbA*_*1c*_ *(%)*	5.6 ± 0.3^*^	5.5 ± 0.3
*Insulin (pmol/l)*	93 ± 36	89 ± 57
*HOMA-IR*	3.29 ± 1.32	3.11 ± 1.95
*Hemoglobin (mmol/l)*	9.1 ± 0.8	9.0 ± 0.6
*Creatinine (mmol/l)*	74 ± 12	78 ± 20
*ALT (U/l)*	27 ± 8	28 ± 14
*AST (U/l)*	24 ± 5	24 ± 8
*SBP (mmHg)*	134 ± 11	126 ± 10
*DBP (mmHg)*	85 ± 8	84 ± 9
*Alcohol drinker*	17 (81.0)	17 (89.5)
*Units of alcohol/week*	4 (5)	2 (3)
*Smoker*	3 (14.3)	0 (0)
Diagnosis of:		
* Hypertension*	4 (19.0)	2 (10.5)
* Hypercholesterolemia*	2 (9.5)	3 (15.8)
* Hypothyroidism*	1 (4.8)	0 (0)
Use of:		
* Antihypertensive drugs*	4 (19.0)	2 (10.5)
* Statins*	1 (4.8)	2 (10.5)
* Antidepressants*	3 (14.3)	1 (5.3)
Menopausal state^#^:		
* Premenopausal*	4 (33.3)	5 (45.5)
* Postmenopausal*	8 (66.7)	6 (54.5)

Data are presented as mean ± SD for continuous variables or *n* (%) for categorical variables. *n = 20, ^#^(% of females). ALT = alanine aminotransferase, AST = aspartate aminotransferase, BMI = body mass index, DBP = diastolic blood pressure, HbA1c = hemoglobin A1c, HOMA-IR = homeostatic model assessment for insulin resistance, SBP = systolic blood pressure.

### Dietary adherence and physical activity

Protein intake, based on dietary records filled in by the participants before CID1, week 6, and CID3, was significantly increased during the intervention period in both groups to approximately 20E%, of which ±50% was plant-based (*p* < 0.001, Supplementary Table 1). Dietary intake was comparable between groups during the intervention period. Furthermore, overall supplement adherence was adequate (87.5% of the participants ingested >95% of the required sachets, 5% of the participants ingested >90%, and the remainder ingested <90%). Additionally, participants maintained their habitual physical activity throughout the study (Supplementary Table 2).

### Tissue-specific insulin sensitivity and glucose homeostasis

**Figure 2.**
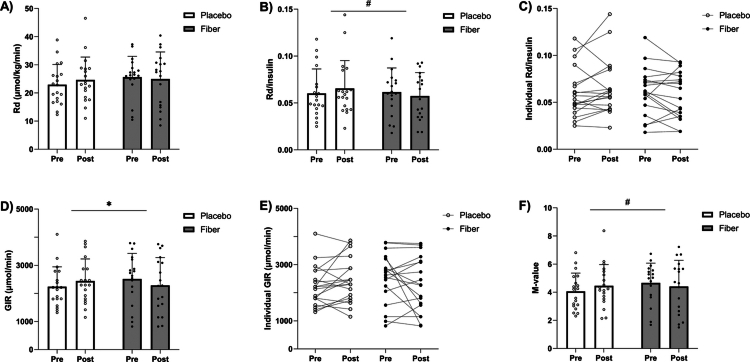
Changes in peripheral and whole-body insulin sensitivity after 12 weeks of fiber supplementation (*n* = 18) versus placebo (*n* = 19) against the background of a high-protein diet. Data are derived from the high-step of the two-step hyperinsulinemic-euglycemic clamp. Presented *p*-values indicate interaction effects. A. Rate of glucose disposal (Rd) (mean ± SD), *p* = 0.127. B. Rate of glucose disposal per unit of insulin (Rd/Insulin) (mean ± SD), *p* = 0.081. C. Individual changes in Rd/Insulin over time. D. Glucose infusion rates (GIR) (mean ± SD), *p* = 0.034. E. Individual changes in GIR over time. F. M-values (mean ± SD), *p* = 0.072.

Our primary outcome, peripheral insulin sensitivity, measured by a two-step hyperinsulinemic–euglycemic clamp, was not altered in any of the groups over time (insulin-stimulated rate of glucose disposal (Rd), *p* = 0.127, Figure 2A). Rd per unit of insulin (Rd/I), however, tended to be slightly lower in the fiber group and slightly higher in the placebo group over time (*p* = 0.081, Figure 2B/C). Accordingly, glucose infusion rates (GIR) during the high insulin infusion step of the clamp differed significantly between the groups over time, decreasing after fiber and increasing after placebo supplementation (*p* = 0.034, Figure 2D/E). A similar trend was observed in whole-body insulin sensitivity (M-value, *p* = 0.072, Figure 2F). Hepatic and adipose tissue insulin sensitivity was not different between groups or over time (Supplementary Table 3). Before and after the intervention, no differences were found in markers of glucose metabolism between the groups, including fasting glucose and insulin, HbA_1c_, and HOMA-IR (Table 2). Overall, fiber supplementation added to a high-protein diet appeared to decrease, rather than improve, insulin sensitivity compared to a high-protein diet alone.

**Table 2.** Changes in plasma cardiometabolic and inflammatory markers after 12 weeks of fiber or placebo supplementation, combined with a high-protein diet.

	Placebo (*n* = 21)		Fiber (*n* = 19)		*p*-value		
	Pre	Post	Pre	Post	Group	Time	Group*Time
*Fasting glucose (mmol/l)* *^#^*	5.70 ± 0.41	5.81 ± 0.39	5.80 ± 0.68	5.80 ± 0.55	0.653	0.351	0.346
*Fasting insulin (pmol/l)*^#^**	41.84 ± 23.86	44.52 ± 30.19	50.75 ± 44.09	59.19 ± 61.66	0.395	0.176	0.479
*HbA*_*1c*_ *(%)*	5.6 ± 0.4	5.6 ± 0.3	5.5 ± 0.4	5.5 ± 0.3	0.350	0.831	0.467
*HOMA-IR*	1.50 ± 0.81	1.67 ± 1.14	1.86 ± 1.51	2.26 ± 2.39	0.310	0.120	0.510
*Leukocytes (x10*^9^ *cells)*	6.29 ± 1.55	6.14 ± 1.01	5.54 ± 1.40	6.05 ± 1.84	0.147	0.064	0.066
*TNF-α (pg/ml)*	2.18 ± 0.51	2.00 ± 0.34	2.20 ± 0.60	2.20 ± 0.61	0.352	0.190	0.218
*IFN-γ (pg/ml)*	11.26 ± 8.89	9.18 ± 5.11	9.20 ± 7.05	8.34 ± 3.39	0.458	0.203	0.593
*IL-6 (pg/ml)*	1.41 ± 1.19	1.13 ± 1.08	0.99 ± 0.70	1.11 ± 0.95	0.531	0.348	0.025^*^
*IL-8 (pg/ml)*	8.15 ± 3.01	7.66 ± 2.92	7.49 ± 2.70	8.00 ± 3.33	0.916	0.980	0.326
*IL-10 (pg/ml)*	0.61 ± 1.19	0.59 ± 1.24	0.29 ± 0.20	0.25 ± 0.07	0.235	0.383	0.626
*CRP (pg/ml)*	3.89 ± 3.92	2.79 ± 2.04	2.53 ± 3.28	2.00 ± 1.95	0.192	0.162	0.621
*Total cholesterol (mmol/l)* *^#^*	5.00 ± 1.04	4.94 ± 0.98	4.90 ± 1.10	4.79 ± 0.97	0.681	0.263	0.758
*HDL-cholesterol (mmol/l)^#^*	1.02 ± 0.28	1.03 ± 0.27	1.05 ± 0.25	1.03 ± 0.25	0.808	0.706	0.314
*LDL-cholesterol (mmol/l)^#^*	3.08 ± 0.80	3.07 ± 0.86	3.13 ± 1.00	3.01 ± 0.78	0.989	0.315	0.480
*Triglycerides (mmol/l)^#^*	2.01 ± 0.98	2.09 ± 1.54	1.68 ± 1.10	1.84 ± 1.35	0.230	0.777	0.323
*Cholesterol/HDL ratio^#^*	5.10 ± 1.31	5.00 ± 1.27	4.91 ± 1.59	4.96 ± 1.76	0.731	0.832	0.498
*Fasting PYY (pmol/l)*	6 ± 3	6 ± 2	6 ± 4	5 ± 3	0.995	0.401	0.401
*Fasting GLP-1 (pmol/l)*	9 ± 4	8 ± 4	7 ± 4	9 ± 8	0.407	0.638	0.229
*PYY t = 330 (pmol/l)*	4 ± 2	4 ± 3	3 ± 2	3 ± 2	0.104	0.638	0.552
*GLP-1 t = 330 (pmol/l)* ^*$*^	6 ± 4	6 ± 3	5 ± 4	6 ± 6	0.330	0.719	0.965

Data are presented as mean ± SD. CRP = C-reactive protein, GLP-1 = glucagon-like peptide 1, HDL = high-density lipoprotein, HOMA-IR = Homeostatic model assessment for insulin resistance, IFN-γ = interferon γ, IL = interleukin, LDL = low-density lipoprotein, PYY = peptide YY, t = 330 = high-step of 2-step hyperinsulinemic-euglycemic clamp, TNF-α = tumor necrosis factor α. *p-value <0.05, ^#^Fiber *n* =  18, ^$^Placebo *n* = 20 and Fiber *n* = 17.

### Blood lipid spectrum, blood pressure, and body composition

Circulating markers of lipid metabolism remained unaffected after 12 weeks of either fiber or placebo intake (Table 2). Furthermore, even though systolic blood pressure was lower in the fiber group throughout the intervention (*p* = 0.017, Supplementary Table 4), no changes occurred in both systolic and diastolic blood pressure over time. Additionally, no changes in body weight, fat and lean mass, fat percentage, android/gynoid ratio, visceral adipose tissue, or waist-to-hip ratio were observed between the groups throughout the intervention, as assessed by anthropometric measurements and DEXA (Supplementary Table 4). Since the prescribed diets were isocaloric, aimed at weight maintenance to prevent interference with outcome parameters, body weight results indicate that weight maintenance was successfully achieved.

### Energy expenditure and substrate metabolism

Insulin-mediated substrate oxidation, assessed using ventilated hood measurements during the high-step of the clamp, remained relatively unaltered in the fiber group, while both RQ and CHO oxidation increased over time in the placebo group (*p* = 0.010 and *p* = 0.027, respectively, Table 3). Accordingly, insulin-mediated suppression of fat oxidation was less pronounced in the fiber group compared to placebo (*p* = 0.006). These data indicate that the high-protein diet without the fiber supplement, compared to fiber, improved metabolic flexibility. Furthermore, urinary nitrogen excretion, and thereby protein oxidation, did not change after fiber supplementation, while protein oxidation increased in the placebo group (*p* = 0.048, Table 3).

**Table 3.** Changes in substrate metabolism and metabolic flexibility as measured by indirect calorimetry during the two-step hyperinsulinemic-euglycemic clamp after 12 weeks of fiber or placebo supplementation, combined with a high-protein diet.

		Placebo (*n* = 19)		Fiber (*n* = 18)		*p*-value		
	Step	Pre	Post	Pre	Post	Group	Time	Group * Time
*TEE (KJ/min)*	0 mU	5.23 ± 1.27^#^	5.12 ± 1.03^#^	4.96 ± 0.63	4.90 ± 0.61	0.490	0.202	0.671
	10 mU	5.26 ± 1.13	4.99 ± 1.04	4.87 ± 0.65	4.86 ± 0.5	0.276	0.017*	0.022*
	40 mU	5.29 ± 1.04	5.31 ± 1.02	5.06 ± 0.72	4.87 ± 0.5	0.081	0.208	0.345
*RQ*	0 mU	0.77 ± 0.04^#^	0.78 ± 0.03^#^	0.78 ± 0.04	0.77 ± 0.03	0.994	0.490	0.206
	10 mU	0.80 ± 0.03	0.81 ± 0.04	0.81 ± 0.03	0.80 ± 0.04	0.682	0.463	0.111
	40 mU	0.86 ± 0.03	0.89 ± 0.05	0.88 ± 0.04	0.88 ± 0.05	0.637	0.120	0.010*
*∆ RQ*	0–10 mU	0.03 ± 0.03	0.03 ± 0.04	0.03 ± 0.04	0.03 ± 0.03	0.715	0.910	0.806
	0–40 mU	0.10 ± 0.04	0.11 ± 0.05	0.11 ± 0.05	0.11 ± 0.05	0.776	0.458	0.206
	10–40 mU	0.06 ± 0.04	0.08 ± 0.03	0.08 ± 0.02	0.08 ± 0.03	0.973	0.234	0.120
*CHO oxidation (g/min)*	0 mU	0.048 ± 0.054^#^	0.061 ± 0.045^#^	0.057 ± 0.039	0.054 ± 0.038	0.918	0.711	0.365
	10 mU	0.081 ± 0.036	0.085 ± 0.044	0.088 ± 0.031	0.086 ± 0.047	0.822	0.865	0.368
	40 mU	0.159 ± 0.042	0.185 ± 0.044	0.178 ± 0.047	0.168 ± 0.065	0.951	0.527	0.027*
*Fat oxidation (g/min)*	0 mU	0.088 ± 0.031^#^	0.076 ± 0.023^#^	0.078 ± 0.021	0.077 ± 0.015	0.900	0.080	0.111
	10 mU	0.076 ± 0.028	0.063 ± 0.028	0.063 ± 0.017	0.064 ± 0.019	0.487	0.040*	0.012*
	40 mU	0.046 ± 0.02	0.032 ± 0.027	0.033 ± 0.017	0.032 ± 0.024	0.349	0.019*	0.006*
*Protein oxidation (g/min)*	0 mU	0.051 ± 0.012^#^	0.061 ± 0.013^#^	0.052 ± 0.018	0.052 ± 0.018	0.468	0.014*	0.048*
*Total urinary nitrogen (g/24 h)*	0 mU	11.835 ± 2.816^#^	14.104 ± 2.962^#^	11.962 ± 4.199	11.937 ± 4.107	0.468	0.014*	0.048*

Data are presented as mean ± SD. *p*-values are corrected for age, sex, BMI, and fat-free mass. CHO = carbohydrate, RQ = respiratory quotient, TEE = total energy expenditure. ^#^*n *= 20, *p-value < 0.05.

### Satiety hormones

Both fasting and insulin-stimulated plasma levels of glucagon-like peptide 1 (GLP-1) and peptide YY (PYY) were comparable between groups at baseline and over time (Table 2).

### Inflammatory markers

Circulating inflammatory markers tumor necrosis factor-*α* (TNF-*α*), interferon-*γ* (IFN-*γ*), interleukin-6 (IL-6), IL-8, and IL-10, and C-reactive protein (CRP) were not different between the groups or over time, except for significant opposite responses between the groups in IL-6 concentrations over time, increasing in the fiber and decreasing in the placebo group (*p* = 0.025, Table 2). Additionally, leukocyte counts tended to follow a similar pattern (*p* = 0.066).

### Gut permeability

Liposaccharide-binding protein (LBP) was not altered over time in any of the groups but was lower in the fiber group throughout the entire intervention (*p* = 0.007, Supplementary Figure 2A, Supplementary Table 5).

The multisugar test showed no changes in proximal gut permeability (*p* = 0.658, Supplementary Figure 2B, Supplementary Table 5), while distal intestinal permeability significantly increased in the fiber group compared to placebo (*p* = 0.046, Supplementary Figure 2C, Supplementary Table 5).

### Microbial metabolites

#### Fecal metabolites

No differential changes occurred between the groups after 12 weeks of intervention in any of the fecal metabolite concentrations (Table 4). Even though the fiber group showed higher overall concentrations of total fecal BCFA, isobutyric acid (isoBA), and propionic acid (PA) compared to placebo, no differential effects were observed over time. Fiber supplementation tended to increase relative acetic acid (AA) concentrations over time, compared to a slight decrease in the placebo group (*p* = 0.073), with an opposite trend in relative hexanoic acid (HA) abundance (*p* = 0.092).

**Table 4.** Changes in absolute and relative concentrations of fecal and plasma short-chain fatty acids (SCFA) and branched-chain fatty acids (BCFA) between 0, 6, and 12 weeks of fiber or placebo supplementation, combined with a high-protein diet.

	Placebo (*n* = 21)			Fiber (*n* = 19)			*p*-value		
FECAL	Pre	Week 6	Post	Pre	Week 6	Post^#^	Group	Time	Group * Time
*Total SCFA (μmol/g)*	28.45 ± 15.32	29.67 ± 23.07	26.47 ± 19.01	33.08 ± 16.62	40.43 ± 23.01	35.85 ± 20.63	0.083	0.360	0.651
*Total BCFA (μmol/g)*	1.14 ± 0.30	0.99 ± 0.35	1.26 ± 0.79	1.27 ± 0.39	1.31 ± 0.51	1.83 ± 1.65	0.045*	0.135	0.305
*SCFA:BCFA*	24.42 ± 11.23	28.68 ± 22.91	22.15 ± 14.36	26.23 ± 12.70	39.12 ± 23.08	24.89 ± 17.01	0.093	0.331	0.668
*AA (μmol/g)*	15.79 ± 10.18	16.63 ± 15.25	13.91 ± 11.45	17.22 ± 9.25	22.33 ± 14.12	19.35 ± 12.10	0.162	0.287	0.589
*PA (μmol/g)*	6.00 ± 2.62	6.43 ± 3.77	6.44 ± 4.61	7.70 ± 4.23	9.21 ± 5.73	8.11 ± 4.34	0.044*	0.320	0.653
*BA (μmol/g)*	4.65 ± 2.64	4.71 ± 4.06	4.26 ± 3.10	5.96 ± 3.69	6.84 ± 4.07	5.97 ± 4.31	0.054	0.564	0.784
*VA (μmol/g)*	1.15 ± 0.41	1.09 ± 0.57	1.03 ± 0.49	1.32 ± 0.51	1.25 ± 0.40	1.39 ± 0.77	0.090	0.587	0.649
*HA (μmol/g)*	0.85 ± 0.28	0.81 ± 0.30	0.83 ± 0.18	0.87 ± 0.25	0.80 ± 0.30	1.03 ± 0.62	0.336	0.259	0.457
*isoBA (μmol/g)*	0.54 ± 0.16	0.47 ± 0.19	0.51 ± 0.31	0.60 ± 0.21	0.62 ± 0.22	0.90 ± 0.86	0.022*	0.098	0.233
*isoVA (μmol/g)*	0.59 ± 0.15	0.52 ± 0.18	0.75 ± 0.75	0.67 ± 0.20	0.69 ± 0.31	0.93 ± 0.80	0.131	0.219	0.459
*AA (% of SCFA)*	52.24 ± 7.79	49.16 ± 12.12	48.59 ± 9.44	50.52 ± 6.09	53.23 ± 6.09	53.18 ± 6.71	0.310	0.917	0.073
*PA (% of SCFA)*	23.39 ± 6.05	26.10 ± 7.84	25.84 ± 6.08	23.90 ± 4.47	23.07 ± 4.47	23.70 ± 5.56	0.305	0.403	0.123
*BA (% of SCFA)*	15.82 ± 3.60	15.15 ± 4.32	15.77 ± 3.94	17.16 ± 4.23	16.87 ± 3.23	15.46 ± 4.06	0.282	0.543	0.449
*VA (% of SCFA)*	4.73 ± 1.72	5.04 ± 2.67	4.97 ± 2.32	4.79 ± 1.97	3.92 ± 1.83	4.31 ± 1.58	0.337	0.664	0.184
*HA (% of SCFA)*	3.83 ± 2.02	4.54 ± 3.35	4.83 ± 2.88	3.64 ± 2.56	2.91 ± 2.07	3.35 ± 1.82	0.135	0.510	0.092
*isoBA (% of BCFA)*	47.38 ± 4.55	44.87 ± 4.50	43.99 ± 11.07	46.80 ± 5.23	51.92 ± 13.62	47.90 ± 3.99	0.215	0.594	0.355
*isoVA (% of BCFA)*	52.62 ± 4.55	55.13 ± 4.50	56.01 ± 11.07	53.20 ± 5.23	48.08 ± 13.62	52.10 ± 3.99	0.215	0.594	0.355
**PLASMA**									
*Total SCFA (μmol/l)*	41.97 ± 19.50	-	50.47 ± 33.76	70.25 ± 37.79	-	72.15 ± 49.50	0.009*	0.319	0.526
*Total BCFA (μmol/l)*	2.87 ± 1.62	-	3.58 ± 2.18	3.65 ± 1.89	-	3.92 ± 2.10	0.359	0.120	0.489
*AA (μmol/l)*	39.29 ± 18.87	-	47.70 ± 33.41	66.62 ± 36.86	-	68.77 ± 9.56	0.011*	0.301	0.538
*PA (μmol/l)*	1.85 ± 0.94	-	1.92 ± 0.67	2.20 ± 0.96	-	2.27 ± 0.79	0.152	0.574	0.992
*BA (μmol/l)*	0.46 ± 0.45	-	0.47 ± 0.45	0.47 ± 0.39	-	0.53 ± 0.49	0.725	0.565	0.679
*VA (μmol/l)*	0.03 ± 0.05	-	0.02 ± 0.04	0.07 ± 0.15	-	0.04 ± 0.04	0.321	0.073	0.590
*HA (μmol/l)*	0.33 ± 0.38	-	0.36 ± 0.29	0.88 ± 1.97	-	0.56 ± 0.40	0.128	0.502	0.405
*LA (μmol/l)*	629.75 ± 252.07	-	617.40 ± 233.56	578.05 ± 251.58	-	551.82 ± 178.06	0.328	0.603	0.851
*isoBA (μmol/l)*	0.83 ± 0.63	-	0.98 ± 0.68	0.99 ± 0.59	-	1.11 ± 0.69	0.475	0.191	0.881
*isoVA (μmol/l)*	0.55 ± 0.34	-	0.76 ± 0.55	0.87 ± 0.39	-	0.84 ± 0.52	0.147	0.206	0.104
*2-MBA (μmol/l)*	1.49 ± 0.71	-	1.84 ± 1.03	1.78 ± 1.01	-	1.97 ± 0.95	0.478	0.082	0.573

AA = acetic acid, BA = butyric acid, HA = hexanoic acid, isoBA = isobutyric acid, isoVA = isovaleric acid, PA = propionic acid, VA = valeric acid, 2-MBA = 2-methylbutyric acid. *p-value <0.05, ^#^*n* = 18.

#### Circulating metabolites

Overall plasma concentrations of total SCFA and AA were significantly higher in the fiber group compared to placebo (*p* = 0.009 and *p* = 0.011, respectively; Table 4). After the 12-week intervention, no differential effects were observed in any of the circulating SCFA and BCFA. As week 6 data were derived from venous blood samples, results are not readily comparable to the data from CID2 and 4, during which arterialized samples were obtained. Nevertheless, we were able to compare the patterns of changes in the first 6 weeks between the groups (Supplementary Table 6). All BCFA showed differential trends in the first 6 weeks, remaining relatively stable or decreasing in the fiber group, while they increased in the placebo group (*p* = 0.040, Supplementary Figure 3). Including all timepoints in our analyzes, however, eliminated these effects, except for a persistent increase in isovaleric acid (isoVA) in the placebo compared to the fiber group (*p* = 0.009). Overall, it appears that fiber supplementation, compared to placebo, did not alter long-term circulating SCFA and BCFA concentrations, despite preventing a transient increase in BCFA.

### Fecal microbiota composition

**Figure 3.**
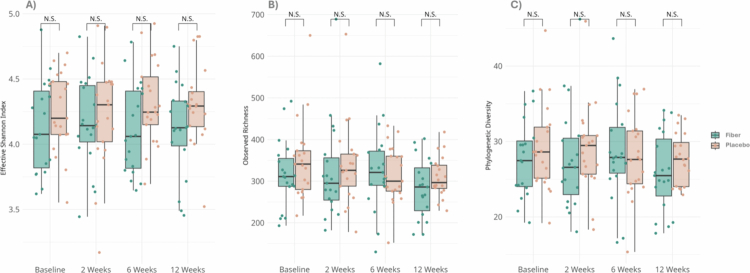
Changes in alpha-diversity on ASV level, measured by A. Effective Shannon index, B. observed richness, and C. Faith's phylogenetic diversity between the groups over time at weeks 0, 2, 6, and 12 of fiber versus placebo supplementation combined with a high-protein diet. N.S. = non-significant difference (*p* > 0.05).

Microbial composition was measured using 16S rRNA gene amplicon sequencing at baseline and after 2, 6, and 12 weeks. Alpha-diversity on ASV (Figure 3) and genus level was similar between groups at all timepoints and did not change over time. Similarly, no differences in beta-diversity between the groups over time were observed. Accordingly, the microbial composition was comparable between the groups at all timepoints during the intervention, both at ASV and genus level, compared using Principal Component Analysis (PCA, Supplementary Figure 4) and principal coordinate analysis (PCoA) plots and permutational multivariate analysis of variance (PERMANOVA), both at the ASV and genus levels (Supplementary Figure 5). The relative abundance of the 15 most abundant taxa at baseline and week 12 are depicted in Supplementary Figure 6. Additional analyzes on changes in relative abundances of individual taxa showed no significant differences between the groups over time (Supplementary Figure 7). Altogether, 12 weeks of fiber supplementation combined with a plant-focused high-protein diet did not affect the fecal microbial composition.

### Well-being

General well-being, measured by the RAND-36 and perceived stress scale (PSS) questionnaires at baseline and after the intervention, was not different between groups (Supplementary Table 7). However, we found a significant overall time effect in the mental component score, driven by significant changes in each group at different time points, but with a limited effect size, suggesting variability in the self-reported data without clinical relevance. Perceived stress was not different between groups over time (Supplementary Table 7).

### Gastrointestinal symptoms

Stool consistency, based on Bristol stool chart data at weeks 0, 2, 6, and 12, was not different between the groups or over time (*p* = 0.279, Supplementary Table 8). Additionally, no significant changes in gastrointestinal symptoms as assessed by the gastrointestinal symptom rating scale (GSRS) occurred after fiber supplementation compared to placebo. Overall, however, participants in the fiber group more often expressed symptoms of bloating, flatulence, and the feeling of incomplete fecal voiding compared to placebo, with the latter symptom already being elevated at baseline (Supplementary Table 9). Both groups experienced increased complaints of diarrhea (*p* = 0.019) and painful hunger (*p* = 0.001) over time. Urgency tended to increase in both groups after 12 weeks, but tended to be more pronounced in the fiber group compared to placebo (*p* = 0.092).

### Eating behavior

Twelve weeks of intervention did not result in differential changes in any of the three domains of the three-factor eating questionnaire (TFEQ) between the groups (cognitive restraint of eating, disinhibition, and feeling of hunger; Supplementary Table 10). However, the participants in the fiber group reported significantly higher feelings of hunger before and after the intervention compared to placebo (*p* = 0.024). Moreover, both the fiber and the placebo group reported a lower feeling of hunger after the 12-week intervention (*p* = 0.001).

## Discussion

The DISTAL-study, a 12-week randomized, placebo-controlled trial, was the first study to investigate the effects of potato fiber and sugar beet pectin supplementation within the context of a high-protein (±45% plant-based protein) diet on peripheral insulin sensitivity (IS) in individuals with overweight/obesity and at increased risk of developing T2D. Furthermore, we assessed tissue-specific insulin sensitivity, microbial composition and functionality, substrate metabolism, and circulating cardiometabolic and inflammatory markers. Prior *in vitro* fermentation experiments^20^ indicated that the investigated fiber mixture would steer towards saccharolytic fermentation in the distal colon, increasing distal saccharolytic metabolite production, including SCFA. Subsequently, this shift in microbial metabolite profile could, potentially, improve metabolic health and insulin sensitivity.^1^^,^^3^^,^^4^^,^^8^^,^^20^ Nevertheless, fiber supplementation in the current study tended to reduce glucose disposal per unit of insulin (Rd/I) compared to a high-protein diet alone. Additionally, fiber supplementation, compared to placebo, lowered insulin-stimulated carbohydrate oxidation and reduced the suppression of fat oxidation. Lastly, distal gut barrier permeability increased after fiber intake, while proximal gut barrier integrity remained unaltered. Our data suggest that adding a potato fiber and sugar beet pectin mixture to a high-protein, partially plant-based diet worsened, rather than improved, cardiometabolic health in individuals with overweight/obesity and at risk of developing T2D. Surprisingly, we found no differences in microbial composition and functionality between the groups over time. Overall, these results are in contrast to our hypothesis and studies that were successful in increasing saccharolytic fermentation throughout the colon by dietary fibers or supplements.^1^^,^^8^^,^^10^^,^^26-28^

Epidemiological evidence indicates that dietary fibers positively influence host metabolism, especially when increased within a whole-food approach,^6^^,^^8^^,^^26^^,^^29^ yet isolated fiber sources have limited and controversial effects on the microbiota and host metabolism.^30^ Furthermore, fibers may reduce protein fermentation by providing an alternative energy source for gut microbes, allowing proteins to be utilized primarily for microbial biomass rather than energy production.^31^ Hence, increasing colonic protein load by increasing dietary proteins, and in particular less digestible plant-based proteins, allowed for a more robust evaluation of the capacity of a distally fermenting fiber mixture to suppress protein fermentation. However, besides a transient fiber-induced reduction in circulating BCFA after 6 weeks, the intended shift in microbial composition, as well as saccharolytic and proteolytic fermentation, evidenced by fecal and plasma SCFA, was not achieved. As aforementioned, we specifically selected this fiber combination for its ability to induce distal SCFA production in the fecal microbiota of individuals with impaired glucose metabolism in an *in vitro* model of the human colon.^20^ Despite this evidence-based, stepwise approach, we failed to achieve increased SCFA production, which might be attributed to the complexity of digestive processes *in vivo*, particularly when ingesting fibers within a whole-food, high-protein context.^9^^,^^20^^,^^32^ The lack of changes in both composition and functionality of the gut microbiome may likely explain the absence of observed metabolic health benefits after the fiber mixture supplementation in this study.

Previous studies have shown that plant-based, compared to animal-derived, proteins can exert cardiometabolic benefits, including improved postprandial glucose and insulin responses, as well as reduced inflammation, independent of changes in microbial composition.^16^^,^^17^^,^^33^ Consistently, we showed improved IS in the plant-protein-rich diet alone (placebo), which coincided with a shift towards increased insulin-stimulated carbohydrate oxidation and reduced fat oxidation, suggesting improved metabolic flexibility. Interestingly, fiber supplementation mitigated the increase in urinary nitrogen excretion, which is reflective of increased protein oxidation, that was observed in the placebo group. Therefore, the complex fibers in this study may have counteracted some of the beneficial effects of the high-protein diet. Based on these results, it can be speculated that the fiber mixture reduced protein digestion and absorption, limiting amino acid availability and oxidation, which is supported by literature showing lower urinary nitrogen and increased fecal nitrogen excretion after increasing fiber intake in animals and humans.^34-37^ In case fiber supplementation indeed hinders protein digestion and/or absorption, larger amounts of proteins might reach the colon, potentially resulting in increased proteolytic fermentation, which in turn may neutralize the beneficial effects of saccharolytic fermentation induced by the fiber mixture. Furthermore, this might support the reduction in distal gut barrier integrity in the fiber group, which contradicts literature showing mainly positive effects of fiber intake on gut barrier function.^38^^,^^39^ However, since we found no changes in microbial composition, nor in measured markers of saccharolytic and proteolytic fermentation, a more complex interplay between dietary fibers and (plant-based) proteins may be at play.

Other factors potentially explaining our results include the phenotype of the study population, as well as the duration of the intervention. Abundant evidence has highlighted that the microbiome differs between individuals with normal weight and those with overweight/obesity or T2D.^11^^,^^12^ We previously showed that 1-d supplementation of a rapidly and slowly fermentable fiber combination increased distal colonic fermentation and metabolic health (*in vitro* and *in vivo*) in individuals with a healthy body weight but not in individuals with overweight and prediabetes.^10^ Additionally, it has been suggested that the microbiome of individuals with overweight may exhibit resistance to microbial alterations, and it may therefore be possible that 12 weeks of intervention *in vivo* was not sufficient to induce the expected microbial (and subsequent metabolic) alterations in our specific subpopulation.^5^^,^^40-42^ More thorough approaches for changing fermentation processes through fiber consumption in individuals with overweight or obesity, such as a broader variety of dietary fibers, as Zhao et al. have shown in individuals with T2D,^8^ or a longer intervention period, may be required.^43^ Moreover, besides studies using slowly fermentable intrinsic fiber mixtures that increased saccharolytic fermentation throughout the entire colon,^28^^,^^44^ promising results from recent advances in optimizing the gut microbiome have been proposed. A study using fecal microbial transplant (FMT) from healthy donors, for example, has shown that introducing a healthy microbiome can alleviate insulin resistance in individuals with disturbed glucose metabolism, both by itself and in combination with antidiabetic drugs or prebiotics.^45-47^ Similarly, a new dietary strategy targeted to restore a healthy microbial composition using a probiotic combined with minimally processed, primarily plant-based, and fiber-rich products in individuals living in industrialized societies was able to change the gut microbiome as well as improve host metabolic health.^48^ While practicality, feasibility, and efficacy should be optimized,^45^^,^^49^ future studies might be able to provide additional therapeutic options using FMT or other microbiome-altering diets and interventions, such as pre-, pro-, and antibiotic agents specifically favoring a beneficial microbial composition.^50-52^

In light of the unexpected results of the current study, further consideration should be given to the amount and type of fibers employed in the current study. Sugar beet pectin, a soluble fiber, is generally fermented rather rapidly in the proximal colon, whereas potato fiber, an insoluble fiber, is usually fermented to a lesser extent and more distally. Soluble fibers generally produce more SCFA,^29^^,^^53^^,^^54^ whereas insoluble fibers aid peristalsis and gut barrier function,^29^^,^^55^ amongst others. However, in the current study, we did not observe a synergistic effect of combining both types of fibers. Higher doses of potato fiber and pectin isolates may have yielded different results, considering that other studies showing beneficial results of these fibers generally supplied higher amounts.^27^^,^^54^^,^^56-58^ Nevertheless, consistent with our results, 4-week 15 g/d of the same pectin supplementation did not change microbial composition or functionality in young and older adults.^59^

The DISTAL-study is, to our knowledge, the first study to investigate the effects of an *in vitro-*optimized, slowly fermentable fiber mixture within the context of a high-protein, partially plant-based diet, hypothesized to increase distal microbial saccharolytic fermentation in individuals with overweight and insulin resistance. Through the high variety of established experiments within our state-of-the-art testing facilities, we were able to provide novel insights on the effects of co-administering fibers and proteins on human metabolism, the microbiome, gut health, tolerability, and general well-being. Naturally, the current study encountered some limitations. Although unavoidable when using a placebo in a nutritional trial, the placebo used in this study, maltodextrin, might affect host metabolism and the gut microbiome.^60-62^ Nevertheless, most of these effects seem to be rather negligible,^63^ especially given the low dose that was used in the current study, and unlikely affected the outcomes of our study. Even though the two-step hyperinsulinemic-euglycemic clamp is the gold standard to measure tissue-specific insulin resistance,^64^^,^^65^ it does not allow for evaluation of other biological processes, such as the rate of glucose or nutrient absorption, as well as related incretin responses, which require oral glucose tolerance or high-fat mixed-meal tests. Furthermore, since we investigated the effect of the fiber mixture within the context of a high-protein diet, we cannot disentangle whether our results were caused by the fiber mixture itself or because it prevented some of the effects of the plant-focused high-protein diet or a combination of both. Additionally, we only measured SCFA and BCFA as markers of microbial functionality. While these are established markers to estimate fermentation processes, other products of saccharolytic and proteolytic fermentation were not measured and may provide a more comprehensive overview of alterations in microbial metabolite repertoire. Moreover, the microbial composition we presented is derived from fecal samples and only describes relative microbial abundances, which do not fully resemble the composition of the entire colon, indicating that potential changes in proximal parts of the colon, as well as up- or downregulation of specific (sub)species, might remain unnoticed. Absolute abundances or higher-resolution measurements might therefore have been able to capture currently undiscovered changes. By chance, we found higher plasma SCFA, lower LBP concentrations, and a tendency towards higher fecal SCFA in the fiber group at baseline, without any other baseline differences in metabolic phenotype, microbial composition, or dietary (fiber) intake. While this might suggest that the placebo group has a slightly different microbial functionality at baseline, there were no differences in initial microbiota composition between the groups. Furthermore, these baseline differences would not explain the minor deterioration in insulin sensitivity and gut permeability in the fiber group, and we therefore do not expect that these baseline differences would have significantly impacted the current results. Overall, the present study employed rigorous methodologies and a comprehensive set of measurements, thereby enhancing its overall quality and reliability. As such, it is unlikely that changes to the study design would have considerably impacted our conclusions.

## Conclusion

Twelve weeks of daily supplementation with 15 g of potato fiber and sugar beet pectin, compared to an isocaloric placebo (maltodextrin), both within the context of a high-protein, partially plant-based diet, did not improve tissue-specific and whole-body insulin sensitivity, substrate metabolism, gut microbial functionality and composition, or cardiometabolic markers. In contrast, peripheral and whole-body insulin sensitivity, metabolic flexibility, and gut barrier function tended to decrease in the fiber group compared to an increase in the placebo group consuming only the high-protein diet. Altogether, this study provides novel insights into the cardiometabolic effects of dietary fibers, suggesting that combining a slowly and a more rapidly fermentable fiber may attenuate the systemic bioavailability, metabolism, and health benefits of (plant-derived) proteins, rather than enhance metabolic health via gut microbial modulation. Further studies are needed to unravel the impact of different fiber types and doses within the context of a high-protein, partially plant-based diet on cardiometabolic health and host–microbiota interactions, on a more in-depth, subspecies level and within a broader range of individuals.

## Supplementary Material

Supplementary material DISTAL_study_SUPPLEMENTARY_MATERIALS_v2_notrackchanges

Supplementary material CONSORT_2025_checklist_DISTAL

Supplementary material Study_protocol_DISTALstudy_MECapproved

## Data Availability

Deidentified data related to this study will be made available after publication. Anonymized 16S rRNA sequencing data will be made available through the European Nucleotide Archive after publication.

## Abbreviations

2-MBA: 2-methylbutyric acid
AA: Acetic acid
ASV: Amplicon sequence variant
AT: Adipose tissue
BA: Butyric acid
BCFA: Branched-chain fatty acids
BSS: Bristol Stool Scale
CHO: Carbohydrate
CLR: Centered log-ratio
CRP: C-reactive protein
DBP: Diastolic blood pressure
DEXA: Dual-energy X-ray absorptiometry
E%: Energy percentage
FDR: False discovery rate
FFA: Free fatty acid
GI: Gastrointestinal
GIR: Glucose infusion rate
GLP-1: Glucagon-like peptide 1
GSRS: Gastrointestinal symptom rating scale
HA: Hexanoic acid
HbA1c: Glycated hemoglobin A1
HDL: High-density lipoprotein
HOMA-IR: Homeostatic model assessment for insulin resistance
IFN-γ: Interferon-γ
IL: Interleukin
IS: Insulin sensitivity
isoBA: Isobutyric acid
isoVA: Isovaleric acid
LA: Lactic acid
LBP: Lipopolysaccharide-binding protein
LDL: Low-density lipoprotein
PA: Propionic acid
PCA: Principal component analysis
PCoA: Principal coordinate analysis
PSS-10: Perceived stress scale 10
PYY: Peptide YY
Ra: Rate of glucose appearance
Rd: Rate of glucose disposal
Rd/I: Rate of glucose disposal per unit of circulating insulin
RQ: Respiratory quotient
SBP: Systolic blood pressure
SCFA: Short-chain fatty acids
SQUASH: Short questionnaire to assess health-enhancing physical activity
T2D: Type 2 Diabetes
TEE: Total energy expenditure
TFEQ: Three-factor eating questionnaire
TNF-α: Tumor necrosis factor-α
VA: Valeric acid
