# Supplementary material for: Effects of a slowly fermentable fiber mixture against the background of a high-protein diet on insulin sensitivity and metabolic health in individuals with overweight: a randomized, placebo-controlled trial
Source: Gut Microbes. 2025 Dec 29;18(1):2606473. doi: 10.1080/19490976.2025.2606473 (PMC12758221; doi:10.1080/19490976.2025.2606473)
Supplement: Supplementary material — DISTAL_study_SUPPLEMENTARY_MATERIALS_v2_notrackchanges [file KGMI_A_2606473_SM6492.docx]

# SUPPLEMENTARY MATERIALS

## Materials and methods

The DISTAL study (“Using a complex carbohydrate mixture added to a high-protein **DI**et to **ST**eer fermentation and improve metabolic, gut and brain he**AL**th”) was a 12-week, parallel, double-blind, randomized, placebo-controlled dietary intervention trial. The study, performed between September 2022 and June 2024 at Maastricht University Medical Center+ (MUMC+, Maastricht, the Netherlands) and Maastricht University (Maastricht University, Maastricht, the Netherlands), was approved by the local Medical Ethics Committee (METC azM/UM, MUMC+, Maastricht, the Netherlands), registered at ClinicalTrials.gov (NCT05354245, https://clinicaltrials.gov/study/NCT05354245), and was carried out according to the principles of the Declaration of Helsinki (October 2013), and monitored by the Clinical Trial Center Maastricht (CTCM, Maastricht, the Netherlands). Patients and/or the public were not involved in the design, or conduct, or reporting or dissemination plans of this research.

The primary objective of this proof-of-principle study was to assess differences in peripheral IS after potato fiber and sugar beet pectin supplementation compared to an isocaloric placebo (maltodextrin), while both groups adhered to a high-protein diet. Secondary study outcomes included alterations in adipose and hepatic tissue IS, gut microbial composition and functionality, gut permeability, energy and substrate metabolism, circulating metabolites and cardiometabolic markers, neurocognitive functioning, perceived well-being, food reward-related brain activity, body composition, adipose and skeletal muscle tissue metabolism, and gastrointestinal side-effects of the intervention. The investigated fiber supplement has been found to increase SCFA production in the distal colon *in vitro.*^1^ The plant-focused, protein-rich diet was added to ensure sufficient protein supply to the colon. A detailed description of the measurements of which results are included in this paper can be found below.

## Study participants

We included males and females aged 30 to 75 years with a BMI between 28 and 40 kg/m^2^ and insulin resistance and/or impaired glucose metabolism. Participants were recruited via an existing volunteer database and online and paper media, including flyers, local newspaper advertisements, and online volunteer platforms. If a person expressed interest in participation, a phone call was conducted to explain the study design and in-/exclusion criteria, before planning a screening visit when no apparent exclusion criteria were present and the person remained interested. At the screening, after receiving extensive verbal and written information about the study, written consent was provided from all individuals, before eligibility was determined based on anthropometrics, blood pressure, and an interview to assess inclusion and exclusion criteria based on (medical) history. Additionally, a venous blood sample was drawn from an antecubital vein after a >10h overnight fast to determine insulin resistance and/or impaired glucose metabolism, which was defined by one or more of the following criteria: fasting plasma glucose of 5.6-6.9 mmol/l, HbA_1c_ between 42-47 mmol/mol (6.0-6.5%), and/or a Homeostatic Model Assessment of Insulin Resistance (HOMA-IR) value >1.85. Fasting glucose and HbA_1c_ ranges were derived from the definition of prediabetes by the American Diabetes Association and the World Health Organization, respectively.^2, 3^ HOMA-IR cut-off was based on the median of all screened participants (*n*>800) in a recent multicenter study in individuals with a BMI of 25-40 kg/m^2^ performed in the Netherlands.^4^ Additional analyses (hemoglobin, aspartate aminotransferase (AST), alanine aminotransferase (ALT), and creatinine) were performed to determine participant’s safety and well-being.

Individuals were excluded from participation if any of the following criteria were present: diagnosis of type 1 or 2 diabetes mellitus (DM), any cardiovascular disease (hypertension <160/100 mmHg was allowed), pulmonary, hepatic, kidney, auto-immune or gastrointestinal disease, a history of abdominal surgery (except appendectomy and cholecystectomy), major mental disorders preventing adequate participation (such as severe depression, psychosis, schizophrenia), malignancy (except non-invasive skin cancer), ongoing (infectious) diseases, diseases with a life expectancy ≤5 years, or any other diseases affecting glucose and/or lipid metabolism, pregnancy, substance abuse (nicotine use including e-cigarettes was allowed with a maximum of 20 cigarettes per day; alcohol was allowed with a maximum of >15 units/week; no drugs allowed), change in body weight ≥3kg over the last 3 months, planning to lose weight or follow a (hypocaloric) diet during the study period, regular strenuous physical activity (>3h per week), significant changes in physical activity or diet during study period, veganism or vegetarianism, presence of food allergies, intolerances or dietary restrictions interfering with the study design. Additionally, regular use of prebiotics, probiotics, and/or laxatives in the last 3 months before or during the study period was prohibited, as well as the use of antibiotics 3 months before the start of the intervention, medication that influences glucose or fat metabolism and/or inflammation (including β-blockers, chronic corticosteroid treatment), and any other medication known to interfere with study outcomes. Stable use (≥3 months before and during the study) of statins and antidepressants was allowed for logistical reasons. As Magnetic Resonance Imaging (MRI) measurements were part of the study design, additional exclusion criteria included: the presence of metal objects or MRI-incompatible implants in the body, tattoos or (visible) permanent make-up on the head/neck area, claustrophobia, and the diagnosis of epilepsy. Anticoagulant use, except acetylsalicylic acid or carbasalate calcium, was prohibited to allow safe biopsy. However, in case of interfering anticoagulant use, participants were included in the trial without tissue biopsies. A flowchart of participant recruitment can be found in Supplementary figure 1.

Individuals were randomly allocated to either receiving the fiber mixture or placebo using minimization (Qminim)^5^ with randomization factors of 1.0 for age, 0.8 for sex and BMI, and a base probability of 0.7 by means of biased coin.^6^ An independent researcher was responsible for the randomization and distribution of, to participants and investigators unrecognizable, dietary supplements to guarantee the double-blind design of the study.


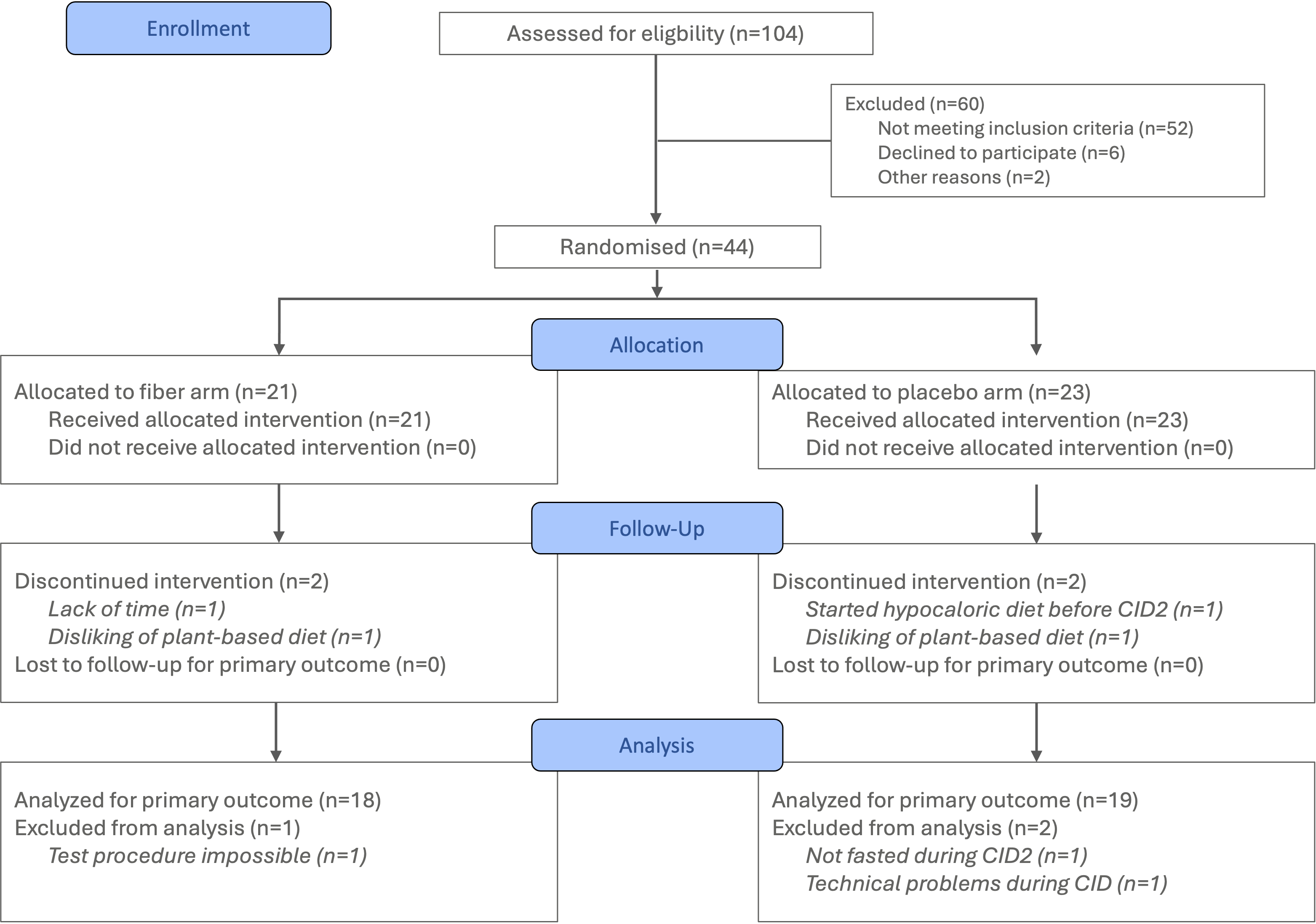


Supplementary Figure 1. CONSORT 2025 Flow Diagram^7^ of DISTAL study participant recruitment. CID = Clinical Investigation Day. See section “Data collection summary” for further details on participant data collection and analyses.

## Dietary intervention

### Investigational products

Before the start of the human trial, *in vitro* experiments were carried out to evaluate a diverse set of fibers and combinations for their potential to increase saccharolytic fermentation and decrease proteolytic fermentation in the distal colon. The design and results of this study are extensively described elsewhere,^1^ which demonstrated that the combination of potato fiber and sugar beet pectin led to the highest SCFA production and SCFA:BCFA-ratio in the distal colon, which was therefore used in the current human intervention.

The fiber supplement (15 grams/day) consisted of equal contribution of potato fiber (Paselli^TM^, Royal Avebe, Groningen, the Netherlands) and sugar beet pectin (GENU® BETA pectin, CP Kelco, Großenbrode, Germany). Maltodextrin (Glucidex IT 12, Roquette Freres, Lestrem, France), a fully digestible carbohydrate, was used as a placebo in an isocaloric manner. Both supplements were distributed in 3 equal weight sachets per day, one for each meal. Sachets were prefilled (Daklapack Filling, Lelystad, The Netherlands), containing exactly 5 grams of fiber mixture (1:1 ratio), or the isocaloric placebo, and labeled for each meal. Both products were similar in look, taste, and palatability. All products have been tested for safety and are approved for use in humans. Participants were instructed to use the supplements from the day after the pre-intervention clinical investigation days (CID1 and CID2) until the day before post-intervention measurements (CID3 and CID4) (**Figure 1**). All participants were asked to track their supplement intake and to return all provided sachets to determine adherence.

### High-protein diet

Participants in both arms were instructed to follow a high-protein diet during the entire intervention period, with 25 energy-% (E%) proteins, 30E% fats (<30% saturated fats), and 45E% carbohydrates, all in line with Dutch dietary guidelines.^8, 9^ The protein content was approximately 45% plant-based, and fiber consumption was aimed at approximately 30 grams/day (fiber supplement not included).

The diet was tailored to each participant, considering the participant’s calculated energy requirements based on the Harris-Benedict formula with an activity factor of 1.3 and personal food preferences, and was aimed at weight maintenance.^10^ Participants were provided with key food products and were reimbursed for required products that would normally not be part of their regular diet (e.g. plant-based meat replacements and dairy, legumes, peanut butter, unsalted nuts) to aid adherence. Additionally, within the prescribed diet, each participant was appointed a personalized daily amount of ‘points’ (9kcal/point, 5-10 points/day), which they could spend on products of their choice, based on a predetermined ‘price list’. All dietary prescriptions were provided by our in-house dietician. Moreover, participants were counseled by the same dietician or a member of the study team to assess adherence and ensure weight maintenance. In case of considerable weight changes (>2-3kg during the intervention period), alterations in energy intake were made to prevent further weight (e.g., adding or removing slices of bread per day, switching to more caloric-dense cheese/yogurt, etc), while maintaining the macronutrient ratio as previously described. Participants were instructed to adhere to the diet from the day after CID2 until the night before CID3 to limit any acute effects of the diet and supplement on insulin sensitivity measured at CID4.

## Study design

All participants underwent 4 CIDs, two before and two after the 12-week intervention, consisting of various clinical experiments (Figure 1). CIDs 1 and 2 were conducted before the start of the intervention for baseline characterization. These measurements were repeated after 12 weeks (CIDs 3 and 4). When possible, preferably 1 but no more than 7 days, were planned between CIDs 1 and 2, and between 3 and 4.

On CIDs 1 and 3, the multisugar test, dual-energy X-ray absorptiometry (DEXA) scan, CANTAB test, functional MRI (fMRI), the subcutaneous abdominal adipose tissue biopsy (ScAT), and a physical examination (anthropometrics, blood pressure) were performed. Additionally, the first dietary counseling session took place on CID1. On CIDs 2 and 4, the two-step hyperinsulinemic-euglycemic clamp, indirect calorimetry, and skeletal muscle biopsy were performed. The evening before CIDs 2 and 4, participants consumed a low-fiber standardized meal: “Aviko maaltijdpannetje malse kip” (Aviko, Royal Cosun, Breda, The Netherland) (385 kJ/92 kcal, 1.7g fat, 12 g carbohydrates, 5.4 g protein, 2.4 g fibers per 100 g; total 450g).

Participants returned for two on-site visits at weeks 2 and 6 and were either contacted by phone or received for an on-site visit at week 9 based on participant’s preference. During these visits, a physical examination was performed, and dietary intake was assessed and adjusted if needed. Additionally, fasted blood samples were taken in week 6 with a venipuncture from an antecubital vein.

Each visit, except for week 2 and 9 visits, participants visited our facilities after a >10h overnight fast, were asked to refrain from alcohol consumption and strenuous physical activity for >24h prior to each visit and were instructed to travel to our research facilities by car or public transport to limit potential disturbances by physical activity.

Participants were instructed to remain weight stable during the intervention period, refrain from using any laxatives, prebiotics, and probiotics, maintain regular physical activity levels, and contact the researchers in case of changes in medication use. Moreover, the occurrence of (serious) adverse events ((S)AEs) and the use of concurrent medication were monitored at each visit.

### Microbial composition and functionality

Before CIDs 1 and 3 and the visits in weeks 2 and 6, participants collected fecal samples at home using three tubes and a container (Fecotainer®, Excretas Medical, Enschede, The Netherlands) specifically designed for collection of fecal material to allow evaluation of microbial composition and functionality. After collection, taking place as close as possible to and not more than 2 days before the visit, samples were put in their home freezer at -18°C until brought to the university to be stored at -80°C until further analysis. Detailed descriptions of the conducted chemical analyses can be found later in this paper.

### Tissue-specific insulin sensitivity

The two-step hyperinsulinemic-euglycemic clamp was conducted to assess whole-body and tissue-specific insulin sensitivity, which is considered the gold standard.^11^ During each step, insulin was infused at either a low or a high rate to determine hepatic or muscle insulin sensitivity, respectively. Before the procedure, two intravenous cannulas were inserted, one in an antecubital vein infusion, and another on the dorsal side of the contralateral hand after being placed in a hotbox (60°C) during the entire procedure to ensure arterialized venous blood sampling.

After baseline fasted blood samples were taken, a [6,6-^2^H_2_]glucose tracer was infused at a constant rate 0.04mg/kg^-1^/min^-1^ during the entire experiment (*t*=-120 min) to allow calculation of endogenous glucose production (EGP), rate of glucose appearance (Ra) and glucose disposal (Rd). After two hours (*t*=0 min), insulin infusion (Novorapid, Novo Nordisk bv, Bagsværd, Denmark), was started at a rate of 10 mU/m^2^ body surface/min for 3 hours (low-step) for determination of hepatic glucose production. After 3 hours (*t*=180 min), insulin infusion was increased to 40 mU/m^2^/min, completely inhibiting EGP for another 2.5 hours, to allow determination of muscle insulin sensitivity (high-step). Starting at *t*=0, blood glucose levels were monitored every 5 minutes with a handheld glucose analyzer (Accu-Chek Inform II, Roche Diagnostics, Basel, Switzerland), to ensure euglycemia during the entire procedure. Glucose levels were maintained at 4.8-5.2 mmol/l, using a 20%-glucose solution (B. Braun Medical, Melsungen, Germany; Baxter International Inc, Deerfield, Illinois, USA) at variable rates.

Steady state calculations were conducted using data from the last 30 minutes of each step. The rates of glucose appearance (Ra), glucose disposal (Rd), glucose disposal per unit of circulating insulin (Rd/I), M-value were calculated according to previously described formulas,^11^ along with calculating the average glucose infusion rates (GIR) during each steady state. Insulin-mediated free fatty acid suppression was calculated at the end of the low-step as a measure of adipose tissue (AT) insulin sensitivity.

### Energy expenditure and substrate metabolism

Open-circuit indirect calorimetry was performed using a ventilated hood system (Omnical, Maastricht Instruments, Maastricht) to assess energy expenditure and substrate utilization during the clamp at rest (*t*=-30 until *t*=0), during the low (*t*=150 until *t*= 180), and high-step (*t*=300 until *t*=330). VCO_2_ (L/min) and VO_2_ (L/min) were measured every minute, of which the last 10 minutes were averaged. Fat, carbohydrate, and protein oxidation, as well as resting energy expenditure (EE) and the respiratory quotient (RQ) were calculated using Weir and Frayn equations,^12, 13^ with protein oxidation being based on urinary nitrogen excretion. Furthermore, metabolic flexibility was determined by calculating the delta RQ (ΔRQ) between baseline RQ and the RQ of the high-step RQ,^14^ but also by assessing the insulin-stimulated switch to carbohydrate oxidation and reduction in fat oxidation.

### Gut permeability and urinary nitrogen excretion

A multisugar test (MS-test) was used to assess gut permeability at CID1 and 3.^15^ Participants were instructed to use the bathroom to ensure complete urinary voiding before ingesting a mixture of sugars (1g lactulose (Eurogenics N.V., STADA Group, Brussel, Belgium), 1g sucralose (Harrison Sport Nutrition S.L., Albolote, Spain), 1g erythritol (Mattisson Healthcare, IJsselstein, The Netherlands), 0.5 L-rhamnose (Sigma Aldrich, Merck Life Sciences N.V., Amsterdam, The Netherlands) dissolved in 150ml water. Subjects were instructed to ingest the solution within 5 minutes and collect all urine for the subsequent 24 hours, enabling determination of urinary excretion of the ingested sugars. Lactulose/rhamnose (L/R) ratio in the 0-5h urine portion and sucralose/erythritol (S/E) ratio in the 5-24h urine portion were used to evaluate upper GI- and colonic permeability, respectively. Participants were instructed to remain fasted during the first 5 hours, and to refrain from alcohol or intake of any food products containing one of the ingested sugars during the following 24 hours, as well as to fully empty their bladders at the end of each collection period (after 5 and 24 hours).

Urine was collected in opaque containers provided by the researchers, containing antibiotics (Neomycin sulfate, Merck Life Science N.V., Amsterdam, The Netherlands) and boric acid (1g/unit, Medicago BA, Uppsala, Sweden) to prevent bacterial growth and allow metabolite fixation for future analyses, respectively. The total urine volume in each jar was measured twice and recorded, before collecting 0-5h, 5-24h and 0-24h samples, which were snap-frozen and stored at -80°C after collection. Samples of 0-24h were additionally used to determine urinary nitrogen concentrations, enabling 24h protein oxidation estimations.

### Anthropometrics and body composition

During all on-site visits, except CID2s and 4, blood pressure and anthropometrics were measured. Blood pressure was measured in triplicate by an automated sphygmomanometer (Omron Healthcare Europe B.V., Hoofddorp, the Netherlands). The initial measurement was excluded for acclimatization purposes; the last two were averaged when both measurements were within a similar range (<10mmHg difference). When the 10mmHg difference between the measurements was exceeded, an additional measurement took place before averaging the measurements closest together and within the same 10mmHg range. Weight, as well as waist and hip circumferences, were measured in underwear, according to the guidelines of the WHO^16^ in triplicate, and averaged afterward. Weight and height were recorded to the nearest 0.1 kg and 0.5 cm, respectively, whereas waist and hip circumferences were measured to the nearest mm. A DEXA scan was performed on CID1 and 3 to evaluate body composition (body fat percentage, body fat mass, abdominal and gynoid fat deposition, visceral adipose tissue, lean body mass), using the facilities at MUMC+ (Discovery A, Hologic).

### Self-reported data

During the intervention period, participants were asked to fill in multiple questionnaires electronically at home prior to each visit. All questionnaires were filled in before CID1 and CID3. In addition, 3-day dietary food records and self-reported physical activity (Short QUestionnaire to ASsess Health-enhancing physical activity, SQUASH)^17^ were also collected at week 6, and the Gastrointestinal Symptom Rating Scale (GSRS) and the Bristol Stool Scale (BSS) at weeks 2, 6, and 9. Additionally, questionnaires to assess general well-being (RAND-36),^18, 19^ perceived stress (Perceived Stress Scale-10 (PSS-10)),^20-22^ and eating behavior (Three-Factor Eating Questionnaire (TFEQ))^23^ were collected.

#### Dietary intake

To analyze baseline dietary intake and diet adherence, three 3-day food records were filled in over three random days (one weekend day and 2 weekdays). Participants could use the Dutch mobile phone application “Eetmeter” (Voedingscentrum, Den Haag, the Netherlands) or a standardized paper diary. When participants used a paper diary, researchers transferred the information to “Eetmeter” to ensure all food diaries used the validated Dutch food composition database (Nederlands Voedingsstoffenbestand 2016/5.0, National Institute for Public Health and Environment, Ministry of Health, Welfare and Sport, The Hague, The Netherlands) and to calculate the average macronutrient composition (protein, fat, and carbohydrate intake), energy intake, and fiber content (of the consumed diet, excluding the fiber supplement). To account for dietary misreporting, the ratio between reported energy intake (EI) and their basal metabolic rate (BMR) was calculated.^24, 25^ EI/BMR-ratios between 0.87 and 2.75 were included in the final analyses.

#### Physical activity

Habitual physical activity was assessed through the SQUASH questionnaire to assess changes in physical activity during the intervention. This questionnaire asks to recall the frequency, duration, and intensity of their physical activities over a typical week and includes a wide range of activities, including commuting, household activities, and sports.^17^

#### Gastrointestinal symptoms

Gastrointestinal side effects were assessed using the Dutch Gastrointestinal Symptom Rating Scale (GSRS), assessing both severity and frequency of symptoms over the last 7 days. The Bristol Stool Scale (BSS) evaluated changes in stool consistency, a potential indicator of gastrointestinal transit time. Participants were asked to rate their average stool consistency over the last 7 days ranging between types 1 to 7, indicating severe constipation and severe diarrhea, respectively. Furthermore, a separate BSS-type for each collected fecal sample was registered to determine the consistency of that specific collected sample, to ensure quality of the microbial analyses.

#### General well-being and stress

The Dutch RAND 36-Item Short Form Health Survey (SF-36)^18, 19^ and the Perceived Stress Scale-10^20-22^ evaluated health-related quality of life (physical and mental health) and perception of stress, respectively. To enhance comprehensibility and translatability, RAND-36 scores were combined into a physical and mental components score (PCS and MCS, respectively).^26^

#### Eating behavior

The Three-Factor Eating Questionnaire (TFEQ)^23^ was used to evaluate cognitive and behavioral aspects of eating habits. The three primary dimensions of eating behavior include the cognitive restraint of eating, disinhibition and hunger, and emotional eating.

### Microbial and biochemical analyses

#### Microbiota and fecal SCFA analysis

Fecal microbiota composition was determined in our laboratory (Campus Venlo, Maastricht University, The Netherlands) as described previously.^27, 28^ In brief, microbial DNA was extracted using the QIAamp Fast DNA Stool Mini Kits (Qiagen Benelux, Venlo, the Netherlands), following the manufacturer's guidelines. For sequencing, the V3-V4 region of the 16S rRNA gene was targeted using a 2-step PCR process to generate amplicons. Initially, 7.5 ng genomic DNA was amplified with primers 341F (5’-CCTACGGGNGGCWGCAG-3’) and 785R (5’-GACTACHVGGGTATCTAATCC-3’) with a total of 50 μl volume per PCR reaction. which included Illumina adapter sequences (sample-specific barcoded primers). For the second PCR, Illumina adapter sequences (sample-specific barcoded primers) were added. After each PCR step the amplicons were purified (QIAquick PCR Purification Kit) followed by amplicon size and quality assessment with Bioanalyzer (Agilent, Santa Clara, CA, USA). Purified PCR products were quantified by fluorometric analysis (Qubit™ dsDNA HS Assay Kit) and combined equimolarly to create a library.

For sequencing, the amplicons were multiplexed and sequenced on an Illumina MiSeq (San Diego, CA, USA) and performed according to standard Illumina protocols (Illumina, Eindhoven, the Netherlands). The raw sequence data were processed using the Illumina CASAVA pipeline (v1.8.3) and sequences were converted to FASTQ files using the BCL2FASTQ pipeline version 1.8.3. The quality threshold was determined according to the Phred quality score.

Forward and reverse primers were removed using Cutadapt v4.7.^29^ Sequences were processed using DADA2 v1.28.0 (Callahan et al., 2016): filtering (maxEE=2, truncLen=240/210 bp), denoising, merging (minOverlap = 10, maxMismatch = 0), and chimera removal (method='consensus'). ASVs with lengths <350 bp or >500 bp were discarded. A phylogenetic tree was built using FastTree v2.1.11^30^ under the GTR model. Taxonomy was assigned using the naïve Bayesian classifier and the SILVA v138.1 reference database.^31^

Fecal SCFA (acetate, propionate, butyrate, valerate, and caproate) and BCFA (isobutyrate and isovalerate) were measured as described previously^28^ by gas-chromatography mass-spectrometry (GC-MS, 8890 GC System; Agilent Technologies, Amstelveen, the Netherlands) at the same laboratory as the compositional analyses. In short, 0.5 gram of fecal material was combined with PBS (1:1) and homogenized for 2 minutes, then centrifuged (10 min, 14.000 g, RT), and, thereafter, 150 μl of the supernatant was extracted. An internal standard solution (containing methanol and the internal standard 2-ethyl butyric acid) and 20% formic acid with a total volume of 550 μl was added to the supernatant. Subsequently, the GC-MS injected 1 μl of the sample on a DB-FATWAX Ultra Inert column (30 m, 0.25 mm, 0.25 μm, Agilent) at a flow rate of 1.2 mL/min with the help of a PAL3 RSI 85 autosampler (Agilent). Calibration curves were used to derive SCFA concentrations by MS Quantitative Analysis (Quant-My-Way) software (Agilent).

### Blood sample collection and plasma, serum, and urine analysis

A wide range of metabolites, proteins, and hormones were measured during the present study. Arterialized blood samples were collected at baseline (fasted, t=-120) and multiple intervals during the clamp on CID2 and 4 and once a week 6 in pre-chilled ethylenediaminetetraacetic acid (EDTA), heparin, and K3-EDTA tubes, or in serum separator tubes (SST) tubes (Becton Dickinson, Eysins, Switzerland) at room temperature, depending on the evaluated metabolite. Immediately after collection in pre-chilled tubes, the blood was centrifuged (3000 rpm, 10 min, 4°C) and plasma was aliquoted, snap-frozen in liquid nitrogen, and stored at -80°C until further analysis. Serum samples were left at room temperature for at least 20 minutes before centrifuging (3000 rpm, 10 min, 21°C), aliquoting, snap-freezing, and storing at -80°C until further analysis.

Plasma glucose, insulin, FFA, inflammatory markers, C-reactive protein (CRP), and SCFA were determined at our in-house laboratories. Plasma glucose, FFA, and CRP were analyzed by colorimetric analysis with a Cobas Pentra C400 using commercially available kits; ABX Pentra Glucose HK CP reagent (Horiba ABX Diagnostics, Montpellier, France), NEFA HR reagents (Wako chemicals, Neuss, Germany), and CRP Horiba reagent (Horiba ABX Diagnostics, Montpellier, France), respectively. Enzyme-linked immunosorbent assay (ELISA) was used for Human Insulin measurement (Crystal Chem (Europe), Zaandam, Netherlands)) and analysis of interferon-γ(IFN-γ), IL-6, IL-8, IL-10 and tumor necrosis factor-alpha (TNF-α) (V-PLEX Plus Proinflammatory Panel 1 Human Kit) (MesoScale Discovery, Gaithersburg, MD, USA). Urinary nitrogen concentrations were determined by a Rapid Max N exceed (type CN-O-Rapid) at our department’s laboratory.

Plasma samples were prepared for SCFA analysis according to the previously described derivatization and isolation procedure,^32^ with slight modifications.^33^ The detection limits for acetate, propionate, and butyrate were 2.5, 0.05, and 0.05 µmol/L, respectively. Quantification was achieved by integrating peak areas and plotting calibration curves using Thermo Scientific Xcalibur 4.4 software. Lipopolysaccharide-binding protein (LBP) was analyzed with ELISA (Greiner, Microlon).^34^ Biotinylated polyclonal rabbit anti-human LBP was bound to captured human LBP. Streptavidin-peroxidase conjugate (Zymed, Invitrogen) was bound to the biotinylated antibody and reacted with the substrate, Tetramethylbenzidine (TMB, Sigma). The enzyme reaction was stopped by the addition of 1M H_2_SO_4_. Spectrophotometry was performed at 450nm with a detection limit of 390 pg/ml.

Fasted samples of glycated hemoglobin A1 (HbA_1c_) and leucocytes were immediately analyzed after collection at CID2, week 6, and CID4 at the laboratories of MUMC+. HbA_1c_ was determined using a D-100 System (HLPC dedicated hemoglobin testing system, Bio-Rad Laboratories B.V., Veenendaal, the Netherlands), while leucocytes were counted on a hematology analyze (XN-9000, Sysmex Nederland B.V., Etten-Leur, the Netherlands). Serum samples were sent to the laboratory of Dr. Stein & Collegae, Maastricht (Humicon BV, Maastricht, The Netherlands), and analyzed for triacylglycerol (TAG), total cholesterol (TC), high-density lipoprotein cholesterol (HDL), and low-density lipoprotein cholesterol (LDL). LDL/HDL and cholesterol/HDL ratios were calculated. Plasma GLP-1 and PYY concentrations were determined at the Department of Biomedical Sciences (University of Copenhagen, Copenhagen, Denmark). Satiety hormone samples were collected during CID2 and 4 at *t*=-120 and *t*=330, and at week 6. GLP-1 was collected in K3EDTA tubes, while PYY was collected in K2EDTA tubes, both enriched with dipeptidyl peptidase-IV inhibitor (Millipore, Darmstadt, Germany) at CID2 and 4. Total GLP-1 immunoreactivity was measured using an antiserum that equally detects both intact GLP-1 and its primary N-terminally truncated metabolite, as previously reported,^35^ whereas total PYY was analyzed with a radioimmunoassay measuring Human PYY (3-36) and PYY (1-36) equally well described elsewhere.^36^

### Statistical analysis

A power calculation was performed to determine the required sample size. 22 individuals were needed per group to allow detection of a physiologically relevant difference of 20% (2.86μmol/kg/min) in insulin sensitivity, assuming a power of 80%, an α-value of 5%, a within-group residual SD of 2.70, and a 20% dropout rate.

Since group comparability was assumed due to the randomized setup, baseline differences between the groups were not tested. Continuous data were analyzed using linear mixed models with repeated measures to assess differential effects between both groups over time, using intervention as a fixed factor, participant IDs as a random factor, and time for repeated measures. All data were compared between weeks 0 and 12, with data from weeks 2 and 6 included where possible and deemed necessary. For explorative reasons, acute effects between weeks 0 and 6 were analyzed were possible. Categorical data from questionnaires were analyzed with generalized estimated equations. Relevant assumptions of the models were checked by computing and visualizing the residuals and checked for normality and homogeneity of variance. In the absence of normality, ln-transformations were performed. Displayed data are presented as means with standard deviations or 95% confidence intervals. Presented *p*-values are two-tailed and adjusted for age, sex, and BMI at baseline unless specified otherwise. *P*-values below 0.05 are considered significant, and between 0.05 and 0.10 considered tendencies. All data were analyzed using SPSS Statistics software version 29 (Chicago, IL, USA), except for microbiota analyses. All visual graphs were made using GraphPad Prism (version 10.4 for Windows, GraphPad Software, Boston, Massachusetts USA). Microbiota analyses were carried out in R Statistical Software (v4.4.2; R core Team 2021). Alpha and beta diversity analyses were performed on unfiltered data using the effective Shannon Index, Faith’s phylogenetic diversity, the Aitchison distance, and the Generalized UniFrac distance. Differential abundance analyses were conducted on core microbiota (genera with a relative abundance of at least 0.1% in at least 10% of the total fecal samples) complemented with the 14 bacterial genera altered by the fiber mixture or high-protein background in our previously performed *in vitro* study.^1^ The centered log ratio (CLR) transformation was applied to genus abundances prior to differential abundance analyses. The Benjamini-Hochberg procedure was applied to correct for multiple testing and control the false discovery rate (FDR).^37^

## Data collection summary

After randomization, 4 individuals withdrew from the intervention prematurely for different reasons: no time for CIDs (*n*=1), not willing to follow plant-based diet (*n*=2), started hypocaloric diet after screening (*n*=1) (Supplementary Figure 1). The clamp could not be performed in 1 participant due to failure of intravenous cannula placements. SCAT biopsy was not conducted in 2 participants due to anticoagulant use (*n*=1) or unwillingness to undergo the procedure (*n*=1). Skeletal muscle biopsies were not performed in 8 participants due to unwillingness to undergo the procedure (*n*=6), anticoagulant use (*n=*1), or getting unwell during the procedure (*n*=1). Due to logistical reasons, the DEXA could not be conducted in 3 individuals and was replaced by the BodPod®. Since DEXA and BodPod ® are distinct measuring techniques, BodPod® data were omitted from the analyses. Explorative analyses after pooling DEXA and BodPod® data did not alter conclusions (data not shown). Individual multisugar-test results were omitted from the final analyses if any problems during urine collection had occurred or the total amount of any of the sugars exceeded the provided amounts, suggesting failed abstinence from sweetener intake during the 24h collection period. Week 12 microbial data of one participant were excluded from the analyses due to the use of interfering antibiotics (doxycycline).

For logistical reasons, CID4 for one participant took place in a different clinical research department with different measuring devices. Additionally, one person was not fasted before CID2 as could be concluded from calorimetry data after the trial had ended. As a result, calorimetry and clamp data of both participants were therefore omitted from further analyses.

Supplementary Table 1. Prescribed content of high-protein diet and changes in dietary intake after adhering to a high-protein diet for 12 weeks in both intervention groups.

|  |  | **Placebo** | | | **Fiber** | | | **p-value** | | |
| --- | --- | --- | --- | --- | --- | --- | --- | --- | --- | --- |
|  | **Prescribed** | **Pre (*n*=16)** | **Week 6 (*n*=18)** | **Post (*n*=18)** | **Pre (*n*=15)** | **Week 6 (*n*=16)** | **Post (*n*=17)** | **Group** | **Time** | **Group * Time** |
| Kcal | 1670-2900 | 2208.9±648.3 | 2257.5±512.5 | 2167.8±525.4 | 1985.9±389.6 | 2260.6±668.3 | 2251.4±708.1 | 0.536 | 0.221 | *0.092* |
| MJ | 7-13 | 9.2±2.7 | 9.6±2.1 | 9.1±2.2 | 8.3±1.6 | 9.5±2.8 | 9.4±3.0 | 0.536 | 0.221 | *0.092* |
| CHO (g/day) | - | 226.6±65.1 | 221.4±50.6 | 197.4±46.3 | 200.6±57.8 | 229.3±60.5 | 220.1±71.5 | 0.954 | 0.298 | **0.034** |
| CHO (E%) | ±35% | 41.2±5.8 | 39.6±6.0 | 36.8±5.5 | 40.5±9.1 | 41.0±5.9 | 39.1±5.5 | 0.449 | **0.046** | 0.856 |
| Fat (g/day) | - | 95.8±39.5 | 91.8±35.5 | 97.4±37.4 | 86.2±26 | 91.4±39.3 | 93.3±35.4 | 0.501 | 0.717 | 0.543 |
| Fat (E%) | ±40% | 38.4±6.6 | 35.9±6.3 | 39.6±6.1 | 38.7±7.9 | 35.7±5.3 | 37.2±5.2 | 0.660 | *0.087* | 0.689 |
| Protein (g/day) | - | 86.6±31.7 | 108.7±23.9 | 100.9±21.6 | 80.0±17.4 | 104.4±34.3 | 110.8±35.1 | 0.722 | **<0.001** | *0.065* |
| Protein (E%) | ±25% | 16.0±3.5 | 19.6±4.1 | 19.0±3.9 | 16.1±1.6 | 18.7±3.5 | 19.9±2.6 | 0.981 | **<0.001** | 0.388 |
| Plant-based protein (%) | ±45% | 42.5±11.6 | 51.2±9.6 | 49.1±12.6 | 43.3±10.4 | 54.3±10.5 | 54.5±12.1 | 0.201 | **<0.001** | 0.700 |
| Animal-based protein (%) | ±55% | 57.6±11.6 | 48.8±9.6 | 50.9±12.6 | 56.7±10.4 | 45.7±10.5 | 45.5±12.1 | 0.201 | **<0.001** | 0.699 |
| Fiber* (g/MJ) | ±3.0 | 2.7±0.8 | 3.1±1.3 | 3.0±0.8 | 2.6±0.6 | 3.3±0.8 | 3.2±0.7 | 0.609 | **<0.001** | 0.689 |
| Alcohol (g/day) | - | 6.2±7.1 | 6.4±7.4 | 5.3±10.4 | 6.1±8.5 | 4.6±6 | 2.8±6 | 0.285 | 0.209 | 0.854 |

CID=Clinical Investigation Day, Kcal=Kilocalories, MJ=Megajoule, CHO=Carbohydrates, g=gram, E%=Energy percentage of total energy intake.*Fiber consumption is calculated from food diaries and does not include the additional fiber supplement of 15g/day.

Supplementary Table 2. Changes in physical activity, measured by a physical activity questionnaire (SQUASH) over time in both intervention groups.

|  | **Placebo** | | | **Fiber** | | | **p-value** | |
| --- | --- | --- | --- | --- | --- | --- | --- | --- |
|  | **Pre (n=21)** | **Week 6 (n=20)** | **Post (n=21)** | **Pre (n=19)** | **Week 6 (n=17)** | **Post (n=19)** | **Group * time** |  |
| Light (min/w) | 958.9±632.1 | 1120.6±856.7 | 1386.7±1135.1 | 1322.9±968.6 | 1369.5±253.6 | 1259.0±1051.8 | *0.081* |  |
| Medium (min/w) | 508.3±396.1 | 628.9±556.7 | 537.1±445.1 | 668.1±494.8 | 753.0±529.1 | 707.4±614.1 | 0.786 |  |
| Heavy (min/w) | 339.3±557.1 | 302.7±383.0 | 306.9±382.3 | 123.7±200.8 | 82.7±100.6 | 101.3±160.0 | 0.981 |  |
| Total score | 7142.5±4980.3 | 7786.7±3584.6 | 7934.4±3838.8 | 6932.4±2624.4 | 7142.86±3111.6 | 6847.1±3493.6 | 0.728 |  |

Data are presented as mean±SD. CID=Clinical Investigation Day

Supplementary Table 3. Changes in peripheral, hepatic and adipose tissue insulin sensitivity, and glucose disposal, measured by two-step hyperinsulinemic-euglycemic clamp after 12 weeks of fiber or placebo supplementation against the background of a high-protein diet.

|  | **Insulin infusion rate** | **Placebo (n=19)** | | **Fiber (n=18)** | | **p-value** | | |
| --- | --- | --- | --- | --- | --- | --- | --- | --- |
|  | **mU/m^2^/min** | **Pre** | **Post** | **Pre** | **Post** | **Group** | **Time** | **Group * Time** |
| Insulin-stimulated Rd (µmol/kg/min) | 40mU | 22.94±7.17 | 24.65±8.09 | 25.38±7.56 | 24.13±10.14 | 0.790 | 0.808 | 0.127 |
| Rd/insulin | 40mU | 0.060±0.026 | 0.066±0.030 | 0.062±0.026 | 0.057±0.025 | 0.611 | 0.913 | *0.081* |
| GIR (µmol/min) | 40mU | 2239.4±706.5 | 2442.5±784.2 | 2516.0±909.4 | 2284.8±989.3 | 0.927 | 0.977 | **0.034** |
| EGP (µmol/kg/min) | 0mU | 10.98±1.2 | 11.18±0.96 | 10.61±1.24 | 10.71±1.01 | 0.329 | 0.842 | 0.701 |
|  | 10mU | 5.8±2.2 | 5.78±1.99 | 5.11±2.2 | 5.05±1.85 | 0.425 | 0.879 | 0.968 |
| Insulin-stimulated EGP suppression (%) | 0-10mU | 47.5±18.2 | 48.8±16.7 | 53±18.7 | 53.1±16.2 | 0.578 | 0.971 | 0.955 |
| NOGD (µmol/kg/min) | 40mU | 14.00±5.92 | 14.09±5.93 | 15.02±6.26 | 13.91±7.87 | 0.956 | 0.664 | 0.507 |
| M-value | 40mU | 4.06±1.3 | 4.46±1.5 | 4.66±1.41 | 4.41±1.86 | 0.672 | 0.671 | *0.072* |
| Insulin-stimulated FFA suppression (%) | 0-10mU | 48.0±19.4 | 50.3±24.6 | 52.7±21.6 | 50.2±26 | 0.975 | 0.686 | 0.144 |

GIR=glucose infusion rate, EGP=endogenous glucose production, Rd=Rate of glucose disposal, NOGD=non-oxidative glucose disposal, FFA=free fatty acids. p-values are corrected for age, sex, BMI, and fat-free mass at baseline.

Supplementary Table 4. Changes in body weight, body fat distribution, blood pressure, and visceral adipose tissue after 12 weeks of fiber or placebo supplementation, combined with a high-protein diet.

|  | **Placebo (n=21)** | | | | **Fiber (n=19)** | | | | **p-value** | | |
| --- | --- | --- | --- | --- | --- | --- | --- | --- | --- | --- | --- |
|  | **Pre^#^** | **Week 2** | **Week 6** | **Post^#^** | **Pre^$^** | **Week 2** | **Week 6** | **Post^#^** | **Group** | **Time** | **Group * Time** |
| Weight (kg) | 100.1±16.8 | 99.6±16.8 | 99.4±16.6 | 100.1±16.2 | 97.6±14.4 | 97.2±14.7 | 97.2±14.7 | 97.8±15.3 | 0.277 | **0.013** | 0.846 |
| BMI (kg/m^2^) | 33.5±3.8 | 33.4±4 | 33.3±4 | 33.5±3.8 | 33.6±4.1 | 33.6±4 | 33.5±4 | 33.7±4.3 | 0.895 | **0.012** | 0.902* |
| Waist circumference (cm) | 110.3±12.8 | 110.3±12.4 | 110.4±11.5 | 110.4±12.1 | 110.1±11.8 | 110.6±11.5 | 109.6±11.7 | 109.6±12.7 | 0.841 | 0.701 | 0.624 |
| Hip circumference (cm) | 114.0±9.5 | 114.7±10 | 114.1±11.8 | 114.9±10.9 | 114.7±10.0 | 114.0±10.3 | 114.3±10.6 | 114.9±10.5 | 0.862 | 0.629 | 0.571 |
| WHR | 0.97±0.09 | 0.96±0.09 | 0.97±0.10 | 0.96±0.09 | 0.96±0.09 | 0.97±0.09 | 0.96±0.09 | 0.96±0.08 | 0.948 | 0.492 | 0.324 |
| SBP (mmHg) | 134±11 | 134±14 | 131±14 | 133±10 | 126±10 | 127±11 | 125±13 | 125±12 | **0.017** | 0.279 | 0.915 |
| DBP (mmHg) | 85±8 | 84±9 | 83±8 | 84±7 | 84±9 | 84±7 | 83±9 | 83±9 | 0.618 | 0.534 | 0.831 |
| Fat mass (kg) | 35.0±8.3 | - | - | 35.2±8.0 | 34.9±8.5 | - | **-** | 34.4±8.5 | 0.341 | 0.475 | 0.890 |
| Lean mass (kg) | 63.0±12.9 | - | - | 63.3±13.5 | 61.8±10.8 | - | - | 61.9±11.1 | 0.739 | 0.421 | 0.849 |
| Total fat % | 35.0±7.3 | - | - | 35.1±7.7 | 35.1±6.4 | - | - | 34.8±6.2 | 0.401 | 0.688 | 0.868 |
| A/G-ratio | 1.16±0.16 | - | - | 1.15±0.13 | 1.25±0.20 | - | - | 1.24±0.23 | **0.024** | 0.262 | 0.622 |
| VAT mass (g) | 889.6±297.4 | - | - | 863.8±249.3 | 977.6±368.7 | - | - | 991.3±388.4 | *0.077* | 0.651 | 0.580 |
| VAT % | 2.63±0.91 | - | - | 2.58±0.94 | 2.92±1.16 | - | - | 2.96±1.12 | **0.039** | 0.450 | 0.824 |

Data are presented as mean±SD. A/G-ratio=android/gynoid ratio, BMI=body mass index, CID=clinical investigation day, DBP=diastolic blood pressure, SBP=systolic blood pressure, VAT=visceral adipose tissue, WHR=waist-to-hip-ratio. ^#^DEXA n=18; ^$^DEXA n=19; *not adjusted for BMI.


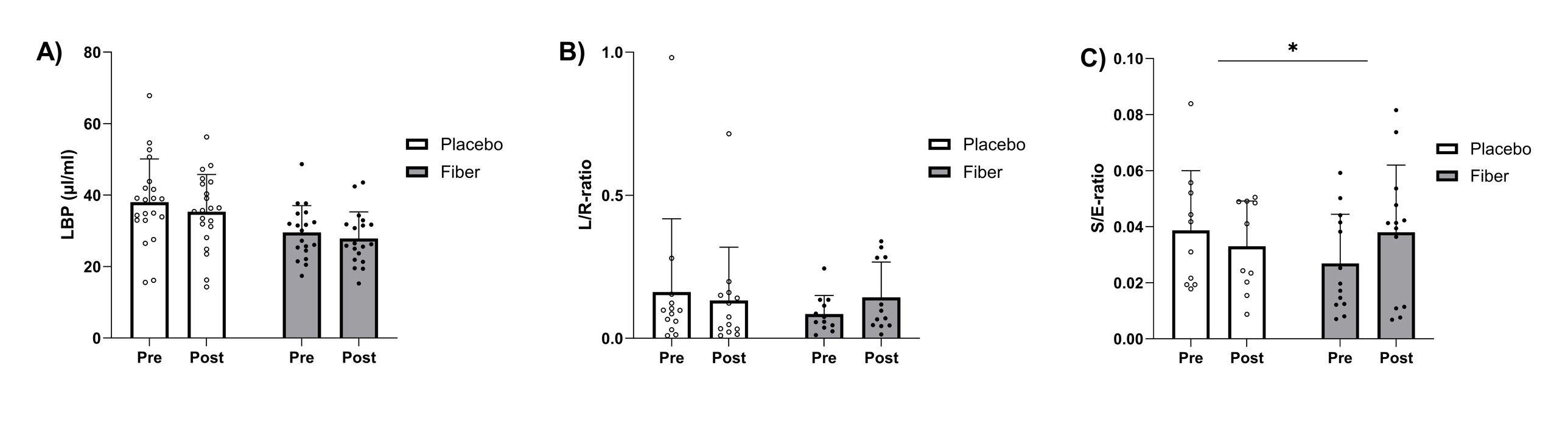
Supplementary Figure 2. Changes in gut permeability after 12 weeks of fiber supplementation versus placebo against the background of a high-protein diet. Presented p-values are interaction terms and are adjusted for age, sex, and BMI at baseline. **A.** Lipopolysaccharide-binding protein (LBP) (mean±SD, placebo n=21, fiber n=19), p=0.699. **B.** Lactulose-Rhamnose ratio (L/R-ratio) (mean±SD, placebo n=13, fiber n=12), p=0.378. **C.** Sucralose-Erythritol ratio (S/E-ratio) (mean±SD, placebo n=10, fiber n=13), p=0.046. *p<0.05.

Supplementary Table 5. Changes in gut permeability after 12 weeks of fiber or placebo supplementation against the background of a high-protein diet.

|  | Placebo | | | Fiber | | | *p*-value | | |
| --- | --- | --- | --- | --- | --- | --- | --- | --- | --- |
|  | **n** | **Pre** | **Post** | **n** | **Pre** | **Post** | **Group** | **Time** | **Group*Time** |
| *LBP (µl/ml)* | 21 | 38.03±12.10 | 35.37±10.37 | 19 | 29.57±7.47 | 27.85±7.44 | **0.007** | *0.081* | 0.699 |
| *L/R ratio* | 13 | 0.162±0.256 | 0.132±0.186 | 12 | 0.085±0.064 | 0.143±0.123 | 0.793 | 0.769 | 0.378 |
| *S/E ratio* | 10 | 0.039±0.021 | 0.033±0.016 | 13 | 0.027±0.018 | 0.038±0.024 | 0.513 | 0.499 | **0.046** |
| *Lactulose 0-5h (ng/ml)* | 13 | 6175±4589 | 5029±3064 | 12 | 5296±4400 | 8469±7509 | - | - | - |
| *Rhamnose 0-5h (ng/ml)* | 13 | 64422±36507 | 73919±59935 | 12 | 63782±33141 | 94790±94421 | - | - | - |
| *Sucralose 5-24h (ng/ml)* | 10 | 12397±12129 | 9904±6675 | 13 | 9403±6518 | 11897±7460 | - | - | - |
| *Erythritol 5-24h (ng/ml)* | 10 | 301347±186246 | 276280±103927 | 13 | 370592±188329 | 366213±180992 | - | - | - |

Data are presented as mean±SD. LBP=Lipopolysaccharide-binding protein; L/R=Lactulose/Rhamnose; S/E=Sucralose/Erythritol.

Supplementary Table 6. Explorative analyses of changes in plasma short-chain fatty acids (SCFA) and branched-chain fatty acids (BCFA) between weeks 0, 6, and 12 of fiber or placebo supplementation and a high-protein diet.

|  | **Placebo (n=21)** | | | **Fiber (n=19)** | | | **p-value** |  |
| --- | --- | --- | --- | --- | --- | --- | --- | --- |
|  | **Pre** | **Week 6** | **Post** | **Pre** | **Week 6** | **Post** | **Weeks 0-6-12** | **Weeks 0-6** |
| Total SCFA (μmol/l) | 41.97±19.50 | 29.96±44.24 | 50.47±33.76 | 70.25±37.79 | 31.33±27.58 | 72.15±49.50 | 0.147 | *0.055* |
| Total BCFA (μmol/l) | 2.87±1.62 | 3.95±2.55 | 3.58±2.18 | 3.65±1.89 | 3.16±1.39 | 3.92±2.10 | 0.113 | **0.040** |
| AA (μmol/l) | 39.29±18.87 | 27.74±42.71 | 47.70±33.41 | 66.62±36.86 | 27.51±27.39 | 68.77±9.56 | 0.125 | **0.044** |
| PA (μmol/l) | 1.85±0.94 | 1.53±0.94 | 1.92±0.67 | 2.20±0.96 | 1.81±0.94 | 2.27±0.79 | 0.968 | 0.834 |
| BA (μmol/l) | 0.46±0.45 | 0.31±0.52 | 0.47±0.45 | 0.47±0.39 | 0.36±0.38 | 0.53±0.49 | 0.911 | 0.762 |
| VA (μmol/l) | 0.03±0.05 | 0.02±0.04 | 0.02±0.04 | 0.07±0.15 | 0.02±0.05 | 0.04±0.04 | 0.317 | 0.422 |
| HA (μmol/l) | 0.33±0.38 | 0.36±0.58 | 0.36±0.29 | 0.88±1.97 | 0.37±0.29 | 0.56±0.40 | 0.376 | 0.236 |
| LA (μmol/l) | 629.75±252.07 | 714.15±415.25 | 617.40±233.56 | 578.05±251.58 | 571.23±260.38 | 551.82±178.06 | 0.668 | 0.403 |
| isoBA (μmol/l) | 0.83±0.63 | 1.04±0.65 | 0.98±0.68 | 0.99±0.59 | 0.82±0.44 | 1.11±0.69 | 0.168 | *0.068* |
| isoVA (μmol/l) | 0.55±0.34 | 0.83±0.69 | 0.76±0.55 | 0.87±0.39 | 0.62±0.35 | 0.84±0.52 | **0.009** | **0.004** |
| 2-MBA (μmol/l) | 1.49±0.71 | 2.08±1.30 | 1.84±1.03 | 1.78±1.01 | 1.71±0.70 | 1.97±0.95 | 0.234 | *0.093* |

Data are presented as mean±SD. AA=Acetic Acid, PA=Propionic Acid, BA=Butyric Acid, VA=Valeric Acid, HA=Hexanoic Acid, LA=Lactic Acid, isoBA=isoButyric Acid, isoVA=isoValeric Acid, 2-MBA=2-Methylbutyric Acid.


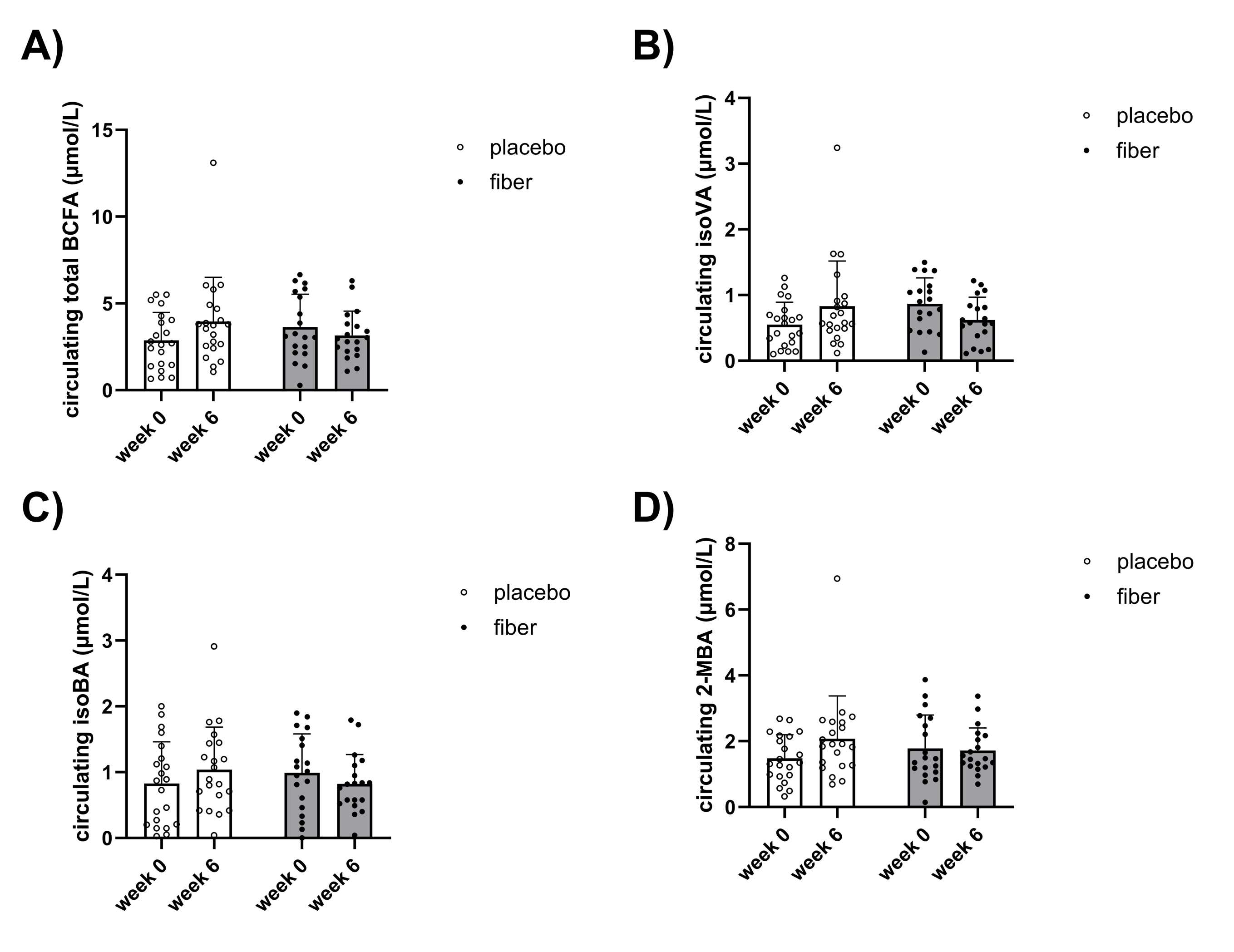


Supplementary Figure 3. Changes in circulating branched-chain fatty acids (BCFA) in the first 6 weeks of fiber or placebo supplementation, against the background of a high-protein diet. Data are presented as mean±SD. **A.** Total BCFA concentrations, **B.** Isovaleric Acid (IsoVA), **C.** Isobutyric Acid (IsoBA), **D.** 2-Methylbutyric Acid (2-MBA).


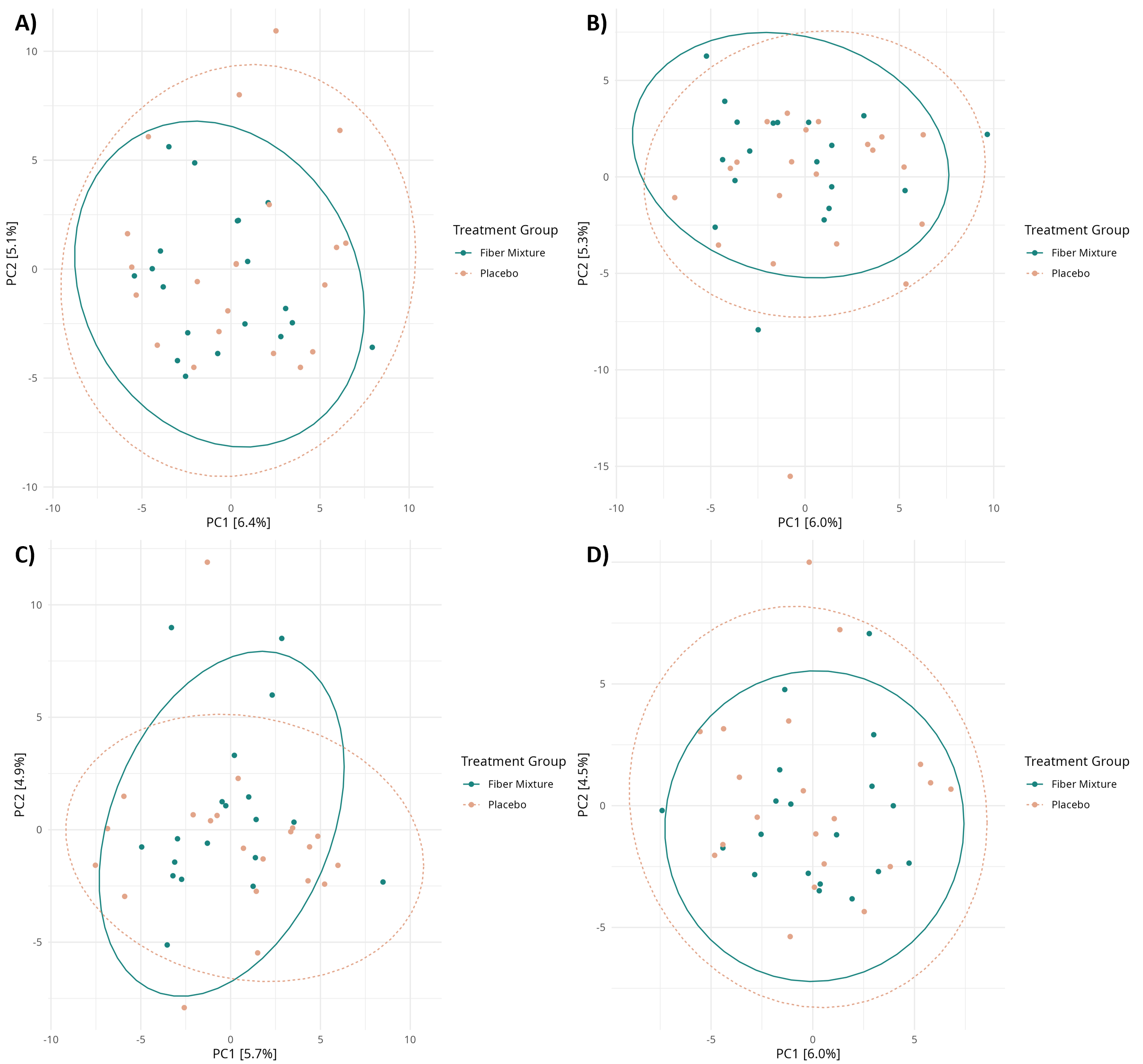


Supplementary Figure 4. Principal Component Analysis (PCA)-plots showing differences in microbial composition on ASV level between the groups after 0, 2, 6, and 12 weeks of fiber supplementation versus placebo, both within the context of a high-protein diet. **A.** Baseline. **B.** Week 2. **C.** Week 6. **D.** Week 12.


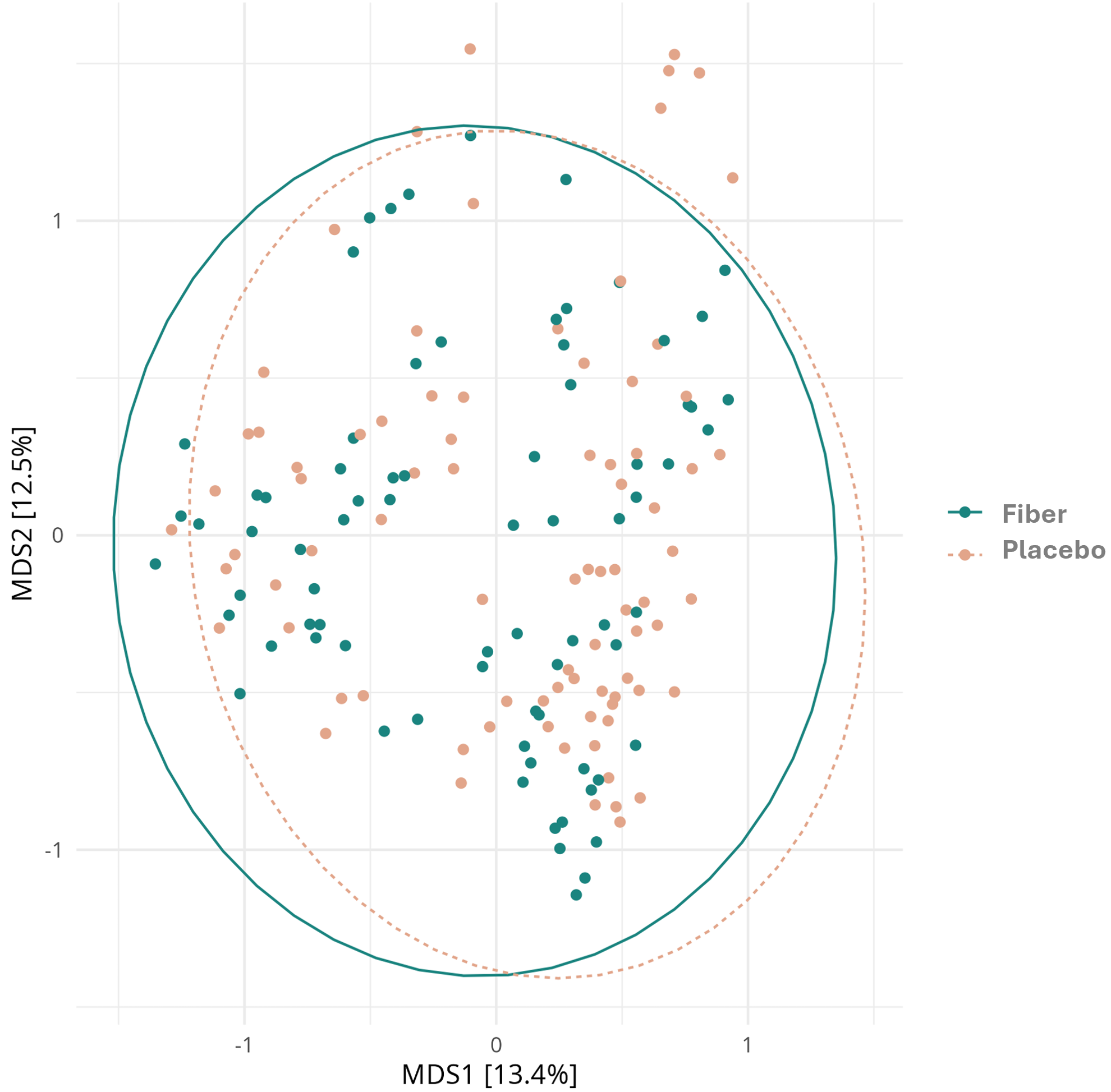


Supplementary Figure 5. Principal Coordinate Analysis (PCoA)-plot showing all results of individual Generalised UniFrac analyses between the two groups after 0, 2, 6, and 12 weeks of fiber supplementation versus placebo, both in context of a high-protein diet. PERmutational Multivariate ANalysis Of VAriance (PERMANOVA) of Generalized UniFrac distances at ASV level at weeks 0 (p=0.57), 2 (p=0.41), 6 (p=0.85), and week 12 (p=0.68) show no differences between the groups.


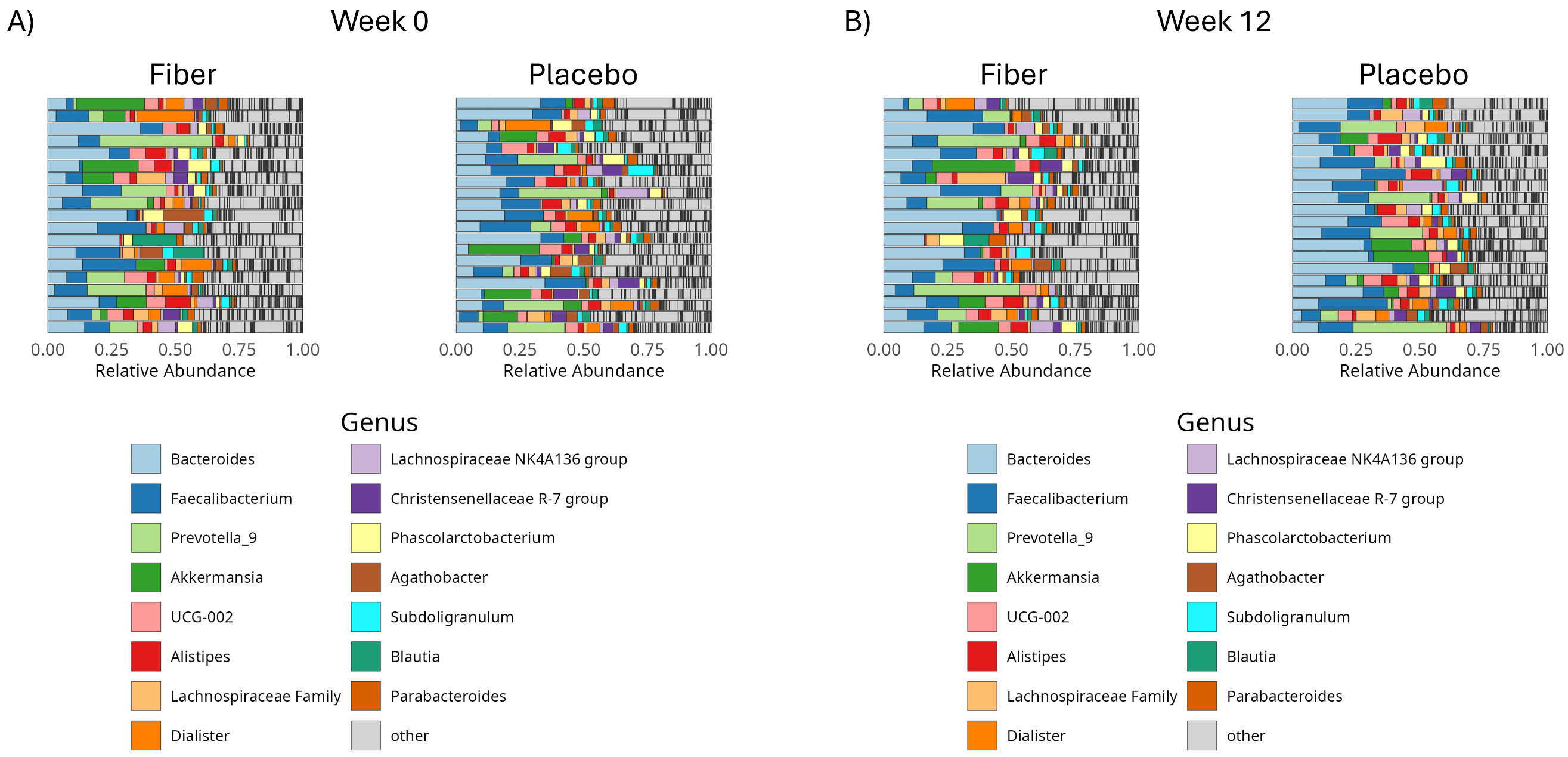


Supplementary Figure 6. Changes in relative abundance of the 15 most abundant microbial genera after 12 weeks of fiber supplementation or placebo and a high-protein diet. No significant changes between groups over time were observed in any of the taxa using linear mixed models with FDR-correction.


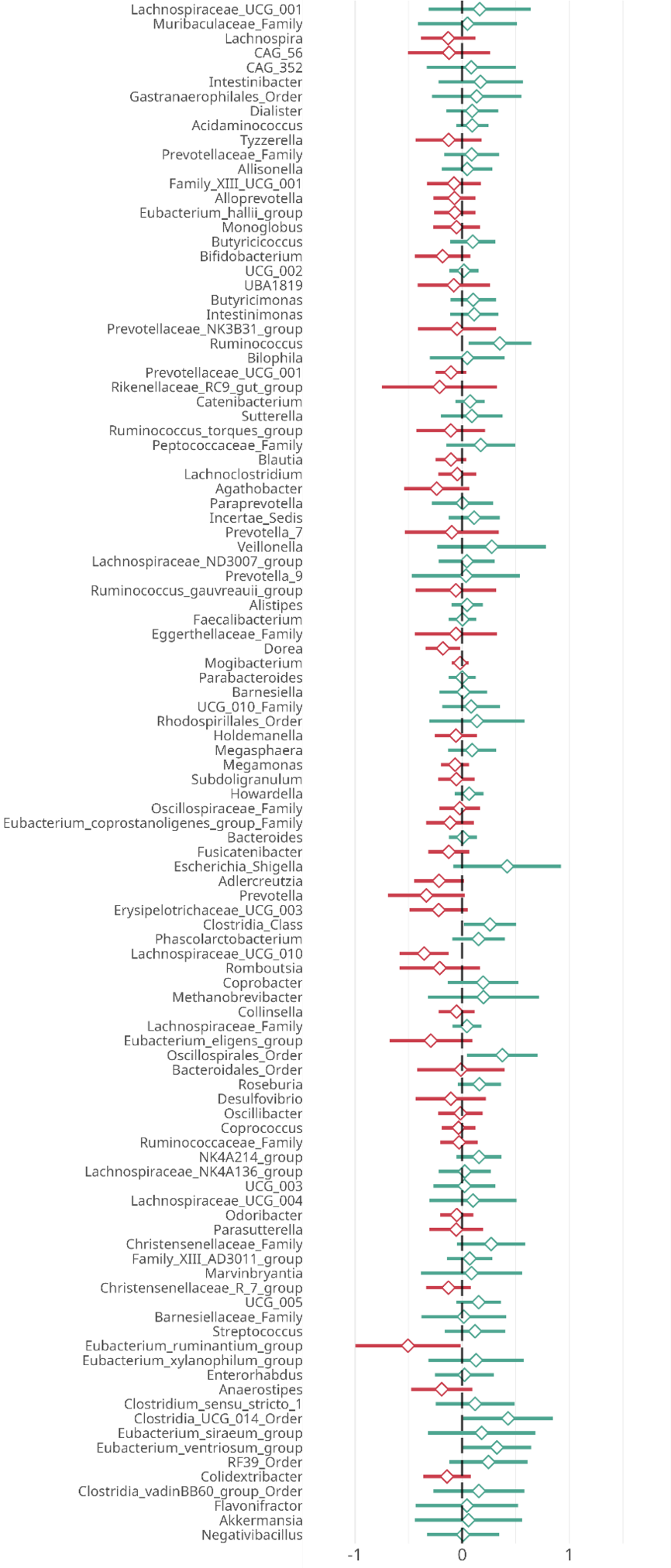


Supplementary Figure 7. Changes in relative abundance of microbial taxa comparing fiber versus placebo over time. Relative decreases are depicted in red, relative increases in green. No significant changes were found after FDR-correction.

|  | **Placebo** | | | **Fiber** | | | **p-value** | | |
| --- | --- | --- | --- | --- | --- | --- | --- | --- | --- |
|  | **Pre** | **Week 6** | **Post** | **Pre** | **Week 6** | **Post** | **Group** | **Time** | **Group * Time** |
| **RAND-36** |  |  |  |  |  |  |  |  |  |
| PCS | 78.7±3.4 | 84.9±3.3 | 81.7±4.0 | 77.9±3.6 | 76.6±3.6 | 74.5±4.2 | 0.227 | 0.396 | 0.228 |
| MCS | 79.6±3.1 | 85.3±2.1 | 82.2±3.9 | 80.3±3.3 | 80.5±2.3 | 74.3±4.1 | 0.303 | **0.026** | 0.226 |
|  |  |  |  |  |  |  |  |  |  |
| **PSS-10^#^** | 12.0±5.9 | - | 11.6±5.8 | 13.8±5.9 | *-* | 14.5±7.6 | 0.204 | 0.634 | 0.413 |

Supplementary Table 7. Changes in self-reported well-being (RAND-36), and perceived stress (PSS-10) after 12 weeks of fiber supplementation or placebo against the background of a high-protein diet.

MCS = mental component score, PCS = physical component score. *n=17, #p-value derived from generalized estimated equations, adjusted for age, sex, and BMI at baseline.

Supplementary Table 8. Changes in stool consistency are measured by the Bristol Stool Chart during and after 12 weeks of fiber or placebo supplementation within the context of a high-protein diet.

|  | **Placebo** | | **Fiber** | | **p-value** | | |
| --- | --- | --- | --- | --- | --- | --- | --- |
|  | **Mean±SD** | **n** | **Mean±SD** | **n** | **Group** | **Time** | **Group * Time** |
| CID1 | 4.0±1.1 | 19 | 3.7±1.1 | 21 | 0.622 | 0.355 | 0.279 |
| Week 2 | 3.9±1.1 | 18 | 4.0±0.9 | 20 |  |  |  |
| Week 6 | 3.7±0.9 | 17 | 3.7±0.7 | 20 |  |  |  |
| Week 9 | 4.0±1.1 | 17 | 4.1±1.3 | 19 |  |  |  |
| CID3 | 4.3±1.1 | 19 | 4.0±1.2 | 21 |  |  |  |

Generalized Estimated Equations (GEE) p-value is adjusted for age, sex, and BMI at baseline.

Supplementary Table 9. Changes in gastrointestinal symptoms, measured by the gastrointestinal symptom rating scale, after 12 weeks of fiber supplementation (F) or placebo (P), against the background of a high-protein diet.

|  | **n** | | **Stomachache** | | **Acid reflux** | | **Painful hunger** | | **Nausea** | | **Rumbling** | | **Burping** | |
| --- | --- | --- | --- | --- | --- | --- | --- | --- | --- | --- | --- | --- | --- | --- |
|  | **P** | **F** | **P** | **F** | **P** | **F** | **P** | **F** | **P** | **F** | **P** | **F** | **P** | **F** |
| CID1 | *21* | *19* | 1.4±1.8 | 1.5±2.8 | 0.8±1.5 | 0.6±1.1 | 0.1±0.5 | 0.0±0.0 | 0.2±0.6 | 0.9±2.2 | 0.9±1.4 | 2.1±2.8 | 0.6±1.8 | 0.8±1.4 |
| Week 2 | *20* | *18* | 1.0±1.4 | 1.7±2.6 | 0.9±1.9 | 0.3±1.0 | 0.2±0.7 | 0.0±0.0 | 0.5±1.0 | 0.9±2.2 | 0.5±1.0 | 1.6±2.2 | 0.5±1.1 | 1.3±2.5 |
| Week 6 | *20* | *17* | 1.2±2.0 | 1.6±2.7 | 1.2±2.0 | 0.9±2.3 | 0.4±1.0 | 0.2±0.7 | 0.4±0.8 | 0.8±1.4 | 1.1±1.9 | 1.5±1.5 | 0.6±1.1 | 0.9±2.5 |
| Week 9 | *19* | *17* | 1.2±2.1 | 0.9±2.4 | 0.5±1.9 | 0.9±2.5 | 0.1±0.5 | 0.1±0.5 | 0.4±1.2 | 0.8±2.5 | 0.8±1.2 | 1.7±2.6 | 0.5±1.4 | 1.5±2.8 |
| CID3 | *21* | *19* | 0.9±1.5 | 1.2±2.4 | 1.2±2.6 | 0.8±2.3 | 0.2±0.4 | 0.4±1.1 | 0.1±0.5 | 0.6±1.4 | 1.2±2.0 | 2.2±2.5 | 0.6±1.8 | 1.6±2.7 |
| p-value |  | | 0.383 | | 0.469 | | 0.428*^#^ | | 0.838 | | 0.842 | | 0.423 | |
|  |  | |  | |  | |  | |  | |  | |  | |
|  |  | |  | |  | |  | |  | |  | |  | |
|  | ***n*** | | ***Bloating*** | | ***Flatulence*** | | ***Constipation*** | | ***Diarrhea*** | | ***Urgency*** | | ***Residual*** | |
|  | **P** | **F** | **P** | **F** | **P** | **F** | **P** | **F** | **P** | **F** | **P** | **F** | **P** | **F** |
| CID1 | *21* | *19* | 1.4±2.3 | 1.9±3.1 | 1.5±1.9 | 3.2±3.3 | 1.1±2.1 | 0.4±1.4 | 0.5±1.3 | 1.0±2.2 | 1.1±1.7 | 1.4±2.4 | 0.1±0.2 | 2.2±3.1 |
| Week 2 | *20* | *18* | 1.7±2.6 | 3.2±3.2 | 1.7±2.2 | 4.8±3.1 | 0.5±1.5 | 1.4±2.2 | 0.5±1.1 | 1.1±2.4 | 0.5±1.4 | 2.7±2.7 | 0.3±1.3 | 1.4±1.8 |
| Week 6 | *20* | *17* | 1.4±2.6 | 1.8±1.9 | 2.3±2.8 | 4.2±3.0 | 0.5±0.9 | 0.8±1.2 | 0.5±0.9 | 0.8±1.5 | 1.4±2.3 | 2.9±3.5 | 0.2±0.5 | 1.4±2.1 |
| Week 9 | *19* | *17* | 0.4±0.8 | 2.2±2.8 | 1.7±2.2 | 4.2±3.3 | 0.2±0.7 | 0.8±1.4 | 0.6±1.3 | 1.4±2.8 | 1.5±2.4 | 3.1±3.8 | 0.2±0.7 | 1.8±2.7 |
| CID3 | *21* | *19* | 0.9±2.0 | 2.4±2.9 | 2.3±2.8 | 4.2±3.2 | 0.3±0.7 | 0.6±1.3 | 1.3±2.3 | 2.0±3.2 | 1.8±2.9 | 3.3±3.9 | 0.1±0.5 | 1.5±2.4 |
| p-value |  | | 0.231* | | 0.424* | | 0.115 | | 0.928^#^ | | *0.092* | | 0.509* | |

Generalized Estimated Equations (GEE) p-values are adjusted for age, sex, and BMI at baseline. ^*^Group p-value <0.05, ^#^Time p-value <0.05. CID = Clinical Investigation Day.

Supplementary Table 10. Changes in eating behavior after 12 weeks of fiber or placebo supplementation, both within the context of a high-protein diet.

|  | **Placebo (n=21)** | | **Fiber (n=19)** | | **p-value** | | |
| --- | --- | --- | --- | --- | --- | --- | --- |
|  | **Pre** | **Post** | **Pre** | **Post** | **Group** | **Time** | **Group*Time** |
| Cognitive restraint | 7.9±4.8 | 8.5±4.0 | 7.1±3.8 | 7.4±3.4 | 0.548 | 0.121 | 0.492 |
| Disinhibition | 5.6±3.5 | 5.4±3.2 | 7.4±4.3 | 7.6±5.1 | 0.119 | 0.869 | 0.741 |
| Hunger | 3.6±3.0 | 2.9±2.8 | 6.1±3.5 | 4.5±3.7 | **0.024** | **0.001** | 0.418 |

Data are presented as mean±SD. p-values are derived from generalized estimated equations (GEE) and are adjusted for age, sex, and BMI at baseline.

# References

1. van Deuren T, Umanets A, Venema K, et al. Specific dietary fibers steer toward distal colonic saccharolytic fermentation using the microbiota of individuals with overweight/obesity. *Food Res Int* 2025; 209: 116271.

2. American Diabetes Association. Diagnosis and classification of diabetes mellitus. *Diabetes care* 2010; 33: S62-S69.

3. World Health Organization. *Use of glycated haemoglobin (HbA1c) in diagnosis of diabetes mellitus: abbreviated report of a WHO consultation*. 2011. World Health Organization.

4. Trouwborst I, Gijbels A, Jardon KM, et al. Cardiometabolic health improvements upon dietary intervention are driven by tissue-specific insulin resistance phenotype: A precision nutrition trial. *Cell Metab* 2023; 35: 71-83 e75.

5. Saghaei M and Saghaei S. Implementation of an open-source customizable minimization program for allocation of patients to parallel groups in clinical trials. *J Biomed Sci Eng* 2011; 4: 734-739.

6. Altman DG and Bland JM. Treatment allocation by minimisation. *BMJ* 2005; 330: 843.

7. Hopewell S, Chan A-W, Collins GS, et al. CONSORT 2025 statement: updated guideline for reporting randomised trials. *BMJ* 2025; 389: e081123.

8. Gezondheidsraad. Voedingsnormen voor eiwitten; Referentiewaarden voor de inname van eiwitten. 2021.

9. Kromhout D, Spaaij CJ, de Goede J, et al. The 2015 Dutch food-based dietary guidelines. *Eur J Clin Nutr* 2016; 70: 869-878.

10. Pavlidou E, Papadopoulou SK, Seroglou K, et al. Revised Harris-Benedict Equation: New Human Resting Metabolic Rate Equation. *Metabolites* 2023; 13: 189.

11. DeFronzo RA, Tobin JD and Andres R. Glucose clamp technique: a method for quantifying insulin secretion and resistance. *Am J Physiol* 1979; 237: E214-223.

12. Weir JB. New methods for calculating metabolic rate with special reference to protein metabolism. *J Physiol* 1949; 109: 1-9.

13. Frayn KN. Calculation of substrate oxidation rates in vivo from gaseous exchange. *J Appl Physiol Respir Environ Exerc Physiol* 1983; 55: 628-634.

14. Most J, Goossens GH, Jocken JW, et al. Short-term supplementation with a specific combination of dietary polyphenols increases energy expenditure and alters substrate metabolism in overweight subjects. *Int J Obes (Lond)* 2014; 38: 698-706.

15. van Wijck K, Verlinden TJ, van Eijk HM, et al. Novel multi-sugar assay for site-specific gastrointestinal permeability analysis: a randomized controlled crossover trial. *Clin Nutr* 2013; 32: 245-251.

16. World Health Organisation Expert Consultation. Waist circumference and waist-hip ratio. *Report of a WHO Expert Consultation Geneva: World Health Organization* 2008; 2008: 8-11.

17. Wendel-Vos GC, Schuit AJ, Saris WH, et al. Reproducibility and relative validity of the short questionnaire to assess health-enhancing physical activity. *J Clin Epidemiol* 2003; 56: 1163-1169.

18. Aaronson NK, Muller M, Cohen PD, et al. Translation, validation, and norming of the Dutch language version of the SF-36 Health Survey in community and chronic disease populations. *J Clin Epidemiol* 1998; 51: 1055-1068.

19. VanderZee KI, Sanderman R, Heyink JW, et al. Psychometric qualities of the RAND 36-Item Health Survey 1.0: a multidimensional measure of general health status. *Int J Behav Med* 1996; 3: 104-122.

20. van Eck M, Berkhof H, Nicolson N, et al. The effects of perceived stress, traits, mood states, and stressful daily events on salivary cortisol. *Psychosom Med* 1996; 58: 447-458.

21. Cohen S, Kamarck T and Mermelstein R. A global measure of perceived stress. *J Health Soc Behav* 1983: 385-396.

22. Hewitt PL, Flett GL and Mosher SW. The Perceived Stress Scale: Factor structure and relation to depression symptoms in a psychiatric sample. *Journal of psychopathology and behavioral assessment* 1992; 14: 247-257.

23. Stunkard AJ and Messick S. The three-factor eating questionnaire to measure dietary restraint, disinhibition and hunger. *J Psychosom Res* 1985; 29: 71-83.

24. Black AE. Critical evaluation of energy intake using the Goldberg cut-off for energy intake:basal metabolic rate. A practical guide to its calculation, use and limitations. *Int J Obes Relat Metab Disord* 2000; 24: 1119-1130.

25. Goldberg GR, Black AE, Jebb SA, et al. Critical evaluation of energy intake data using fundamental principles of energy physiology: 1. Derivation of cut-off limits to identify under-recording. *Eur J Clin Nutr* 1991; 45: 569-581.

26. Andersen JR, Breivik K, Engelund IE, et al. Correlated physical and mental health composite scores for the RAND-36 and RAND-12 health surveys: can we keep them simple? *Health Qual Life Outcomes* 2022; 20: 89.

27. Surono IS, Widiyanti D, Kusumo PD, et al. Gut microbiota profile of Indonesian stunted children and children with normal nutritional status. *PLoS One* 2021; 16: e0245399.

28. Venema K, Verhoeven J, Verbruggen S, et al. Xylo-oligosaccharides from sugarcane show prebiotic potential in a dynamic computer-controlled in vitro model of the adult human large intestine. *Benef Microbes* 2020; 11: 191-200.

29. Martin M. Cutadapt removes adapter sequences from high-throughput sequencing reads. *EMBnetjournal* 2011; 17

30. Price MN, Dehal PS and Arkin AP. FastTree 2--approximately maximum-likelihood trees for large alignments. *PLoS One* 2010; 5: e9490.

31. Quast C, Pruesse E, Yilmaz P, et al. The SILVA ribosomal RNA gene database project: improved data processing and web-based tools. *Nucleic Acids Res* 2013; 41: D590-596.

32. Han J, Lin K, Sequeira C, et al. An isotope-labeled chemical derivatization method for the quantitation of short-chain fatty acids in human feces by liquid chromatography-tandem mass spectrometry. *Anal Chim Acta* 2015; 854: 86-94.

33. van Deuren T, Smolders L, Hartog A, et al. Butyrate and hexanoate-enriched triglycerides increase postprandrial systemic butyrate and hexanoate in men with overweight/obesity: A double-blind placebo-controlled randomized crossover trial. *Front Nutr* 2022; 9: 1066950.

34. Schols AM, Buurman WA, Staal van den Brekel AJ, et al. Evidence for a relation between metabolic derangements and increased levels of inflammatory mediators in a subgroup of patients with chronic obstructive pulmonary disease. *Thorax* 1996; 51: 819-824.

35. Orskov C, Rabenhoj L, Wettergren A, et al. Tissue and plasma concentrations of amidated and glycine-extended glucagon-like peptide I in humans. *Diabetes* 1994; 43: 535-539.

36. Torang S, Bojsen-Moller KN, Svane MS, et al. In vivo and in vitro degradation of peptide YY3-36 to inactive peptide YY3-34 in humans. *Am J Physiol Regul Integr Comp Physiol* 2016; 310: R866-874.

37. Kim S. ppcor: An R Package for a Fast Calculation to Semi-partial Correlation Coefficients. *Commun Stat Appl Methods* 2015; 22: 665-674.
