# Supplementary material for: Effects of a slowly fermentable fiber mixture against the background of a high-protein diet on insulin sensitivity and metabolic health in individuals with overweight: a randomized, placebo-controlled trial
Source: Gut Microbes. 2025 Dec 29;18(1):2606473. doi: 10.1080/19490976.2025.2606473 (PMC12758221; doi:10.1080/19490976.2025.2606473)
Supplement: Supplementary material — Study_protocol_DISTALstudy_MECapproved [file KGMI_A_2606473_SM6490.pdf]

Using a complex carbohydrate mixture added to a high-protein DIet to STeer fermentation and improve metabolic, gut and brain heALth: DISTAL-study

PROTOCOL TITLE Using a complex carbohydrate mixture added to a high-protein DIet to STeer fermentation and improve metabolic, gut and brain health: DISTAL-study

**Protocol ID**	**NL80459.068.22**
**Short title**	**DISTAL-study**
**EudraCT number**	**Not applicable**
**Version**	**1**
**Date**	**16-11-2021**
**Coordinating investigator/project leader**	**C.A.J. van Kalkeren, M.D.**<br>T: +31 (0)43 388 1638<br>E: c.vankalkeren@maastrichtuniversity.nl<br>Dept. Of Human Biology, Maastricht University, Maastricht, The Netherlands<br><br>**T.M. van Deuren, MSc**<br>T: +31 (0)43 388 1638<br>E: t.vandeuren@maastrichtuniversity.nl<br>Dept. Of Human Biology, MUMC+, Maastricht, The Netherlands
**Principal investigator(s) (in Dutch: hoofdonderzoeker/ uitvoerder)**	**Prof. Dr. E.E. Blaak**<br>T: +31 (0)43 388 1503<br>E: e.blaak@maastrichtuniversity.nl<br>Dept. Of Human Biology, Maastricht University, Maastricht, The Netherlands
**Sponsor (in Dutch: verrichter/opdrachtgever)**	**Maastricht University**
**Independent expert (s)**	**Dr. Z. Mujagic, Gastroenterologist-Hepatologist**<br>T: +31 (0)43 387 5100 (Polikliniek MUMC+)<br>E: zlatan.mujagic@mumc.nl

	Division of Gastroenterology-Hepatoly<br>Maastricht University Medical Center+ (MUMC+), Maastricht, The Netherlands
**Laboratory sites**	Laboratory of Human Biology, Maastricht University, Maastricht, the Netherlands<br>Metabolic Research Unit Maastricht, Maastricht University, Maastricht, The Netherlands
**Pharmacy**	Not applicable

PROTOCOL SIGNATURE SHEET

Name	Signature	Date
**Head of Department:**	**Prof. Dr. E.E. Blaak**<br>T: +31 (0)43 38 81503<br>E: e.blaak@maastrichtuniversity.nl<br>Dept. Of Human Biology, Maastricht University, Maastricht, The Netherlands	
**[Coordinating Investigator/Project leader/Principal Investigator]:**	**Prof. Dr. E.E. Blaak**<br>T: +31 (0)43 38 81503<br>E: e.blaak@maastrichtuniversity.nl<br>Dept. Of Human Biology, Maastricht University, Maastricht, The Netherlands	

TABLE OF CONTENTS

SUMMARY 11
1. INTRODUCTION AND RATIONALE 13
2. OBJECTIVES 16
3. STUDY DESIGN 17
4. STUDY POPULATION 21
4.1 Population (base) 21
4.2 Inclusion criteria 21
4.3 Exclusion criteria 21
4.3.1 Additional exclusion criteria for fMRI 22
4.4 Sample size calculation 22
5. TREATMENT OF SUBJECTS 23
5.1 Investigational product/treatment 23
5.2 Use of co-intervention 24
5.3 Escape medication 24
6. INVESTIGATIONAL PRODUCT 25
6.1 Name and description of investigational product(s) 25
6.2 Summary of findings from non-clinical studies 25
6.3 Summary of findings from clinical studies 25
6.4 Summary of known and potential risks and benefits 25
6.5 Description and justification of route of administration and dosage 25
6.6 Dosages, dosage modifications and method of administration 25
6.7 Preparation and labelling of Investigational Medicinal Product 25
6.8 Drug accountability 26
7. NON-INVESTIGATIONAL PRODUCT 27
7.1 Name and description of non-investigational product(s) 27
7.2 Summary of findings from non-clinical studies 27
7.3 Summary of findings from clinical studies 27
7.4 Summary of known and potential risks and benefits 27
7.5 Description and justification of route of administration and dosage 27
7.6 Dosages, dosage modifications and method of administration 27
7.7 Preparation and labelling of Non Investigational Medicinal Product 28
7.8 Drug accountability 28
8. METHODS 29
8.1 Study parameters/endpoints 29
8.1.1 Main study parameter/endpoint 29
8.1.2 Secondary study parameters/endpoints 29
8.1.3 Other study parameters (if applicable) 29
8.2 Randomisation, blinding and treatment allocation 29
8.3 Study procedures 30
8.3.1 Screening 30
8.3.2 Physical examination 30

8.3.3 Blood samples ... 31
8.3.4 Faecal samples ... 31
8.3.5 Multisugar test ... 31
8.3.6 Urine samples ... 32
8.3.7 Dual-energy X-ray absorptiometry (DEXA) scan ... 32
8.3.8 Functional Magnetic Resonance Imaging (fMRI) ... 32
8.3.9 Tissue biopsies ... 32
8.3.10 Two-step hyperinsulinemic-euglycemic clamp ... 33
8.3.11 Indirect calorimetry ... 34
8.3.12 Questionnaires ... 34
8.3.13 Neuropsychological assessment ... 36
8.3.14 Dietary intervention ... 37
8.3.15 Procedure justification ... 37
8.4 Withdrawal of individual subjects ... 38
8.4.1 Specific criteria for withdrawal ... 39
8.5 Replacement of individual subjects after withdrawal ... 39
8.6 Follow-up of subjects withdrawn from treatment ... 39
8.7 Premature termination of the study ... 39
9. SAFETY REPORTING ... 40
9.1 Temporary halt for reasons of subject safety ... 40
9.2 AEs, SAEs and SUSARs ... 40
9.2.1 Adverse events (AEs) ... 40
9.2.2 Serious adverse events (SAEs) ... 40
9.2.3 Suspected unexpected serious adverse reactions (SUSARs) ... 41
9.3 Annual safety report ... 41
9.4 Follow-up of adverse events ... 41
9.5 Data Safety Monitoring Board (DSMB) / Safety Committee ... 41
10. STATISTICAL ANALYSIS ... 42
10.1 Primary study parameter(s) ... 42
10.2 Secondary study parameter(s) ... 42
10.3 Other study parameters ... 42
10.4 Interim analysis (if applicable) ... 43
11. ETHICAL CONSIDERATIONS ... 44
11.1 Regulation statement ... 44
11.2 Recruitment and consent ... 44
11.3 Objection by minors or incapacitated subjects (if applicable) ... 44
11.4 Benefits and risks assessment, group relatedness ... 45
11.5 Compensation for injury ... 46
11.6 Incentives (if applicable) ... 46
12. ADMINISTRATIVE ASPECTS, MONITORING AND PUBLICATION ... 48
12.1 Handling and storage of data and documents ... 48
12.2 Monitoring and Quality Assurance ... 48
12.3 Amendments ... 48

12.4 Annual progress report ... 49
12.5 Temporary halt and (prematurely) end of study report ... 49
12.6 Public disclosure and publication policy ... 49
13. STRUCTURED RISK ANALYSIS ... 50
13.1 Potential issues of concern ... 50
13.2 Synthesis ... 52
14. REFERENCES ... 53

LIST OF ABBREVIATIONS AND RELEVANT DEFINITIONS

ABR	General Assessment and Registration form (ABR form), the application form that is required for submission to the accredited Ethics Committee; in Dutch: Algemeen Beoordelings- en Registratieformulier (ABR-formulier)
AE	Adverse Event
ACTH	Adrenocorticotropic hormone (ACTH)
ALAT	Alanine Aminotransferase
ANOVA	Analysis of variance
AR	Adverse Reaction
ASAT	Aspartate Aminotransferase
BCFA	Branched-chain fatty acid
BMI	Body Mass Index (Weight in kg / (Length in cm)^2)
BSS	Bristol Stool Scale
CA	Competent Authority
CANTAB	Cambridge Neuropsychological Test Automated Battery
CCC	Carbohydrate Competence Centre
CCMO	Central Committee on Research Involving Human Subjects; in Dutch: Centrale Commissie Mensgebonden Onderzoek
CID	Clinical Investigation Day
CRP	C-reactive protein
CV	Curriculum Vitae
CVD	Cardiovascular Disease
DEXA	Dual-energy X-ray absorptiometry
DSMB	Data Safety Monitoring Board
E%	Energy percentage
EGP	Endogenous glucose production
EU	European Union
EudraCT	European drug regulatory affairs Clinical Trials
FAA	Free fatty acid
FFQ	Food frequency questionnaire
fMRI	functional Magnetic Resonance Imaging
GCP	Good Clinical Practice
GDPR	General Data Protection Regulation; in Dutch: Algemene Verordening Gegevensbescherming (AVG)

GLP-1	Glucagon-like Peptide-1
GPR-x	G-protein coupled receptor
GRGS	Gastro-intestinal Symptom Rating Scale
HbA1c	Glycated haemoglobin
HDL	High-density lipoprotein
HOMA-IR	Homeostatic Model Assessment for Insulin Resistance
IB	Investigator's Brochure
IC	Informed Consent
IFN-γ	Interferon-gamma
IL-x	Interleukin-x
IMP	Investigational Medicinal Product
IMPD	Investigational Medicinal Product Dossier
LBP	Lipopolysaccharide Binding Protein
LDL	Low-density lipoprotein
METC	Medical research ethics committee (MREC); in Dutch: medisch-ethische toetsingscommissie (METC)
MRUM	Metabolic Research Center Maastricht
MUMC+	Maastricht University Medical Centre+
NAFLD	Non-alcoholic Fatty Liver Disease
NWO	Nederlandse organisatie voor Wetenschappelijk Onderzoek
PYY	Peptide YY
Ra	Rate of glucose appearance
(S)AE	(Serious) Adverse Event
scAT	Subcutaneous adipose tissue
SCFA	Short-chain fatty acid
SMT	Skeletal muscle tissue
SOP	Standard Operating Procedure
SPC	Summary of Product Characteristics; in Dutch: officiële productinformatie IB1-tekst
Sponsor	The sponsor is the party that commissions the organisation or performance of the research, for example a pharmaceutical company, academic hospital, scientific organisation or investigator. A party that provides funding for a study but does not commission it is not regarded as the sponsor, but referred to as a subsidising party.
SUSAR	Suspected Unexpected Serious Adverse Reaction
T2DM	Type 2 Diabetes Mellitus

**TAG**	**Triacylglycerol**
**TFEQ**	**Three-factor eating questionnaire**
**TNF-α**	**Tumor necrosis factor-alpha**
**UAVG**	**Dutch Act on Implementation of the General Data Protection Regulation; in Dutch: Uitvoeringswet AVG**
**UM**	**Maastricht University**
**VLDL**	**Very low-density lipoprotein**
**WMO**	**Medical Research Involving Human Subjects Act; in Dutch: Wet Medisch-wetenschappelijk Onderzoek met Mensen**

SUMMARY

Rationale: The prevalence of obesity and obesity-related disorders, such as metabolic syndrome, type 2 diabetes mellitus and non-alcoholic fatty liver disease, is rising worldwide. Apart from diet itself being of great influence on the development of these diseases, digestion and fermentation of dietary components are also of major importance. The gut microbiome is responsible for the fermentation of dietary components and its composition differs throughout the gastrointestinal tract. This leads to specific components being predominantly digested in the small intestine, while others are fermented in the colon. The majority of dietary components that are fermented in the colon are so called resistant carbohydrates or dietary fibres, which are carbohydrates that escape digestion, partly due to more complex chemical structures. It is known that this fermentation of indigestible carbohydrates, also referred to as saccharolytic fermentation, yields beneficial substrates, including short chain fatty acids, that improve immunity, and gut, metabolic and brain health, reduce inflammation, insulin resistance and gut permeability, and improve energy expenditure and satiety hormones. Nevertheless, indigestible carbohydrates are highly fermented in the proximal colon, due to the fact that these carbohydrates are the preferred fuel for the microbiota. Consequently, less indigestible carbohydrates are available for fermentation in the distal colon, and bacteria switch to the fermentation of indigestible proteins (proteolytic fermentation), instead. This yields more detrimental substrates, negatively impacting the human body and metabolism. However, optimal amounts of digestible and possible fermentable proteins in the small intestine are necessary to maintain proper small intestinal immunity and gut barrier function. Altogether, this gives rise to the necessity to ensure proper dietary component delivery to the different intestinal parts. In this study, a protein-carbohydrate combination will be provided that steers towards increased saccharolytic fermentation throughout the entire colon, thereby reducing proteolytic fermentation, with the aim to improve metabolic health in humans that are prediabetic and overweight.

Objective: To study the impact of a potato-pectin fibre mixture on insulin sensitivity, metabolic, gut and brain health and inflammation against the background of a high-protein diet.

Study design: Double-blind randomized placebo-controlled trial using a potato-pectin fibre mixtureversus a placebo (maltodextrin), against the background of a high-protein diet in all participants. Total duration: 12 weeks. A screening visit will be planned, eligibility will be determined and written informed consent will be obtained from participants, followed by two clinical investigation days (CID) to measure baseline characteristics. Afterwards, the dietary intervention trial starts. During the 12-week trial, 3 brief moments are planned to evaluate the trial and provide some additional data. After 12 weeks of intervention, two other CIDs are planned to measure endpoint outcomes. We will measure anthropometrics, insulin sensitivity,

energy harvesting and expenditure, substrate oxidation, body composition, gut permeability, microbiome composition and functionality, neurocognitive functioning, gene and protein expression in adipose and skeletal muscle tissue, physical activity, dietary intake and gastrointestinal complaints.

Study population: 44 overweight humans with impaired glucose homeostasis (male and female, age 30-75 years, BMI 28-40 kg/m^2, fasting glucose 5.6-6.9 mmol/l, HbA1c 42-47 mmol/mol), without any underlying diseases.

Intervention: Both groups will receive a high-protein diet (25% of energy intake (25E%)), along with either a potato-pectin fibre mixture or placebo (maltodextrin). Both dietary supplements will be taken three times daily for a total of 12 consecutive weeks.

Main study parameters/endpoints: The main endpoint is the change in tissue-specific insulin sensitivity, as measured by a two-step hyperinsulinemic-euglycemic clamp. Secondary outcomes are changes in energy and substrate metabolism (including production of SCFA and BCFAs), microbiome composition and functionality, gut permeability, brain health and inflammatory parameters.

Nature and extent of the burden and risks associated with participation, benefit and group relatedness: Participants will have to invest a considerable amount of time and dedication to follow a diet for 12 weeks and visit our facilities for a total of ~40 hours, divided over 8 different days. Furthermore, blood sampling (one sample on 4 separate occasions and multiple samples during the two-step clamp), the two-step clamp, the MRI measurements and tissue biopsies can cause mild discomfort upon the participants. Possible adverse effects of the biopsies include per-procedural pain, and hematomas and scarring afterwards. All measurements will be executed by experienced investigators to limit any experience of discomfort as much as possible. The clinical investigation days (CID) will be performed in the Metabolic Research Unit Maastricht (MRUM) and Maastricht University Medical Center+ (MUMC+) and a safety protocol and guidelines are in place in case of emergency.

There are no risks associated with the investigational product of this study. A potato-pectin fibre mixture has shown to be safe in the dose used here. The intervention itself will not cause any burden on a participant or threat to their health, but may rather have positive effects on health. However, mild gastro-intestinal discomfort can occur.

1. INTRODUCTION AND RATIONALE

The worldwide prevalence of obesity has nearly tripled in the past 40 years, totalling up to over 650 million adults in 2016.(1) Obesity is related to the onset of metabolic disorders, such as metabolic syndrome, type 2 diabetes mellitus (T2DM), non-alcoholic fatty liver disease (NAFLD) and cardiovascular disease (CVD). These diseases are associated with a high morbidity and mortality, making T2DM the ninth leading cause of death worldwide.(2) Apart from causing a high burden on a patients wellbeing, this also leads to a global health and socio-economic burden.

The human diet plays an important role in (metabolic) health and can be a major component in the aetiology of obesity, T2DM and NAFLD.(3-5) The western diet, being energy and relatively low in fibres, is seen as a major contributor to the development of these diseases. However, diet composition may affect food digestion and fermentation, which may have either a beneficial or detrimental effect on human metabolism and health.(3) The gut microbiome, consisting of trillions of microbes and being responsible for the fermentation of indigestible food components, is being more and more thoroughly understood, and is recognized for its important role in gut health, mucosal immunity and integrity, and in metabolic and psychological health of its host.(3-6)

The microbiome is able to ferment indigestible food components, such as prebiotics, dietary fibres and resistant starches. Fermentation of these carbohydrates, called saccharolytic fermentation, occurs mainly in the proximal colon and produces short-chain fatty acids (SCFA), of which acetate, propionate and butyrate are the most common.(4, 5) These SCFA, which are the main fuel for colonocytes, are absorbed quickly, leading to an almost absolute absence of SCFA in the transverse colon. In absence of indigestible carbohydrates, the microbiome shifts to proteolytic fermentation in the transverse and distal colon, producing numerous substrates, such as indoles, tryptophane, ammonia and hydrogen sulphide.(3, 7-9)

In general, SCFA have a beneficial effect on human health. They improve gut health by improving epithelial function, intestinal barrier function and mucosal quality, and reducing gut permeability. SCFA also have a positive impact on metabolic health by reducing body weight, increasing energy expenditure, satiety, insulin sensitivity and adipose tissue lipid buffering capacity, and preventing low-grade inflammation and ectopic lipid accumulation. Furthermore, evidence shows that an increase in circulating SCFA reduces inflammation in the gut, pancreas and throughout the body, including the brain, by reducing cytotoxic T cell activity and increasing anti-inflammatory regulatory T-cells. Lastly, SCFA are found to reduce stress in rodents, as a

result of SCFA-induced reduced release of corticotropin-releasing hormone and downregulation of mineralocorticoid receptor expression.(3-6, 8-19)

Proteolytic fermentation, more prevalent in the transverse and distal colon, produces substrates, such as branched-chain fatty acids, ammonia and phenols, that can have detrimental health effects.(5, 20, 21) On the other hand, there are also indications that substrates of proteolytic digestion may have positive health effects. Proteolytic digestion, and especially digestion of tryptophan rich proteins, in the small intestine is associated with reduced inflammation and improved gut barrier function and increased satiety and weight loss/maintenance.(3, 22-27) Overall, the balance between saccharolytic and proteolytic fermentation products in different parts of the gut may be an important determinant of metabolic health.

Our recent studies have shown that delivering SCFA, acetate in particular, specifically to the distal colon yields beneficial health effects, such as increased fat oxidation, reduced lipolysis, increased satiety hormone concentrations and reduced inflammatory cytokines (TNF-α).(28, 29) Apart from this, other studies have shown that delivering SCFA directly to the distal colon improves human metabolism and increases levels of satiety hormones and energy expenditure, which may be partly due to SCFA bypassing the liver and increasing peripheral SCFA concentrations.(28, 29) These effects were not present when acetate was administered to the proximal colon. We therefore hypothesize that an increased delivery of indigestible carbohydrates throughout the entire colon may have the most pronounced health effects by increasing distal saccharolytic fermentation and SCFA production, and thereby inhibiting proteolytic fermentation.

Increasing the SCFA concentration in the distal colon can be achieved by using complex carbohydrates, such as resistant starches or dietary fibres. These complex, indigestible carbohydrates escape fermentation in the small intestine, since the composition of the small intestinal microbiome differs from the colonic microbiome, lacking specific bacteria that are capable of fermenting these carbohydrates.(3-6, 8-10)

Many previous studies regarding manipulation of intestinal digestion and fermentation are performed on rodents, while evidence in humans is lacking. The few available human trials mainly describe acute interventions and present inconsistent data. Furthermore, the beneficial effects of SCFA on glucose homeostasis and insulin sensitivity are mainly found in lean individuals, and not in obese/(pre)diabetic adults.(28, 30-32)

The few human trials on long term effects of introduction of SCFA in the colon are mostly conducted in healthy adults, and results were not consistent. (33-35) This may be related to interindividual variation in response. We hypothesize that this is partly due to a different composition of initial microbiome, as well as differences in metabolic phenotypes (i.e. being insulin resistant or having prediabetes).

To this end, the aim of the present project is to determine the long-term effects (12 weeks) of an optimal balance between saccharolytic and proteolytic fermentation products on gut health, human metabolism and psychological state in humans with insulin resistance and/or prediabetes, and overweight. The present study might give rise to new possible preventative and therapeutic measures to reduce obesity and its related metabolic disorders, including insulin resistance, metabolic syndrome, type 2 diabetes mellitus and non-alcoholic fatty liver disease. Additionally, we expect that improving microbial composition and functionality may also improve brain health by supporting cognitive functioning and appetite regulation, altering reactions to food cues and reducing experienced stress.

2. OBJECTIVES

Primary Objective:

The main goal of this study is to provide evidence that steering saccharolytic/proteolytic fermentation by a specific indigestible fibre mixture supplemented against the background of a high-protein diet optimizes beneficial and minimizes detrimental substrate production. Most importantly, we will be looking into changes in peripheral insulin sensitivity.

Secondary Objectives:

Apart from determining possible effects on insulin sensitivity, we will also investigate the following parameters and analyse whether steering fermentation can influence the following secondary outcomes:

1. Hepatic and adipose tissue insulin sensitivity
2. Energy and substrate metabolism (energy harvest, energy intake, energy expenditure, fat and carbohydrate oxidation).
3. Circulating metabolites, inflammatory factors and blood lipid spectrum
4. Gut permeability
5. Brain health (neurocognitive functioning and food reward-related brain activity)
6. Adipose tissue and skeletal muscle metabolism
7. Composition and functionality of microbiome
8. Gastro-intestinal side-effects of provided dietary supplement against the background of a high-protein diet

Additionally, we will explore possible correlations in the gathered data, including anthropometric measurements, physical activity, dietary intake, glucose sensitivity, composition of gut microbiota, etc.

3. STUDY DESIGN

The proposed study will be a double-blind, randomized placebo-controlled trial, to evaluate the effect of a potato-pectin fibre mixture on gut, metabolic, immune and brain health by optimizing saccharolytic and proteolytic fermentation. Individuals aged 30-75 years with overweight or obesity (BMI ≥ 28 kg/m2 < 40 kg/m^2) and impaired fasting glucose (defined as fasting glucose 5.6-6.9 mmol/l or HbA1c 42-47 mmol/mol) or insulin resistance (HOMA-IR >1.85) will be included in the study.

All visits regarding this study will take place at the facilities of the department of human biology or the Metabolism Research Unit Maastricht of Maastricht University, or at the clinical facilities of Maastricht University Medical Center+.

After the application of an individual via (e-)mail or phone (see Chapter 4 for recruitment), the researcher will contact the potential participant by email or phone to provide additional information regarding the study, answer questions and determine possible eligibility, after which a week of consideration will be given. After a week, the potential participant will be contacted again and, if they are still interested in participating, a screening visit will be planned.

The first visit to our facilities will consist of the following components:

- Checking an individual's wellbeing, medical history and determining eligibility and safety of participation.
- Signing informed consent
- Measuring vital parameters and body measurements (body weight, length, waist-to-hip ratio, systolic and diastolic blood pressure)
- Taking blood samples to determine eligibility based on specific inclusion criteria (20ml to determine glucose, insulin, ALAT, creatinine, HbA1c)

The participant will visit our facilities after a >10h overnight fasted state.

All eligible participants will be equally randomized over the two arms (potato-pectin fibre mixture vs placebo) with stratification for age, sex and HbA1c (see Chapter 8.2). The entire trial period after screening and randomization will take 13 weeks, which will be explained in more detail below.

Intervention period

This randomized clinical trial with the primary outcome tissue-specific insulin sensitivity has a placebo-controlled, double blind, randomized parallel design, which allows evaluation of the role of a fibre mixture against the background of a high protein diet on host metabolism in male and female adult volunteers with overweight/obesity and impaired glucose homeostasis.

The approach is as follows:

1. Preceding this human intervention trial, a pilot study already has been performed to screen for the most optimal fibre mixture increasing distal colonic saccharolytic fermentation (and thus SCFA production) and inhibiting proteolytic fermentation. Faeces have been collected in 13 human volunteers with insulin resistance and overweight. The pooled faecal microbiota has been inoculated in TIM-2 to analyse SCFA and BCFA production after addition of different fibres or fiber combinations in a 24-h protocol against the background of a high-plant based protein mixture (which was comparable to the protein ratio as proposed in the human trial). The fibre mixture that resulted in the highest microbial SCFA production in the part of TIM-2 that represented the distal colon *in vitro* will be supplemented in the *in vivo* study. The hereby defined fibre mixture is composed out of potato and pectin fibres.
2. After randomization, the 12-week impact of a potato-pectin fibre mixture on tissue-specific insulin sensitivity will be determined in a randomized, double blind, parallel designed study.

Before the start of the intervention, participants will visit our facilities for the first clinical investigation days (CID 1 and 2). These days consist of the following test: (see Chapter 8.3 Study procedures for detailed description of the tests)

- Providing stool samples by participant
- Taking skeletal muscle and adipose tissue biopsies
- Performing two-step hyperinsulinemic-euglycemic clamp
- Performing indirect calorimetry during the clamp
- Performing gut permeability tests by intake of specific sugars dissolved in water and analysis of urine samples
- Conducting Functional Magnetic Resonance Imaging to study food-reward-related brain activity
- Conducting a DEXA scan to determine body composition
- Filling in questionnaires and performing neuropsychological tests
- Food diaries will be filled in prior to CID 1.
- Distributing and explaining of assigned intervention (a potato-pectin fibre mixture or placebo)

After CID 1, participants will be provided with a standardized meal. This meal needs to be consumed at home on the evening before CID 2 *ad libitum*. Afterwards, they should remain

fasted until the hyperinsulinemic-euglycemic clamp on CID 2. CID 2 will take place at least 2 days and preferably within 2 weeks after CID 1. In total, participants will visit the university after an overnight fast in week 6 and on all four CIDs.
After the 12 weeks of intervention, two additional CIDs (CID 3+4) will take place, which are similar to CID 1+2, respectively.

After completing the screening and CID 1+2, the intervention will consist of either a dietary supplement or a placebo which will have to be taken thrice daily during 12 consecutive weeks. Furthermore, all participants will receive a background diet high in protein 25EN% protein mixture, 30EN% fat, and 45EN% carbohydrates. Protein content will consist for 45-50% out of plant-based protein and for 50-55% out of animal-based proteins. The protein-rich products will be consumed during breakfast and dinner. The fibre mixtures and control product can be easily consumed with protein-rich food. The exact diet composition will be discussed individually to establish a diet suited to the participants' needs and taste. The energy requirements for this diet will be estimated using indirect calorimetry based on resting energy expenditure and an activity factor of 1.5.
Both the participants and investigators will be blinded to the treatment. The study is designed to study the dietary fibre effects against the background of a high protein diet independent of any diet or exercise intervention.

In week 2, 6 and 9, participants will return for a short interview or will be contacted via telephone to assess their wellbeing and compliance, answer arisen questions and fill in questionnaires. Additionally, a stool and blood sample will be provided in 6. Questionnaires will mainly be filled in before and after the intervention, with the addition of SQUASH and three-day food diary in week 6, and BSS and GSRS in weeks 2, 6 and 9.

To screen their food intake and physical activity (SQUASH), participants will fill in a three-day food diary before each CID 1+3 and on week 6. Gastrointestinal Symptom Rating Scale (GSRS) questionnaire will be completed the day before the CID1+3 and on weeks 2, 6 and 9. Therefore, we can screen whether the participant might experience gastrointestinal discomforts during the intervention period such as bloating and the outcomes are also relevant for our faecal microbiota analysis.

After completion of the trial, all data will be gathered and analyzed. Participants will receive a financial compensation for their efforts, as well as reimbursements of their travel expenses.
The total amount of time participants spend at our facilities will be around 36 hours (Screening: 1h; CID 1+2: 7+9.5h; Interview week 2, 6, 9: 3 x 0.5h; CID 3+4: 7+9.5h).

As mentioned before, CID 3 will be planned 12 weeks after the start of the intervention. The dietary supplement/placebo and the protein-rich diet will have to be continued until CID 3, which means that in some cases the intervention period will be not exactly 12 weeks, but a few days longer or shorter, depending on the availability of the participant. Logically, the investigators aim for CID 3 to be planned exactly 12 weeks after the start of the intervention. The reason the intervention needs to be continued until the day before CID 3, is to ensure possible acute effects of the intervention on the experiments/outcomes are comparable between participants. It is hypothesized that the small differences in total length of the intervention will not substantially interfere with the outcome measures.

<u>The researchers aim to adhere to the proposed schedule of the test days. However, if necessary, test day schedules might have to be altered due to logistical reasons.</u>

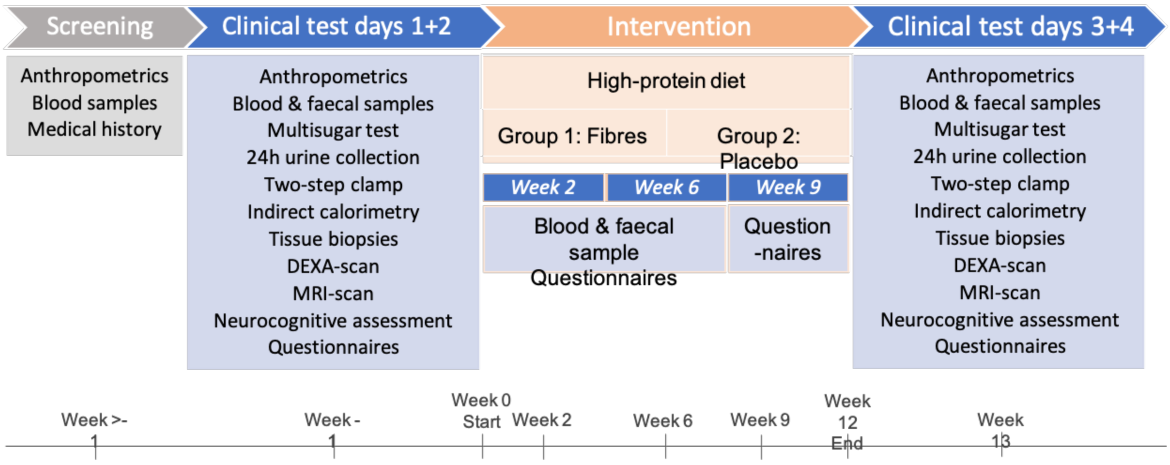

Figure 1. Study design

Tables 1 and 2. Clinical investigation day 1 and 2

Clinical investigation day 1	
08.00u	<u>Measuring</u> anthropometrics
08.15u	Multisugar test → start 24h urine collection
08.45u	fMRI
10.00u	Subcutaneous adipose tissue biopsy
10.45u	Questionnaires + CANTAB
12.15u	DEXA-scan
13.15u	Collect 5h urine

Clinical investigation day 2	
08.00u	Arrival after >10h fasting after standardized meal. <u>Providing of 24h-urine + faecal samples</u>
08.15u	Insertion cannulas, blood sampling
08.30u	Start clamp
09.00u	Skeletal muscle tissue biopsy
10.00u	First indirect calorimetry
13.00u	Second indirect calorimetry
15.30u	Third indirect calorimetry
16.30u	End clamp

4. STUDY POPULATION

4.1 Population (base)

Participants will be recruited using different types of media: posters will be hung in the university and hospital, a newspaper advertisement will be placed, social media such as LinkedIn and Facebook will be used, an online advertisement on proefpersonen.nl will be placed. Furthermore, participants from earlier trials who approved to being contacted for future studies will be contacted.

4.2 Inclusion criteria

A participant is considered eligible for this study if the following criteria are met:

- Age 30-75 years
- BMI ≥28 and <40 kg/m^2
- One or more of the following criteria to determine disturbed glucose/insulin homeostasis:
  - Fasting glucose 5.6-6.9 mmol/l
  - HbA1c 42-47 mmol/mol (6.0-6.5%) (36, 37)
  - HOMA-IR >1.85

4.3 Exclusion criteria

A potential subject that meets any of the following criteria will be excluded from participation in this study:

- Diabetes mellitus (type 1 or 2)
- Cardiovascular disease: Including no history of myocardial infarction, heart failure, arrhythmias (except hypertension (<160/100 mmHg is allowed)
- Pulmonary disease: no history of chronic obstructive pulmonary disease, emphysema, bronchitis, asthma
- Kidney disease/failure
- Hepatic disease/failure
- Gastrointestinal disease or a history of abdominal surgery (except appendectomy and cholecystectomy): No inflammatory bowel disease, irritable bowel syndrome and related diseases.
- Any other diseases affecting glucose and/or lipid metabolism
- Malignancy (except non-invasive skin cancer)
- Auto-immune disease
- Major mental disorders preventing correct participation (such as severe depression, psychosis, schizophrenia)
- Ongoing (infectious) disease or any disease with a life expectancy ≤5 years

- Substance abuse (nicotine abuse (including e-cigarettes) defined as >20 cigarettes per day; alcohol abuse defined as ≥8 drinks/week for females and ≥15 drinks/week for males(38); any drugs)
- A change in weight ≥3kg over the last 3 months or plans to lose weight or follow a hypocaloric diet during the study period
- Pre/pro/antibiotic use in the last 3 months or during the study
- Use of medication that influences glucose or fat metabolism and inflammation, such as:
  - Use of statins (stable use ≥3 months prior to and during study is allowed)
  - Use of antidepressants (stable use ≥3 months prior to and during study is allowed)
  - Use of anticoagulants (acetylsalicylic acid and carbasalate calcium are allowed)
  - Use of medication known to interfere with study outcomes
  - Use of β-blockers
  - Chronic corticosteroid treatment (>7 consecutive days)
- Regular use of laxatives 3 months prior to the study or during study period
- Change in physical activity or diet during study period
- Intensive physical activity (>3h per week)
- Pregnancy
- Following a vegan or vegetarian diet; presence of food allergies, intolerances or diet restrictions interfering with the study.

4.3.1 Additional exclusion criteria for fMRI

- Metal objects such as implants present in the body (e.g. electronic implants, pacemakers, metal fragments in the eyes, skin or body)
- The use of permanent make-up (eyeliners, eyebrows)
- Tattoos on the head, shoulders, breast or neck
- Claustrophobia

4.4 Sample size calculation

We used G-power to calculate the sample size required to detect a clinically relevant change in peripheral insulin sensitivity.

To calculate the sample size, we used the data derived from previous clinical trials of our research group using a comparable participant population to assess effects of alterations of the gut microbiota on peripheral insulin sensitivity with the hyperinsulinemic-euglycemic clamp technique. Based on the studies' data we calculated a within-group residual SD of 2.70, which was based on a SD of post measurement of 3.95 and a pre-post correlation of 0.73; sqrt(1-0.732)*3.95 = 2.70. Twenty-two (we aim to include an equal number of males and females: 11

males and 11 females) participants per group (N = 44) are needed to detect a physiological relevant difference of 20% (2.86µmol/kg/min) in the change in our primary outcome parameter peripheral insulin sensitivity measured via the hyperinsulinemic-euglycemic clamp technique, assuming a power of 80%, an α-value of .05, a within-group residual SD of 2.70 and a 20% dropout rate. The described secondary outcomes will be analysed exploratively, for microbiome analysis Benjamini-Hochberg procedure will be used to prevent false-positive results.

5. TREATMENT OF SUBJECTS

5.1 Investigational product/treatment

All participants will receive a high-protein diet during the entire trial (12 consecutive weeks in total, starting the day directly after CID 2 until the day prior to CID3). This diet will consist of 25 energy percentage (E%) protein, 30E% fat and 45E% carbohydrates. Proteins will be 45-50% plant-based and 50-55% animal-based, to ensure a resemblance to the current western diet consisting of mainly animal-based proteins, while simultaneously acting on the currently shifting trend in society towards more plant-based diets.

Furthermore, according to randomization, one group will receive a potato-pectin fibre mixture and the remaining participants will receive a placebo.

Potato-pectin fibre mixture

The potato-pectin fibre mixture showed the most positive results regarding short-chain fatty acid production in the distal colon in the TIM-2 model as described before. The fibre mixture will consist of 15 grams per day, consisting of 7.5 gram of potato fibre and 7.5 gram of pectin. The total of 15 grams will be equally divided over 3 doses, one for each meal.

Placebo

The participants not receiving a potato-pectin fibre mixture will be provided with a placebo in an isocaloric manner, which will be maltodextrin. Maltodextrin Glucidex IT 12 (Roquette Freres, Lestrem, France) is a fully digestible carbohydrate and will be used as isocaloric compensation to the investigated fibre additive. Furthermore, maltodextrin will be comparable to the fibre in terms of taste, mouthfeel and appearance.

5.2 Use of co-intervention

During the study period, participants should consume a protein-rich diet, of which the content, based on a subjects energy requirements and preferences, will be discussed individually. Participants should continue their regular behaviour in terms of physical activity and should refrain from using prebiotics, probiotics, antibiotics or other types of medication that can influence metabolism, glucose homeostasis or body weight. If participants visit a physician during the trial period, they should inform their physician of their partaking in this study.

5.3 Escape medication

No great adverse events are expected. Gastrointestinal discomfort is the main expected side effect, for which paracetamol may be used, if necessary, with a maximum of 1000 milligrams four times daily. In the event of a continuous necessity of paracetamol for more than 3 consecutive days, participants should contact the investigator for additional advices. In case of diarrhoea or constipation, no additional supplement may be taken without contacting the investigator for a suitable solution.

6. INVESTIGATIONAL PRODUCT

6.1 Name and description of investigational product(s)

The investigated fibre mixture will consist of potato fibre and sugar beet pectin. The daily ingested amount will be 15 grams (7.5 grams of potato fibre and 7.5 grams of pectin), equally divided over three gifts. Every gift will be taken alongside the three main mails (breakfast, lunch, dinner). Both fibres are commonly used in the Western/Dutch diet and are proven to be safe in these amounts. Required safety and analysis certificates are available and can be found in the additional files (D2).

6.2 Summary of findings from non-clinical studies

All relevant data on composition, safety, production and analyses can be found in additional files (D2).

6.3 Summary of findings from clinical studies

All fibres have been tested for safety purposes in several experiments. See attached GRAS and safety documents (D2).

6.4 Summary of known and potential risks and benefits

There are no known risks related to these dietary fibres. Potential risk or negative effects are related to gastro-intestinal discomfort as is described in chapter 9, 11 and 13. Potential benefits are clearly established in chapter 1, 2 and 11

6.5 Description and justification of route of administration and dosage

The fibre mixture shall be added to each meal (breakfast, lunch, dinner) three times a day (3 x 5 gram = 15 gram in total). This mixture can be added to prepared or raw food, but must not be added before preparation of a meal in order to maintain chemical composition. By adding it to or distributing over a meal, it can be ingested easily and quickly.

6.6 Dosages, dosage modifications and method of administration

The daily consumed dosage will be 15 gram (7.5 gram potato fibre + 7.5 gram sugar beet pectin), divided over 3 three doses of 5 grams. All will be added to a prepared or raw meal and will therefore be easily ingested.

6.7 Preparation and labelling of Investigational Medicinal Product

The intervention and placebo supplements will be provided in sachets. These sachets will be packed by an experienced, independent researcher (i.e. research intern, PhD-candidate) in the kitchen of the Metabolic Research Unit Maastricht. Blinding will be assured by using identical sachets for either groups, with only the preparing researcher knowing the exact content. This researcher will also distribute the sachets to the participant.
All sachets will be labelled, containing the following information:

- Study name (DISTAL-study)
- Participant code
- Date and time of intake
- Expiration date

6.8 Drug accountability

All supplement will be shipped to Maastricht University in a safe and recognizable way as designed by the manufacturer and will be stored at the Metabolic Research Unit Maastricht at room temperature. Participants will receive all required sachets for the entire trial period (i.e. 84 sachets for a daily suppletion for 12 weeks). Participants will return all empty sachets to determine compliance and for safe disposal at our facilities. If more sachets are needed for any reason, they will be provided by an independent researcher from our facilities. All sachets will be provided in a non-transparent bag.

7. NON-INVESTIGATIONAL PRODUCT

7.1 Name and description of non-investigational product(s)

Insulin and glucose will be used for the hyperinsulinemic-euglycemic clamp, which are regularly used products in clinical settings and are used as the golden standard for determination of insulin resistance. Lidocaine for local anesthesia is also regularly used in both clinical and research settings.

All are safe for human use. Further details can be found in attachment D2.

Gut permeability will be assessed with the Multisugar test (MS), performed by using specific orally administered sugars (~~1 gram sucrose~~, 1 gram lactulose, 1 gram sucralose, 1 gram erythritol, 0.5 gram rhamnose) which are all either commonly used sugars, European Food Safety Authority-approved artificial sweeteners or found to be safe for administration by previous studies.(39)

7.2 Summary of findings from non-clinical studies

Not applicable

7.3 Summary of findings from clinical studies

The usage of the multisugar test has been proven safe and effective to determine gut permeability.(39) All sugars were tested by Basic Pharma (Geleen, the Netherlands) to ensure safe oral human consumption. Furthermore, this test has been used successfully by our department before on multiple occasions, yielding reliable results without any adverse effects.

7.4 Summary of known and potential risks and benefits

There are no know or expected risks related to the oral ingestion of the related sugars. By consuming these sugars twice in our trial, we can measure the gut permeability to assess the effect of the intervention product on gut permeability.

7.5 Description and justification of route of administration and dosage

All sugars (4.5 gram in total per administration) will be dissolved in a single glass of water, which will be ingested orally on CID 1 and CID 3. This amount and way of ingestion is tested and proven reliable in previous studies.(39)

7.6 Dosages, dosage modifications and method of administration

Two-step hyperinsulinemic-euglycemic clamp:

- Insulin and glucose 20% (clamp): i.v. glucose 20% strongly dependent on the insulin sensitivity of the participant.
- [6,6-2H2]-glucose (clamp): i.v. prime concentration of 2.4 mg/kg body weight and a continuous infusion of 0.04 mg/kg/min for t= -120 to 330 minutes. The infusion will be set at a concentration that has no metabolic effect in humans (tracer amounts).
- Lidocaine (AT and skeletal muscle biopsies): 4-6 ml for local subcutaneous injection.

Gut permeability:

- 1 gram lactulose, 1 gram sucralose, 1 gram erythritol, 0.5 gram rhamnose for each administration. All will be dissolved in water and administered orally. No dosage modification is needed.

7.7 Preparation and labelling of Non Investigational Medicinal Product

Please find more detailed information in the attached documents D2.

All products used will be prepared in the kitchen or laboratories of the MRUM facilities.

Products to be used will be labelled as follows:

- Study name (DISTAL-study)
- Participant code
- Date and time of intake
- Expiration date

7.8 Drug accountability

Not applicable

8. METHODS

8.1 Study parameters/endpoints

8.1.1 Main study parameter/endpoint

The main endpoint is the difference in change in peripheral muscle insulin between intervention and control group, measured by hyperinsulinemic-euglycemic clamp.

8.1.2 Secondary study parameters/endpoints

Difference in changes in pre- and posttreatment values between intervention and control group, regarding the following parameters/endpoints:

- Hepatic and adipose tissue insulin sensitivity
- Energy and substrate metabolism (energy harvest, intake, expenditure, carbohydrate and fat oxidation)
- Body weight, composition and body fat distribution (anthropometrics, DEXA)
- Plasma and faecal concentrations of butyrate, propionate and acetate (SCFA) and proteolytic substrates (BCFA, indoles, ammonia)
- Plasma concentrations of inflammatory markers (IL-6, TNF-α, IL-1), circulating metabolites (glucose, triglycerides, FFAs) and hormones (insulin, GLP1, PYY, glucagon) and blood lipid spectrum (total cholesterol, HDL, triglycerides/TAG)
- Brain health: food reward related brain activation and cognitive function (CANTAB, fMRI)
- Gut permeability
- Faecal microbiota composition and functionality (Metagenome and -transcriptome sequencing)
- Gastrointestinal side effects (GRGS / BSS)
- Expression of gene and protein expression in adipose and muscle tissue

8.1.3 Other study parameters (if applicable)

- Three day food record: completed before CID 1 and 3 and the visit in week 6.
- Physical activity questionnaire: completed before CID 1 and 3 and visit in week 6 (SQUASH)
- Questionnaires on general wellbeing and quality of life, (family) medical history, stress, sleeping habits and fatigue, mood, satiety

8.2 Randomisation, blinding and treatment allocation

Participants will be randomized to the intervention or placebo group using a validated variable block randomization using Qminim. Furthermore, minimization will be used to ensure group comparability. Factors included for minimization are age, sex and BMI.(40) Initially, CASTOR

EDC would be used for randomization and stratification for this study. After the change from stratification to minimization, however, CASTOR cannot be used since it has no minimization option. Therefore, Qminim will be used, with which there is extensive experience within our research group.
Randomization and preparing and distribution of the, for participant and investigator, unrecognizable dietary supplement (either a potato-pectin fibre mixture or placebo) will be carried out by an independent researcher. This ensures that only the independent researcher knows which subject received which supplement, while both the participant and original investigator remain blinded to the treatment. In case of emergency, the independent investigator can deblind the participant(s).

8.3 Study procedures

Whenever participants are expected to be physically present at our facilities, they will be asked to travel by car or public transport to limit the physical activity prior to the experiments.
Prior to the screening and CIDs, subjects are asked to refrain from alcohol and strenuous physical activity 24h and need to arrive in the morning after a >10h overnight fasting period.

8.3.1 Screening

After recruitment (see Chapter 11.2 for additional information), a screening visit will take place. During this visit, the purpose and design of the study will be explained again and in- and exclusion criteria will be discussed, questions will be answered and additional information will be provided if necessary. If the person seems eligible, informed consent must be signed in the presence of the investigator before proceeding. After signing, the participant will be asked to provide an overview of his/her complete medical history and family history of diabetes mellitus, medication and substance use, recent weight changes, and an insight in their usual daily/weekly physical activity and diet. Furthermore, multiple questionnaires (see 8.3.10 Questionnaires) will be filled in, a physical examination will be performed and blood samples will be drawn (see below).

8.3.2 Physical examination

During the screening, on CID 1+3, in week 2 and 6, a physical examination will be performed, consisting of measurement of blood pressure and anthropometrics (length, weight, waist circumference, waist to hip ratio). Blood pressure will be measured three times; the first measurement will be used to acclimatize the participant to the measuring: data from this measurement will be omitted from analysis. All other body measurement will be taken twice to ensure correct data gathering and reduce variability.

8.3.3 Blood samples

During the study period, multiple blood samples will be taken, most of which will be drawn right before or during the hyperinsulinemic-euglycemic clamp. During the screening, fasting glucose, HbA1c, and liver and kidney function will be determined to assess eligibility. Also, on CID 2, CID 4 and in week 6, blood samples will be taken to assess the following elements:
Glucose, insulin, HbA1c, haemoglobin, leucocytes, C-reactive protein (CRP), interleukin 1 (IL-1), IL-2, IL-6, IL-8, IL-12, interferon-gamma (IFN-y), lipopolysaccharide binding protein (LBP), lipopolysaccharides (LPS), triacylglycerol (TAG), high-density lipoprotein (HDL), low-density lipoprotein (LDL), total cholesterol, glucagon-like peptide 1 (GLP-1), peptide YY (PYY), acetate, propionate, butyrate, valerate, isovalerate, isobutyrate, lactate, free fatty acids (FAA), branched-chain fatty acids (BCFA), leptin, aspartate amino transferase (ASAT), alanine amino transferase (ALAT), creatinine, ureum, zonulin, dopamine, FACS on immune cells.
Specific blood samples will be needed during the hyperinsulinemic-euglycemic clamp.
All samples consist of a maximum of 10ml blood per sample and will be analysed by the laboratories of the MUMC+. The total amount of blood needed is ~380ml (screening 20ml, CID 1+2 165ml (baseline 15ml, clamp 150), week 6 10ml each, CID 3+4 165ml (endpoint 15ml, clamp 150).

8.3.4 Faecal samples

Participants will hand in a faecal sample on CID 2+CID 4 and in week 2 and 6 to determine the amount of faecal saccharolytic and proteolytic fermentation substrates (e.g. SCFA, and BCFA and indoles, respectively), energy content and the composition of the microbiome, using 16S RNA sequencing. A sample container will be provided for every collection.

8.3.5 Multisugar test

Gut permeability will be assessed with the Multisugar test (MS), performed by using specific orally administered sugars (1 gram lactulose, 1 gram sucralose, 1 gram erythritol, 0.5 gram rhamnose) which are all either commonly used sugars, European Food Safety Authority-approved artificial sweeteners or found to be safe for administration by previous studies.(39) These sugars will be dissolved in water, ingested, absorbed throughout the gastrointestinal tract and eventually excreted in the urine. Higher urinary sugar concentrations indicate higher gut permeability. This test will be performed on either CID 1+3 or on CID 2+4, depending on logistics. The multisugar test will take place after an overnight fast, on the same CID as the fMRI. Participants will remain fasted for 5 hours after sugar ingestion.

8.3.6 Urine samples

Participants will collect 24-hour urine portions after the MS. Urine will be collected in for the first five hours after MS in one container, and from 5-24 hours after sugar ingestion in another. This allows assessment of gut permeability in the small and large intestine, respectively. Additionally, urinary nitrogen concentrations will be analysed to estimate protein oxidation in order to calculate carbohydrate and fat oxidation from the indirect calorimetry measures.

8.3.7 Dual-energy X-ray absorptiometry (DEXA) scan

On CID 1 and 3, a DEXA scan (effective radiation dose: ~20μSv) will be conducted to determine body composition (fat percentage, fat distribution, lean body mass), using facilities at MUMC+ (MUMC+, Discovery A, Hologic). There will be no discomfort of the DEXA scan.

8.3.8 Functional Magnetic Resonance Imaging (fMRI)

Apart from a DEXA scan, we will be using functional magnetic resonance imaging (fMRI) on CID 1 and 3 to determine changes in brain activity related to food reward before and after the intervention. A MRI scan is a non-invasive, commonly used radiological diagnostic technique. Apart from a rather narrow space in which the participant needs to be placed and a relatively noisy procedure, no discomfort will be present. In case of claustrophobia, this part might have to be omitted from the testing procedure or an eligible participant will not be included in the trial. Furthermore, hearing protection might be offered in necessary. In case of any fMRI-exclusion criteria are met (see chapter 4.3.1), the MRI will not take place.
Scanning will be performed on a 3T MRI scanner at the facilities of Scannexus. The scan will take place after an overnight fast. During scanning, images of different types of food and non-food will be displayed. A total set of 120 images will be used during the scan and will be presented in 12 blocks of 10 images for 2 seconds per image. Participants will be instructed to think about how much they like each shown subject. Between each block, there will be a 10 second break during which a fixation cross will be shown, as well as questions ensure participants maintain focused on the task. Images will consist of high-calorie foods (Fries, donuts, hamburgers etc) and low-calorie foods (vegetables, fruits etc). All images will be retrieved from a verified database.(41-44)

8.3.9 Tissue biopsies

On CID 1 and 3, after the DEXA scan, tissue biopsies will be taken from abdominal subcutaneous adipose tissue (scAT) and skeletal muscle tissue (SMT). In both tissues, the expression of genes, proteins and pathways related to adipogenesis, lipid metabolism oxidative metabolism and inflammatory profile will be analysed by standard PCR and blotting methodologies. Biopsies will be taken in >10h fasting state.

Adipose tissue biopsy

An abdominal subcutaneous adipose tissue biopsy will be performed using needle biopsy to obtain approximately 1 gram of adipose tissue. After administering of local anesthesia (lidocaine 2%), a needle biopsy will be performed.

Muscle tissue biopsy

A skeletal muscle biopsy will be taken from the *m. vastus lateralis*. After locally administered anesthesia (lidocaine 2%), the skin and fascia will be opened using a small incision. Afterwards, the biopsy needle will be inserted into the muscle to obtain several muscle samples using suction. A total of approximately 100mg will be collected. After the biopsy, the site of insertion will be closed using a Steristrip® and bandaged with Tegaderm® and Acrylastic® pressure bandage.

Besides changes in SCFA receptor GPR41/43 expression in these tissues, signalling pathways involved in insulin signalling and oxidative metabolism (i.e. phosphorylation of IRS1, PKC and AMPK, as well as PGC-1α and PPARα/γ), lipolysis (i.e. total and phosphorylated HSL, ATGL and its co-activator CGI-58 and PLIN1), and inflammation (i.e. NFκB, CD68, Interferon-γ and MCP) will be analysed in these samples using RT-qPCR and/or Western Blot. In this project we will focus on adipose tissue and skeletal muscle metabolism, since alterations in adipose tissue and muscle functionality could play a key role in the SCFA induced improved peripheral insulin sensitivity, by affecting lipolysis and proinflammatory processes in adipose tissue and fat oxidation in skeletal muscle. Additionally, adipocyte size will be determined. If budget allows, untargeted gene sequencing will be performed.

8.3.10 Two-step hyperinsulinemic-euglycemic clamp

To assess whole-body and tissue-specific insulin sensitivity, we will be performing a two-step hyperinsulinemic-euglycemic clamp, which is the gold standard to determine insulin sensitivity.(45) The clamp will be performed on CID 2 and 4 and will take place after a >12h overnight fasting period and a standardized meal the evening before. Before starting the procedure, an intravenous canula will be inserted in an antecubital vein to provide intravenous access for insulin and glucose suppletion. Another intravenous canula will be inserted on the dorsal side of the contralateral hand to ensure intravenous access for blood sampling.

At t = 0 minutes, a fasting blood sample (10ml) will be drawn, followed by infusion of a primed 6,6-$^2H_2$-glucose tracer at 0.04mg/kg/min, which will be used to calculate endogenous glucose production (EGP), glucose appearance (Ra) and glucose disposal. Therefore, blood samples (6ml) will be drawn at t=90, 105 and 120 min. At t = 120min, insulin will be infused at a low,

constant rate of 10 mU/m^2/min for 3 hours to allow determination of hepatic glucose production. After 3 hours (*t* = 300min), insulin infusion will be increased to 40 mU/m^2/min to inhibit EGP for 2.5 hours, providing insight in muscle insulin sensitivity. During insulin suppletion, plasma glucose levels will be measured every 5 minutes (1ml), upon which glucose (20% glucose solution) will be infused at 10-30ml/h to ensure a constant plasma glucose level of 4.8-5.2 mmol/l during the clamp. Afterwards, the total amount of infused glucose can be used to determine insulin sensitivity. Additional blood samples (6ml) will be drawn at *t* = 180, 240, 270, 285, 400, 420, 435 and 450 minutes.
At *t* = 450min, insulin infusion will be terminated and glucose infusion will be slowly reduced to zero to ensure a safe normalisation of glucose homeostasis. Additional dietary supplements will be provided (sandwiches, drinks) to prevent a hypoglycemic state.
During the clamp, an indirect calorimetry will be performed, which will be described below.

8.3.11 Indirect calorimetry

During the hyperinsulinemic-euglycemic clamp, an indirect calorimetry will be performed using a ventilated hood (Omnical, Maastricht Instruments, Maastricht). Measurements will take place during the basal, low insulin and high insulin infusion for 30 minutes (i.e. starting at *t* = 90, *t* = 270 and *t* = 420 minutes). During calorimetry, a hood will be placed over the subject's head to enable a continuous breath analysis for calculation of energy expenditure, and fat and carbohydrate oxidation. Data can then be used to determine resting metabolic rate, energy expenditure and total rate of fat and carbohydrate oxidation using the equations of Weir and Frayn.(46, 47) Nitrogen (N) excretion will be calculated based on the assumption that protein oxidation represents 15% of total energy expenditure. Additionally, urinary nitrogen will be analyzed as described before.

Calculations:
Energy Expenditure (EE) (kJ/min) = (3.9*VO2) + (1.1*VCO2)
Carbohydrate oxidation (CHO) (g/min) = (4.55*VCO2) – (3.21*VO2) – (2.87*N)
Fat oxidation (FAT) (g/min) = (1.67*VO2) – (1.67*VCO2) – (1.92*N)
N (g/min) = ((0.15* EE)/17)/6.25

8.3.12 Questionnaires

Multiple questionnaires will be used during the study period. The moment some or all these questionnaires will be used, are upon entering the study before CID 1, in week 2, 6 and 9 and before CID 3. The following questionnaires will be used:

Dietary habits

A 3-day food record will be filled in regarding dietary intake over three random days in the week prior to CID 1 and 3 and in week 6 (one weekend day and two weekdays). A mobile phone application, produced by the Dutch organisation Voedingscentrum, called Eetmeter, will be used. All products and quantities will be specified as specific as possible. Data will be gathered on CID 1 and 3 and in week 6.

Physical activity

Physical activity will be measured using the SQUASH questionnaire. Participants will be asked to maintain their usual physical behaviour. To ensure the amount of physical activity remains constant throughout the study period, the questionnaire will be filled in upon starting the study, in week 6 and at the end. Participants should refrain from any strenuous physical activity during the study period (≥4 hours per week).

Gastrointestinal symptom rating scale (GSRS)

To determine possible side effects due to the dietary components, the GSRS will be used. This questionnaire will be filled in at CID 1 and 3 and at week 2, 6 and 9 and contains information about the last 7 days, prior to the moment of measurement.

Bristol stool scale

The Bristol stool scale will be used alongside the GSRS at CID 1 and 3 and at week 2, 6 and 9 to determine possible changes in the consistency of produced faeces during the trial.

Three-factor eating questionnaire (TFEQ)

This questionnaire will be filled in to analyse neuropsychological responses to different types of food and food-related questions. 51 questions are divided in three subcategories: “cognitive restraint of eating”, “disinhibition” and “hunger” and will be scored on a binary scale.(41-43) The TFEQ will be filled in on CID 1 and 3.

fMRI Image validation

A small questionnaire, containing 30 of the 120 images (10 from each category) shown during the fMRI, will be filled in on or before CID1 to analyse to what extent the high- and low-calorie images are being liked and induce a feeling of hunger. This will be used to ensure that the hypothesized responses to the different food categories can be distinguished.

If applicable, some questionnaires can be filled in the day prior to CID 1 and 3.

8.3.13 Neuropsychological assessment

Cognitive performance

To assess potential effect of the intervention on brain health, an insight in cognitive function is needed, for which we will be using components of the *Cambridge Neuropsychological Test Automated Battery* (CANTAB).(48) The CANTAB will be performed on CID 1 and 3 and will last approximately 45 minutes.

The CANTAB will focus on attention, psychomotor speed, executive function and memory. Before the tests, all participants will be familiarised with an example and receive instructions in Dutch by the investigator. All tests of the CANTAB are computerized and presented on a device with touch screen (iPad 2017). Data are instantly and safely recorded. The test will in fixed order, as described below:

- Attention and psychomotor speed
  - Motor Screening Task: provides a general assessment of whether sensorimotor deficits or lack of comprehension, will limit the collection of valid data from the participant. Duration: approximately 2 minutes.
  - Reaction Time: provides assessments of motor and mental response speeds, as well as measures of movement time, reaction time, response accuracy and impulsivity. Duration: approximately 3 minutes.
- Executive function
  - Multitasking Test: assesses the participant's ability to manage conflicting information provided by the direction of an arrow and its location on the screen and to ignore task-irrelevant information. Duration: approximately 8 minutes.
  - Spatial Span: assesses visuospatial working memory capacity. Duration: approximately 5 minutes.
  - Executive function
  - Delayed Matching to Sample: assesses both simultaneous visual matching ability and short-term visual recognition memory, for non-verbalisable patterns. Duration: approximately 7 minutes.
  - Paired Associates Learning: assesses visual memory and new learning. Duration: approximately 8 minutes.

Wellbeing and stress

To assess a participants impression of its own wellbeing and stress, two additional questionnaires will be filled in: the *RAND 36-item Short Form Health Survey* (SF-36)(49, 50) and the and the *Perceived Stress Scale 10* (PSS-10)(51, 52), respectively. Both surveys will be completed on or in the week prior to CID 1 and 3.

8.3.14 Dietary intervention

At the end of CID 2, participants receive the assigned intervention (potato-pectin fibre mixture or placebo), in a blinded manner to both the participant and original investigator. Participants will start using the dietary supplement the day after CID 2 and continue to do so for 84 consecutive days. The assigned supplement should be consumed thrice daily during breakfast, lunch and dinner. Along with the dietary supplement, all participants should consume a diet high in proteins (see below). This diet will be composed in collaboration with a dietician to provide a diet suitable to each individuals needs and taste. Furthermore, subjects are asked to consume ≤1 alcohol units/day, based on the national dietary guidelines.(53)

Additionally, all participants will follow a diet high in proteins alongside the dietary supplement, of which the exact composition will be discussed individually to establish a diet suited to the participants' needs and taste. The energy requirements for this diet will be estimated using indirect calorimetry. The diet will consist of 25 energy percentage (E%) protein mixture, 30E% fat and 45E% carbohydrate. Proteins will be 45-50% plant-based and 50-55% animal-based, to ensure a resemblance to the current western diet consisting of mainly animal-based proteins, while simultaneously acting on the currently shifting trend into more plant-based protein-rich components.

8.3.15 Procedure justification

The screening procedure is necessary to determine eligibility and safety of participation, requiring a thorough medical history questionnaire, blood sampling and measuring of anthropometrics. Therefore, the described measures in the screening and physical examination section are required.

Blood sampling and faecal sampling are necessary to analyze differences from baseline in metabolic and inflammatory (serum) markers and microbiome functioning and composition (primary and secondary outcomes). Blood samples will be drawn and faecal samples will be provided on multiple occasions during the intervention period to analyze relatively acute and long-term effects of the intervention. The required amounts as described earlier are therefore needed to be able to analyze these effects. Blood sampling will bring a small burden to participants (apart from the risks that are described elsewhere (8.3.3 and 13.1, 13.2), because sample drawing can be done using the already intravenously placed cannulas that are needed for other experiments.

The multisugar tests, bringing a relatively low burden to participants, is necessary to analyze gut permeability. The execution of this test (ingestion of a sugar mixture and urine collection) is a rather low burden on participants in relation to the valuable data this provides. The

collected urine can additionally be used to complement indirect calorimetry data to determine protein oxidation.

The DEXA scan is the least invasive way to obtain accurate data on body composition, which is needed to answer secondary objectives (changes in body composition). Instead of DEXA, an MRI could be performed, which is more burdensome for some participants, time-consuming, and costly.

The fMRI as proposed in this protocol is the only way to establish food reward-related brain activity and is therefore essential. Additionally, to measure neurocognitive functioning, the CANTAB, which is a widely used test battery to analyze these parameters, will be used. To analyze stress-related symptoms, which can hypothetically, also be improved by increasing carbohydrate fermentation, specific stress-analyzing questionnaires will be used. Different aspects of neurocognitive/psychological functioning need to be investigated to be able to address the effects of the intervention on brain health. By excluding one of these procedures, an incomplete insight will be provided on this aspect.

Tissue biopsies are needed to analyze specific intracellular pathways, signalling and oxidation, as well as cell size. Taking biopsies is the only possible way to analyze these markers to answer secondary objectives.

The hyperinsulinaemic-euglycemic is the gold standard to assess peripheral, hepatic and adipose tissue insulin sensitivity. It is an invasive procedure, but allows optimally for analyzing insuline sensitivity in different tissues, as well as for comparing data to available literature worldwide. Additionally, the indirect calorimetry, which is performed during the clamp and is of minor importance regarding invasiveness, combined with urinary nitrogen analysis, is the only mechanism to analyze oxidation of the different macronutrients correctly.

Multiple questionnaires will be used: they are needed to determine adherence to the prescribed diet and physical activity restrictions, as well as establish estimated baseline carloric intake and energy expenditure. To assess side-effects, the GSRS and BSS are the least invasive techniques available.

In general, all described procedures and tests are needed to answer the primary and secondary study objectives as stated in section 8.1.

8.4 Withdrawal of individual subjects

Subjects can leave the study at any time for any reason if they wish to do so without any consequences. The investigator can decide to withdraw a subject from the study for urgent medical reasons.

8.4.1 Specific criteria for withdrawal

Apart from the opportunity for the subject to leave the study at any moment, a participant will also be withdrawn from the study in case of any situation making continuing of the study unsafe. Also, if one of the exclusion criteria is met during the study, withdrawal will follow.

8.5 Replacement of individual subjects after withdrawal

If a participant withdraws from the study, an alternative candidate may be selected to meet earlier established sample size/power.

8.6 Follow-up of subjects withdrawn from treatment

If a participant withdraws from the study, no follow-up is needed, unless a medical reason occurs for further follow-up. If medical assistance is needed, this will be provided by the investigator, if this falls within his/her capabilities. If needed, the participant will be referred to the correct caregiver.

8.7 Premature termination of the study

In case Maastricht University, government agencies or the METC AZM/UM stop the study for unexpected reasons, the project will be terminated. Unexpected reasons include the safety of the participant, due to unexpected effects of the study products as well as a condition that make it impossible to continue the research such as another viral pandemic (i.e. worsening of the Covid-19 situation) that strongly limit personal contacts. In case of premature termination, the METC AZM/UM will be notified within 15 days.

9. SAFETY REPORTING

9.1 Temporary halt for reasons of subject safety

In accordance to section 10, subsection 4, of the WMO, the sponsor will suspend the study if there is sufficient ground that continuation of the study will jeopardise subject health or safety. The sponsor will notify the accredited METC without undue delay of a temporary halt including the reason for such an action. The study will be suspended pending a further positive decision by the accredited METC. The investigator will take care that all subjects are kept informed.

9.2 AEs, SAEs and SUSARs

9.2.1 Adverse events (AEs)

Adverse events are defined as any undesirable experience occurring to a subject during the study, whether or not considered related to the investigational product. All adverse events reported spontaneously by the subject or observed by the investigator or his staff will be recorded.

9.2.2 Serious adverse events (SAEs)

A serious adverse event is any untoward medical occurrence or effect that

- results in death;
- is life threatening (at the time of the event);
- requires hospitalisation or prolongation of existing inpatients' hospitalisation;
- results in persistent or significant disability or incapacity;
- is a congenital anomaly or birth defect; or
- any other important medical event that did not result in any of the outcomes listed above due to medical or surgical intervention but could have been based upon appropriate judgement by the investigator.

An elective hospital admission will not be considered as a serious adverse event.

A medical doctor will be present or reachable by phone/pager at all times during a CID. If necessary, a doctor will provide medical assistance or a referral to the MUMC+ will be carried out. In life-threatening emergencies, regular national guidelines will always be followed.

The investigator will report all SAEs to the sponsor without undue delay after obtaining knowledge of the events.

The sponsor will report the SAEs through the web portal *ToetsingOnline* to the accredited METC that approved the protocol, within 7 days of first knowledge for SAEs that result in death or are life threatening followed by a period of maximum of 8 days to complete the initial preliminary report. All other SAEs will be reported within a period of maximum 15 days after the sponsor has first knowledge of the serious adverse events.

9.2.3 Suspected unexpected serious adverse reactions (SUSARs)

Not applicable

9.3 Annual safety report

Not applicable

9.4 Follow-up of adverse events

All AEs will be followed until they have abated, or until a stable situation has been reached. Depending on the event, follow up may require additional tests or medical procedures as indicated, and/or referral to the general physician or a medical specialist.

SAEs need to be reported till end of study within the Netherlands, as defined in the protocol

9.5 Data Safety Monitoring Board (DSMB) / Safety Committee

Not applicable

10. STATISTICAL ANALYSIS

The statistical analyses will be carried out by the investigator using SPSS software. Regarding descriptive statistics, numerical variables will be reported as mean ±SD. Categorical variables will be reported as numbers and percentages. To test for normality of the data, Histogram and Kolmogorov-Smirnov test will be used. In case, of non-parametric data, the data will be transformed using natural logarithm. In general, a p-value <0.05 will be considered statistically significant using two-tailed tests. In case of missing data, we will perform multiple imputation (MI) to estimate the missing value. MI is the process of replacing each missing data point with a set of m > 1 plausible values to generate m complete data sets. Multiple imputation has been shown to produce valid statistical inference that reflects the uncertainty associated with the estimation of the missing data.

10.1 Primary study parameter(s)

We will be comparing the intervention group versus the placebo group regarding the change in insulin sensitivity as measured by the two-step hyperinsulinemic-euglycemic clamp. Changes in glucose disposal rate and infused glucose concentrations before and after the 12 week intervention period are our primary study parameters and will be analysed and compared between the two groups. Data from both groups will be analysed using a linear mixed model to include repeated measures. An intention-to-treat and per-protocol analysis will be conducted. Intervention and study period will be set as fixed factors, participants will be set as random factor. Age and sex will be added as covariates in the analysis.

10.2 Secondary study parameter(s)

All additional gathered data will be analysed to answer our secondary objectives (Chapter 2), using the same methods as described above. We will be using linear mixed model for repeated measures, t-tests and regression models, depending on the specific data and requirements to assess each secondary objective. Anthropometrics, energy balance, substrate metabolism, urine and blood values will be presented as mean ± standard error.

Microbial composition

The microbiota composition based on 16S rRNA analysis or metagenome analysis will be determined from DNA isolated from faecal samples. Diversity of the microbiota will be calculated by i.e. Shannon-index. Moreover, to determine the effects of the intervention on for instance single bacterial groups, linear mixed model analysis will be conducted using an unstructured covariance structure for repeated measures.

10.3 Other study parameters

Not applicable

10.4 Interim analysis (if applicable)

Not applicable

11. ETHICAL CONSIDERATIONS

11.1 Regulation statement

The study has to be approved by the Medical Ethical Committee of the Maastricht University and academic hospital (medisch-ethische toetsingscommissie azM/UM, METC azM/UM). The study will be conducted according to the Declaration of Helsinki (64th WMA General Assembly, Fortaleza, Brazil, October 2013) and in accordance with the Medical Research Involving Human Subjects Act (WMO). The general principles of informed consent, ethics review and data management will be in line with GCP.

11.2 Recruitment and consent

Recruitment will take place via advertisements in local newspapers and social media, as well as posters hung around the Maastricht University and Maastricht University Medical Centre+. Furthermore, websites related to recruitment of participants for scientific trials will be used to attract participants. Additionally, if participants from other trials that were carried out within our research group have agreed to be contacted for future research will be contacted via email to ask request participation.

If a person is interested in participating, he/she can contact the investigators via email or telephone using the via advertisements provided contact details to express their interest. Eventually, the investigators will contact the possible participants via telephone to provide additional information, answer questions and discuss inclusion and exclusion criteria to enable the interested persons to determine whether they can and want to participate in this trial. Furthermore, the participant information documents will be provided via (e-)mail. Afterwards, participants will be given one week of contemplation before the investigators recontact the interested persons to answer questions, provide additional information if necessary and assess whether the person still wants to participate. If a person expresses continued interest, a screening visit will be planned to assess eligibility. During this visit, the investigators check whether all provided information is understood, answer arisen questions and provide additional information if necessary. If willingness to participate persists during this visit, the informed consent form will be signed in presence of the investigator. Afterwards, the screening will take place (see chapter 8.3.1). People that don't meet the inclusion or meet any of the exclusion criteria will not be included in the randomization and will be considered drop-outs.

11.3 Objection by minors or incapacitated subjects (if applicable)

Not applicable

11.4 Benefits and risks assessment, group relatedness

In general, obesity and its related diseases bring a high burden to a patients wellbeing and quality of life. Furthermore, these diseases are one of the leading causes of death worldwide, have a high socio-economic impact on society, and thus increase health care costs. By evaluating possible strategies to improve metabolic, gut and brain health, we want to attribute to a healthier lifestyle, a decrease in obesity and obesity-related diseases and in the socioeconomic impact of said diseases.

By testing the pre-established, possibly beneficial dietary component, we will gain insight in specific metabolic pathways, analyse interactions between food, gut and brain, and find potential ways to steer the human digestive system to provide beneficial substrates and reduce detrimental by-products. Along with the purpose of finding fundamental evidence to support further preventative or therapeutic interventions and studies, participants might benefit individually from the intervention in terms of small short-term improvements in body weight, body composition and carbohydrate, fat metabolism and emotional wellbeing.

Nor the investigated dietary additive, placebo or high-protein diet will pose any risk to general health in participants, since these are natural, commonly used dietary components and possible allergies or intolerances are established and ruled out during the screening visit. However, they might cause mild gastro-intestinal discomfort. Furthermore, adhering to a diet for 12 consecutive weeks, consisting of relatively high amounts of proteins and the investigated fibre-combination, requires motivation, compliance, time and willingness to change their normal lifestyle behaviour to a temporary lifestyle compliant with the prescribed intervention. This will subsequently pose a burden to participants, which is necessary to be able to evaluate the effects on primary and secondary outcome parameters as stated in chapter 8.

All other conducted tests will not pose a threat to the participants' health, but come with possible side-effects or complications. The physical examination, all questionnaires, collecting and providing of faecal and urine sample, neuropsychological evaluations, MRI and DEXA scan, the gut permeability test and indirect calorimetry will not harm the participants, nor pose a threat to their health. For all these tests, a participant will have to be cooperative and motivated to actively and correctly fulfil all tests and interventions, and will have to be willing to spend a vast amount of time at our facilities (40 hours in total). These tests will not pose a health risk for participants. However, the mental burden posed by these tests may be relevant. Especially the neurocognitive tests may have an impact on mental/psychological wellbeing, in particular when confronted with less optimal test results. Individual results will only be shared with the participant at their own request.

The induced radiation during a DEXA scan (<20 μSv) is far less than a Dutch citizen is exposed to on a yearly basis (2.5 mSv). This will not cause any additional health effects.
The MRI is performed in a narrow space and is known to be noisy. Some participants might experience claustrophobia, which will be examined during the screening visit, and during the scan, participants will be monitored closely.
The invasive tests (i.e. blood samples, tissue biopsies, hyperinsulinemic-euglycemic clamp) might cause more of a burden to a participant. Blood samples and intravenous cannulas will be drawn or inserted on multiple occasions, for which a sharp needle will need to penetrate skin tissue. This might hurt a participant or cause bleeding and hematoma, but is in general of minor relevance/importance/danger.
Tissue biopsies (adipose tissue and skeletal muscle) are the most invasive tests and might cause pain, hematoma, bruising or bleeding to a participant, mainly during and after the skeletal muscle biopsy. Pressure will be applied to the insertion site after the muscle biopsy to reduce the risk of hematoma and will be properly bandaged. The insertion site of both tissue biopsies will leave a small scar (~3mm for scAT and ~8mm for SMT).
During the hyperinsulinaemic-euglycemic clamp there is a small risk of hypo- or hyperglycemia. However, from our own extensive experience, these conditions do not occur very often and can be reversed immediately. A medical doctor is always available during the clamp.
Standard operating procedures (SOPs) for each measurement are available on the UM Human Biology Department's server.

11.5 Compensation for injury

The sponsor/investigator has a liability insurance which is in accordance with article 7 of the WMO.

The sponsor (also) has an insurance which is in accordance with the legal requirements in the Netherlands (Article 7 WMO). This insurance provides cover for damage to research subjects through injury or death caused by the study.
The insurance applies to the damage that becomes apparent during the study or within 4 years after the end of the study.

11.6 Incentives (if applicable)

Participants will receive a financial compensation for the invested time and energy. All subjects will participate voluntarily. After complete participation in the study, compensation will be

€500,-. Furthermore, travel expenses will be covered (€0.19/km). Some expenses for food products needed for the protein-rich diet can be declared as well. Premature termination of non-compliance will result in a reimbursement relative to the duration of participation. Participants will not receive any payment after only completing the screening, but their travel expenses will be reimbursed.

12. ADMINISTRATIVE ASPECTS, MONITORING AND PUBLICATION

12.1 Handling and storage of data and documents

The privacy of all (potential) participants will be protected and all data will be handled confidentially. All participants will be linked to a specific individual participant's number that will be used to identify participants and to link all data to throughout the study. The key that couples the participants personal data, such as name, address and date of birth, to the participants number will be stored in a password protect file, which can only be used by researcher directly involved in this study. This participants number will also be used to label all samples and other tests results. The participant's number will consist of a unique alpha-numeric combination, where the letters indicate this particular study for all participants (i.e. 'DS' for DISTAL-study) and a unique number for each participant (for example DS0001).

The researchers conducting the experiments and analyses have no access to the randomization lists that conceals the treatment code. The principal investigator Prof. dr. Ellen Blaak will receive sealed envelopes from Gabby Hul (independent person who performs randomisation), who has access to the randomization key, to reveal the treatment in case of medical emergency.

The handling of personal data will be in accordance with the EU General Data Protection Regulation and the Dutch Act on Implementation of the General Data Protection Regulation. The subjects will be given the opportunity to obtain information regarding their personal results of this study. This information will be given verbally and/or in writing.

When subjects give permission, collected material will be stored for 15 years at the Department of Human Biology or at the storage facility at Maastricht University Medical Centre+ (2nd Floor, UNS 50) after completion of this study to allow the possibility of additional analyses based on new techniques and advanced insights in the processes involved in the development of obesity and diabetes.

The retention period for the research data will be 15 years and the principal investigator, executive researchers, study monitor (Clinical Trial Center Maastricht) and controlling authorities such as Inspectie Gezondheidszorg en Jeugd will have the possibility to access these data if necessary.

12.2 Monitoring and Quality Assurance

Monitoring depends on the risk classification, which will be determined in consultation with Clinical Trial Center Maastricht after approval of this research protocol.

12.3 Amendments

Amendments are changes made to the research after a favourable opinion by the accredited METC has been given. All amendments will be notified to the METC that gave a favourable opinion.

12.4 Annual progress report

The sponsor/investigator will submit a summary of the progress of the trial to the accredited METC once a year. Information will be provided on the date of inclusion of the first subject, numbers of subjects included and numbers of subjects that have completed the trial, serious adverse events/ serious adverse reactions, other problems, and amendments.

12.5 Temporary halt and (prematurely) end of study report

The investigator/sponsor will notify the accredited METC of the end of the study within a period of 8 weeks. The end of the study is defined as the last patient's last visit.

The sponsor will notify the METC immediately of a temporary halt of the study, including the reason of such an action.

In case the study is ended prematurely, the sponsor will notify the accredited METC within 15 days, including the reasons for the premature termination.

Within one year after the end of the study, the investigator/sponsor will submit a final study report with the results of the study, including any publications/abstracts of the study, to the accredited METC.

12.6 Public disclosure and publication policy

There are no restrictions with respect to publication of the data. Both positive and negative results of the studies will be made public, preferably in peer-reviewed international scientific journals, according to the CCMO statement of publication policy. The authorship of the article shall be determined in appropriate consultation based on a considerable contribution to the set-up and execution of the study and an active participation in publication. Further, this study will be registered in a public trial registry before the first volunteer will be recruited.

13. STRUCTURED RISK ANALYSIS

13.1 Potential issues of concern

a. Level of knowledge about mechanism of action

As described before, fermentation of different components leads to different substrates, each with their own specific effect on the human body. In general, saccharolytic fermentation in the colon yields beneficial products (mainly SCFA) that improve human and gut health and metabolism, increase insulin sensitivity and energy expenditure, and reduce inflammation, gut permeability and stress.(4-6, 28-31, 54) Proteolytic fermentation, on the other hand, can either produce beneficial metabolites by fermenting protein in the small intestine, or more detrimental metabolites in the distal colon.(3, 6, 54) Increasing delivery of specific dietary components by adjusting dietary ratios of proteins, carbohydrates and fats, and switching to more complex carbohydrates can contribute to fermentation of specific, desired components in one place of the GI-tract, while another dietary component is fermented elsewhere. This way, it might be possible to increase beneficial and reduce detrimental dietary substrates through shifting from proteolytic to saccharolytic fermentation in the distal colon.

SCFA are known to bind to G-Protein-coupled-receptors 41 and 43 (GPR-41 and GPR-43). By doing so, they increase the release of hormones such as PYY and GLP-1, reduce inflammation by decreased plasma TNF-α concentrations. Furthermore, GLP-1 increases insulin secretion and sensitivity.(4-6, 10, 28, 30, 33-35)

In this study, we will only be using complex carbohydrates and proteins that are readily available in different types of food, frequently used in a regular Dutch diet.

Furthermore, the components in the high-protein diet will consist of products readily available in a regular Dutch diet.

b. Previous exposure of human beings with the test product(s) and/or products with a similar biological mechanism

Studies that supplemented SCFA or SCFA-esters are discussed in the *introduction* and in section A of this chapter. For additional information related to the investigated products, see attachments D2.

c. Can the primary or secondary mechanism be induced in animals and/or in *ex-vivo* human cell material?

The potato-pectin fibre mixture has been used in our TIM-2 model, mimicking colonic passage of this product in a human colon. This TIM-2 model used human faeces to simulate fermentation of a potato-pectin fibre mixture , after which the amount of produced SCFA was calculated. Among the potato-pectin fibre mixture, other protein/carbohydrate-combinations

were tested to determine which dietary component yields the most beneficial substrates. The potato-pectin fibre mixture was the most favourable and is therefore used in this trial. Hereby, we established the predicted effect of a potato-pectin fibre mixture on SCFA production. The effectiveness of the fibre supplement against a high-protein diet on (tissue-specific) insulin sensitivity has to be tested in a RCT in humans in order to obtain data that are relevant for prevention and treatment.

d. Selectivity of the mechanism to target tissue in animals and/or human beings

See chapter 13.1.a.

e. Analysis of potential effect

No harmful/detrimental effects are expected of this intervention. Only beneficial effects due to the mixture of complex fibres are to be expected. Potential adverse effect consist of gastro-intestinal discomfort, bloating, flatulence, constipation, diarrhoea etc, which will negatively impact a participants quality of life during the trial, but will not pose any serious threat to general health. Furthermore, after termination of the trial, these complaints will subside rather quickly.

f. Pharmacokinetic considerations

Not applicable

g. Study population

We will include 44 overweight, prediabetic people. Apart from these conditions, they should be healthy and possible health risks (other than those we are interested in, i.e. obesity and impaired glucose homeostasis) will be ruled out via our exclusion criteria.

h. Interaction with other products

The test products are food derived substances and are consumed in a regular human diet as well. No interaction with other products, posing a health risk to the individual of any kind, are expected.

i. Predictability of effect

As described before, we predict an increase in plasma and faecal SCFA concentrations, resulting in the beneficial effects (increased insulin sensitivity, energy expenditure; reduced inflammation, stress, gut permeability, etc)

j. Can effects be managed?

No adverse effect of the intervention is expected and, therefore, no rescue treatment will be needed. For all tests conducted on CIDs, strict guidelines are in place on how to respond in case of emergency and Standard Operating Procedures (SOP) are available for every test conducted.

13.2 Synthesis

There are no risks associated with the test products that can harm the participants. All tested substances are nutritional substances and impose no threat to general health. To reduce the possible health risks, we include healthy persons that meet all in- and exclusion criteria (Chapter 4).
All CIDs will be performed at the Metabolic Research Unit Maastricht (MRUM) at Maastricht University. All tests will be performed following general safety and health guidelines and Standard Operating Procedures (SOPs). Strict guidelines in case of emergency are in place. Furthermore, a medical doctor will always be either physically present or reachable via telephone during all tests.
All participants receive the phone numbers from the researchers and independent physician, which can be used to get in contact in case of emergency, complaints, questions or other complications.

14. REFERENCES

1. World Health Organization. Obesity and overweight - fact sheet 2021 [Available from: https://www.who.int/news-room/fact-sheets/detail/obesity-and-overweight.
2. World Health Organization. Diabetes - fact sheet 2021 [Available from: https://www.who.int/news-room/fact-sheets/detail/diabetes.
3. Blaak EE, Canfora EE, Theis S, Frost G, Groen AK, Mithieux G, et al. Short chain fatty acids in human gut and metabolic health. Benef Microbes. 2020;11(5):411-55.
4. Canfora EE, Jocken JW, Blaak EE. Short-chain fatty acids in control of body weight and insulin sensitivity. Nat Rev Endocrinol. 2015;11(10):577-91.
5. Canfora EE, Meex RCR, Venema K, Blaak EE. Gut microbial metabolites in obesity, NAFLD and T2DM. Nat Rev Endocrinol. 2019;15(5):261-73.
6. Blaak EE. Current metabolic perspective on malnutrition in obesity: Towards more subgroup-based nutritional approaches? Proc Nutr Soc. 2020;79(3):331-7.
7. Flint HJ, Duncan SH, Scott KP, Louis P. Links between diet, gut microbiota composition and gut metabolism. Proc Nutr Soc. 2015;74(1):13-22.
8. Miller TL, Wolin MJ. Pathways of acetate, propionate, and butyrate formation by the human fecal microbial flora. Appl Environ Microbiol. 1996;62(5):1589-92.
9. Morrison DJ, Preston T. Formation of short chain fatty acids by the gut microbiota and their impact on human metabolism. Gut Microbes. 2016;7(3):189-200.
10. Chambers ES, Byrne CS, Rugyendo A, Morrison DJ, Preston T, Tedford C, et al. The effects of dietary supplementation with inulin and inulin-propionate ester on hepatic steatosis in adults with non-alcoholic fatty liver disease. Diabetes Obes Metab. 2019;21(2):372-6.
11. Dalile B, Van Oudenhove L, Vervliet B, Verbeke K. The role of short-chain fatty acids in microbiota-gut-brain communication. Nat Rev Gastroenterol Hepatol. 2019;16(8):461-78.
12. Martin CR, Osadchiy V, Kalani A, Mayer EA. The brain-gut-microbiome axis. Cell Mol Gastroenterol Hepatol. 2018;6(2):133-48.
13. Reijnders D, Goossens GH, Hermes GD, Neis EP, van der Beek CM, Most J, et al. Effects of gut microbiota manipulation by antibiotics on host metabolism in obese humans: A randomized double-blind placebo-controlled trial. Cell Metab. 2016;24(1):63-74.
14. Silva YP, Bernardi A, Frozza RL. The role of short-chain fatty acids from gut microbiota in gut-brain communication. Front Endocrinol (Lausanne). 2020;11:25.
15. van de Wouw M, Boehme M, Lyte JM, Wiley N, Strain C, O'Sullivan O, et al. Short-chain fatty acids: Microbial metabolites that alleviate stress-induced brain-gut axis alterations. J Physiol. 2018;596(20):4923-44.
16. Vily-Petit J, Soty-Roca M, Silva M, Raffin M, Gautier-Stein A, Rajas F, et al. Intestinal gluconeogenesis prevents obesity-linked liver steatosis and non-alcoholic fatty liver disease. Gut. 2020;69(12):2193-202.
17. Zatterale F, Longo M, Naderi J, Raciti GA, Desiderio A, Miele C, et al. Chronic adipose tissue inflammation linking obesity to insulin resistance and type 2 diabetes. Front Physiol. 2019;10:1607.
18. Fransen F, Sahasrabudhe NM, Elderman M, Bosveld M, El Aidy S, Hugenholtz F, et al. B2→1-fructans modulate the immune system in vivo in a microbiota-dependent and -independent fashion. Front Immunol. 2017;8:154.
19. He Y, Wu C, Li J, Li H, Sun Z, Zhang H, et al. Inulin-type fructans modulates pancreatic–gut innate immune responses and gut barrier integrity during experimental acute pancreatitis in a chain length-dependent manner. Front Immunol. 2017;8(1209).
20. Macfarlane GT, Macfarlane S. Bacteria, colonic fermentation, and gastrointestinal health. J AOAC Int. 2012;95(1):50-60.
21. Sridharan GV, Choi K, Klemashevich C, Wu C, Prabakaran D, Pan LB, et al. Prediction and quantification of bioactive microbiota metabolites in the mouse gut. Nat Commun. 2014;5:5492.
22. Diether NE, Willing BP. Microbial fermentation of dietary protein: An important factor in diet¯microbe¯host interaction. Microorganisms. 2019;7(1).

23. Campos-Nonato I, Hernandez L, Barquera S. Effect of a high-protein diet versus standard-protein diet on weight loss and biomarkers of metabolic syndrome: A randomized clinical trial. Obes Facts. 2017;10(3):238-51.
24. Cuenca-Sánchez M, Navas-Carrillo D, Orenes-Piñero E. Controversies surrounding high-protein diet intake: Satiating effect and kidney and bone health. Adv Nutr. 2015;6(3):260-6.
25. Drummen M, Tischmann L, Gatta-Cherifi B, Adam T, Westerterp-Plantenga M. Dietary protein and energy balance in relation to obesity and co-morbidities. Front Endocrinol (Lausanne). 2018;9:443.
26. Postler TS, Ghosh S. Understanding the holobiont: How microbial metabolites affect human health and shape the immune system. Cell Metab. 2017;26(1):110-30.
27. Soenen S, Bonomi AG, Lemmens SG, Scholte J, Thijssen MA, van Berkum F, et al. Relatively high-protein or 'low-carb' energy-restricted diets for body weight loss and body weight maintenance? Physiol Behav. 2012;107(3):374-80.
28. Canfora EE, van der Beek CM, Jocken JWE, Goossens GH, Holst JJ, Olde Damink SWM, et al. Colonic infusions of short-chain fatty acid mixtures promote energy metabolism in overweight/obese men: A randomized crossover trial. Sci Rep. 2017;7(1):2360.
29. van der Beek CM, Canfora EE, Lenaerts K, Troost FJ, Damink S, Holst JJ, et al. Distal, not proximal, colonic acetate infusions promote fat oxidation and improve metabolic markers in overweight/obese men. Clin Sci (Lond). 2016;130(22):2073-82.
30. Freeland KR, Wolever TM. Acute effects of intravenous and rectal acetate on glucagon-like peptide-1, peptide yy, ghrelin, adiponectin and tumour necrosis factor-alpha. Br J Nutr. 2010;103(3):460-6.
31. Wolever TM, Brighenti F, Royall D, Jenkins AL, Jenkins DJ. Effect of rectal infusion of short chain fatty acids in human subjects. Am J Gastroenterol. 1989;84(9):1027-33.
32. Fernandes J, Vogt J, Wolever TMS. Intravenous acetate elicits a greater free fatty acid rebound in normal than hyperinsulinaemic humans. Eur J Clin Nutr. 2012;66(9):1029-34.
33. Todesco T, Rao AV, Bosello O, Jenkins DJ. Propionate lowers blood glucose and alters lipid metabolism in healthy subjects. Am J Clin Nutr. 1991;54(5):860-5.
34. Venter CS, Vorster HH, Cummings JH. Effects of dietary propionate on carbohydrate and lipid metabolism in healthy volunteers. Am J Gastroenterol. 1990;85(5):549-53.
35. Bouter K, Bakker GJ, Levin E, Hartstra AV, Kootte RS, Udayappan SD, et al. Differential metabolic effects of oral butyrate treatment in lean versus metabolic syndrome subjects. Clin Transl Gastroenterol. 2018;9(5):155.
36. Gillett MJ. International expert committee report on the role of the a1c assay in the diagnosis of diabetes: Diabetes care 2009; 32(7): 1327-1334. Clin Biochem Rev. 2009;30(4):197-200.
37. World Health Organization. Use of glycated haemoglobin (HbA1c) in diagnosis of diabetes mellitus: Abbreviated report of a WHO consultation. World Health Organization; 2011.
38. Centers for Disease Control and Prevention. Alcohol use and your health 2021 [updated May 11, 2021. Available from: https://www.cdc.gov/alcohol/fact-sheets/alcohol-use.htm.
39. van Wijck K, Verlinden TJ, van Eijk HM, Dekker J, Buurman WA, Dejong CH, et al. Novel multi-sugar assay for site-specific gastrointestinal permeability analysis: A randomized controlled crossover trial. Clin Nutr. 2013;32(2):245-51.
40. Kim YS, Unno T, Kim BY, Park MS. Sex differences in gut microbiota. World J Mens Health. 2020;38(1):48-60.
41. Drummen M, Dorenbos E, Vreugdenhil ACE, Stratton G, Raben A, Westerterp-Plantenga MS, et al. Associations of brain reactivity to food cues with weight loss, protein intake and dietary restraint during the preview intervention. Nutrients. 2018;10(11).

42. Drummen M, Dorenbos E, Vreugdenhil ACE, Raben A, Westerterp-Plantenga MS, Adam TC. Insulin resistance, weight, and behavioral variables as determinants of brain reactivity to food cues: A prevention of diabetes through lifestyle intervention and population studies in europe and around the world - a preview study. Am J Clin Nutr. 2019;109(2):315-21.
43. Stunkard AJ, Messick S. The three-factor eating questionnaire to measure dietary restraint, disinhibition and hunger. J Psychosom Res. 1985;29(1):71-83.
44. Blechert J, Lender A, Polk S, Busch NA, Ohla K. Food-pics_extended-an image database for experimental research on eating and appetite: Additional images, normative ratings and an updated review. Front Psychol. 2019;10:307.
45. DeFronzo RA, Tobin JD, Andres R. Glucose clamp technique: A method for quantifying insulin secretion and resistance. Am J Physiol. 1979;237(3):E214-23.
46. Frayn KN. Calculation of substrate oxidation rates in vivo from gaseous exchange. J Appl Physiol. 1983;55(2):628-34.
47. Weir JBdV. New methods for calculating metabolic rate with special reference to protein metabolism. J Physiol. 1949;109(1-2):1-9.
48. Ltd CC. Cantab 2021 [Available from: https://www.cambridgecognition.com/cantab/.
49. Division RC-HC. RAND 36-item short form health survey 1993 [Available from: https://www.rand.org/health-care/surveys_tools/mos/36-item-short-form.html.
50. Hays RD, Sherbourne CD, Mazel RM. The RAND 36-item health survey 1.0. Health Econ. 1993;2(3):217-27.
51. Cohen S, Kamarck T, Mermelstein R. A global measure of perceived stress. J Health Soc Behav. 1983;24(4):385-96.
52. van Eck M, Berkhof H, Nicolson N, Sulon J. The effects of perceived stress, traits, mood states, and stressful daily events on salivary cortisol. Psychosom Med. 1996;58(5):447-58.
53. Kromhout D, Spaaij CJ, de Goede J, Weggemans RM. The 2015 dutch food-based dietary guidelines. Eur J Clin Nutr. 2016;70(8):869-78.
54. Hernández MAG, Canfora EE, Blaak EE. Faecal microbial metabolites of proteolytic and saccharolytic fermentation in relation to degree of insulin resistance in adult individuals. Benef Microbes. 2021;12(3):259-66.
